# Modulating halide leaving-group trends through recognition by bisboranes

**DOI:** 10.1039/d5sc10013e

**Published:** 2026-02-06

**Authors:** Tong-Tong Liu, Xiao-Wen Li, Yun-Shu Cui, Zi-Hao Deng, Feng Liu, Dan-Dan Zhai, Zhang-Jie Shi

**Affiliations:** a Department of Chemistry, Fudan University Shanghai 200438 P. R. China zjshi@fudan.edu.cn zhaidandan@fudan.edu.cn liufeng@fudan.edu.cn; b State Key Laboratory of Organometallic Chemistry, Chinese Academy of Sciences Shanghai 200032 P. R. China; c Collaborative Innovation Center of Chemistry for Energy Materials (2011-iChEM) Shanghai 201418 China

## Abstract

Modulating the intrinsic leaving-group tendency of halides remains a long-standing challenge in synthetic chemistry. Herein, we reported dynamic anion recognition and demonstrated its application in nucleophilic substitution reactions, enabling an apparent reversal of the halide leaving-group tendency sequence. A bidentate Lewis acid-based platform was developed to selectively bind halide ions, forming host–guest complexes that were characterized by NMR spectroscopy and X-ray crystallography. Competitive experiments with the host molecule have revealed tunable binding affinities for the halides based on the cavity size of the bisboron center. Moreover, anion exchange experiments have demonstrated that the dynamic binding of halides is primarily influenced by their nucleophilicity and ion radius. The recognition of organohalides by diborane hosts induced a reversal of the leaving ability of Br^−^ and Cl^−^ in diborane-catalyzed transformations, which deviated from the conventional sequence.

## Abstract 2

We report highly Lewis-acidic diboranes that reversibly bind halides *via* cavity-controlled recognition, display dynamic host–guest behavior, and invert the conventional halide (Br/Cl) leaving-group order in diborane-catalyzed reactions.

## Graphical abstract

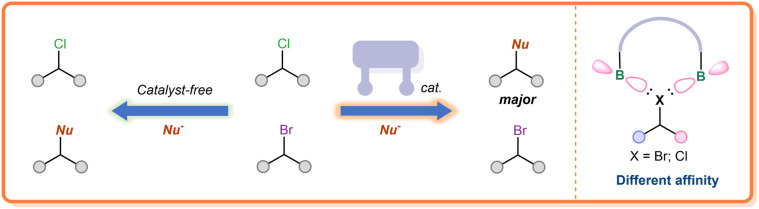

## Introduction

**Fig. 1:**
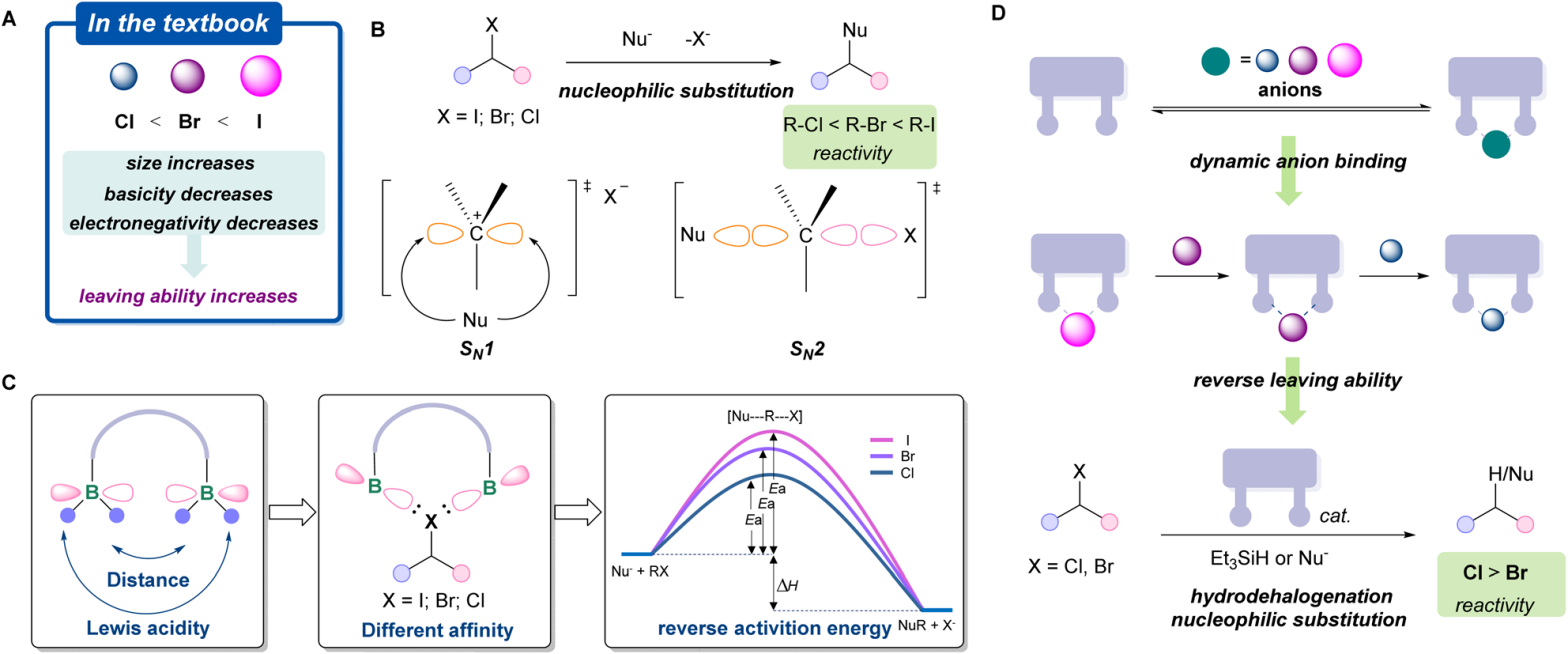
Leaving ability of organohalides. (A) The conventional leaving ability of halides; (B) the reactivity of alkyl halides in nucleophilic substitution reactions; (C) conceptual illustration of organic halides activation with diborane (activation energy using *S*_*N*_2 reaction as an example); (D) this work: dynamic binding of halogen anions and reversing leaving ability by smart recognition of halides with diborane.

Group 17 halogen anions play a pivotal role in both physiological homeostasis and modern organic synthesis.^1–4^ In synthetic chemistry, organic halides represent one of the most widely utilized building blocks, with broad applications in medicinal chemistry and materials science.^5–8^ Owing to their high electronegativity and systematic variation in atomic size, halogens impart distinct reactivity to carbon–halogen bonds, enabling nucleophilic substitution, charge-transfer processes, and transition-metal-mediated cross-coupling reactions.^9–15^ In conventional reaction systems, the leaving-group ability of halides follows a well-established hierarchy (I^−^ > Br^−^ > Cl^−^), arising from systematic trends in electronegativity, atomic size, and basicity (Fig. 1A).^16–18^ This sequence has been validated across numerous classical organic reactions, such as nucleophilic substitution (*S*_*N*_1/*S*_*N*_2) (Fig. 1B), and is widely accepted as a guiding principle in modern organic synthesis.^19^

Reversing intrinsic reactivity patterns represents a powerful strategy for expanding chemical reactivity space, as exemplified by umpolung chemistry.^20–26^ The ability to reverse the reactivity of organic halides has significant implications in a diversity of chemical transformations. To date, a general and well-developed strategy for reversing organohalide reactivity remains elusive. Boron-based poly-Lewis acids (PLAs) have recently emerged as effective platforms for anion binding *via* chelation effects.^27–32^ We envisioned that this feature may provide an alternative approach to modulate halide leaving behavior, as exemplified by *S*_*N*_2 reactions (Fig. 1C). Despite significant progress in anion binding chemistry, studies on dynamic halide binding and host–guest interactions involving diboranes remain limited, and structural evidence beyond fluoride complexes is scarce.^33^ Herein, we report a class of highly Lewis acidic diboranes capable of reversibly binding different halides through cavity-controlled recognition (Fig. 1D). Structural and spectroscopic studies reveal dynamic host–guest behavior, while catalytic investigations demonstrate that this diborane enables nucleophilic substitutions, Friedel–Crafts reactions, and reductions of alkyl chlorides and bromides with a distinct reversal of conventional halide leaving-group trends.

## Results and discussion

**Fig. 2:**
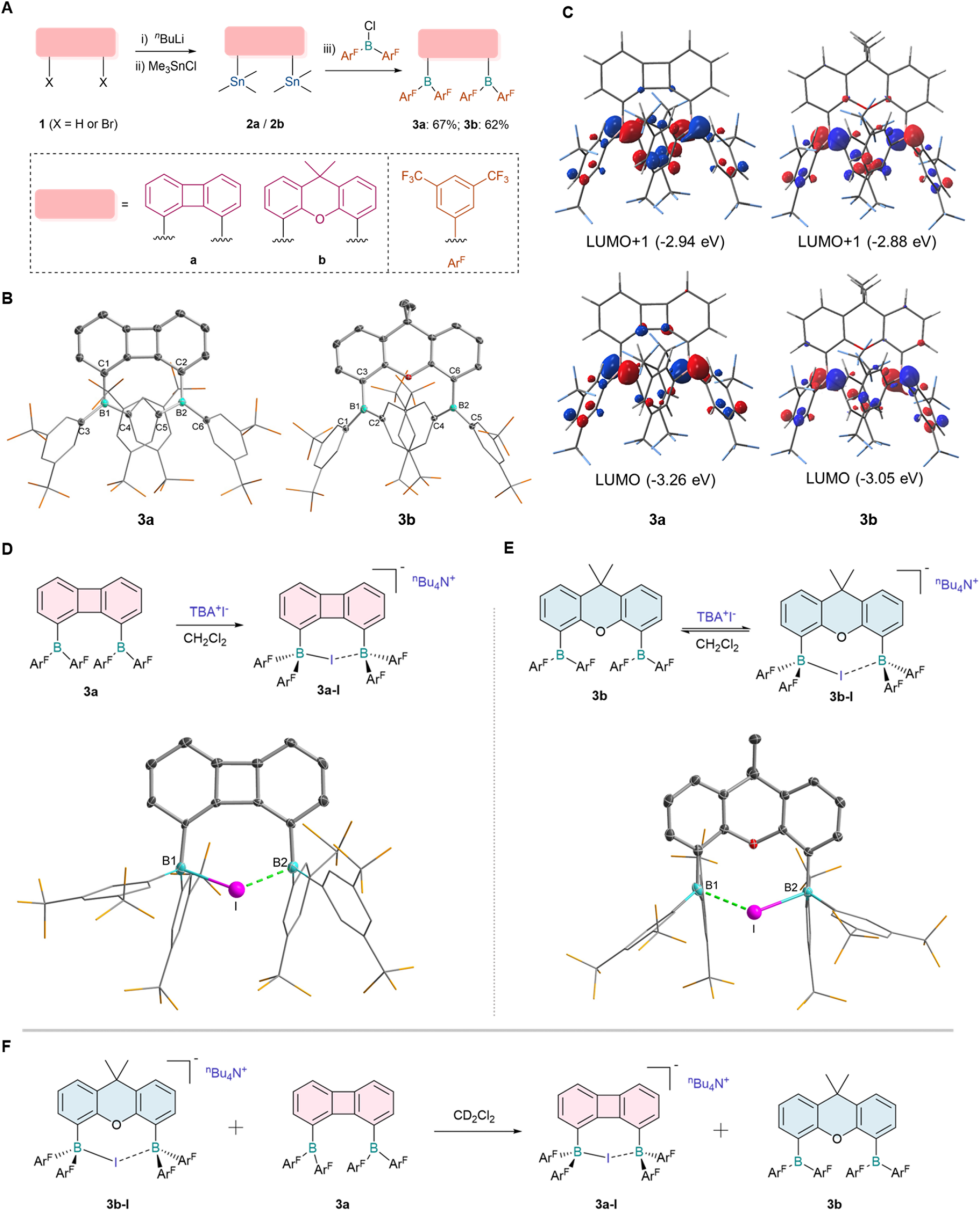
Synthesis and characterization of diboranes. (A) Synthesis of 3a and 3b. (B) Molecular structures of 3a and 3b. Thermal ellipsoids are set at the 30% probability level. Hydrogen atoms and solvent molecules are omitted for clarity. (C) Contours of frontier unoccupied molecular orbitals of 3a and 3b. Isovalue = 0.02 a.u. (D) Anion-binding of 3a with I^−^; (E) anion-binding of 3b with I^−^; (F) competitive experiments of 3a and 3b for I^−^.

We postulate that the ideal bisborane systems should possess a spacious cavity to enable the reversible chelation of anions. Working from this assumption, electron-withdrawing groups were introduced to enhance the Lewis acidity of diborane centers, while biphenylene and 9,9-dimethylxanthene frameworks were selected to ensure differential binding affinities between two borane moieties and halides.^34^ Starting with compound 1, dilithium reagents were formed *in situ* with *n*-BuLi and TMEDA (where X = H). Subsequently, Me_3_SnCl was added, resulting in high-yield formation of di-stannyl compounds 2a and 2b (Fig. 2A). Treatment of 2a with bis(3,5-bis(trifluoromethyl)phenyl)chloroborane in hexane resulted in the precipitation of an orange-red solid, which was identified as the symmetric compound 3a by ^1^H NMR and ^19^F NMR analysis. Similarly, compound 3b was synthesized, which exhibited a broad resonance at *δ* = 68.11 ppm (^11^B NMR). This downfield signal was characteristic of a three-coordinate, electron-deficient boron center and was consistent with Lewis acidic behavior. Single crystal X-ray diffraction confirmed the structures of both compounds (Fig. 2B), where the boron centers adopted a trigonal planar geometry with a sum angle for C–B–C of 360.06° (3a) and 360.08° (3b). The parallel alignment of the two diaryl boron groups effectively minimized the steric hindrance associated with 3a and 3b. As expected, the boron–boron distance in 3a (4.048 Å) was significantly lower than that in 3b (4.620 Å). Density functional theory (DFT) calculations were performed to optimize the structures of 3a and 3b. The optimized geometries matched the experimental data (Fig. 2C). The lowest unoccupied molecular orbital (LUMO) and the second lowest unoccupied molecular orbital (LUMO+1) of 3a and 3b were dominated by the two boron p_π_ orbitals and partially delocalized into the π orbitals of the fluorinated aromatic rings.

Having established the structure and electronic properties of the two diboranes, we further examined their binding affinity toward different anions. While diborane species with a large bite angle facilitated the chelation of polyatomic ions, studies addressing the synergistic chelation of monoatomic halide ions remained scarce.^35,36^ Synergistic chelation of monoatomic halide ions is geometrically disfavored by the relatively large boron–boron separation, however, this spatial constraint may be exploited to enable dynamic anion binding.^37,38^ The introduction of high Lewis acid-type boron substituents may compensate this spatial barrier. Unlike fluoride anions (F^−^), iodide anions (I^−^) exhibit considerably weaker binding affinity toward diboranes and have thus rarely been investigated. Therefore, we set out to investigate the interaction between iodide anions and diboranes 3a or 3b (Fig. 2). Upon adding TBAI to a dichloromethane solution of 3a, the color quickly faded and the ^1^H NMR spectrum revealed a set of new symmetric peaks, indicating that two sets of boryl substituents were equivalent in solution. Colorless single crystals suitable for X-ray crystallographic studies were obtained by recrystallization from a dichloromethane and *n*-hexane solution, confirming the structure of product 3a-I (Fig. 2D). In the solid state, iodide ions were chelated by both boron centers, where one boron center was slightly closer to the iodide anion. The B–I bond lengths were 2.407(6) Å(B1–I) and 2.469(6) Å(B2–I), respectively. This coordination mode with I^−^ was also observed in diborane 3b, although a coordination dissociation equilibrium was observed in solution (Fig. 2E). The single crystal of complex 3b-I was obtained under low temperature conditions and the chelating coordination structure was determined by X-ray diffraction analysis. The boron–boron bond distance (B1–B2, 4.40 Å) was smaller than that in precursor 3b. The B–I bond lengths were determined to be 2.668(3) Å and 2.467(3) Å, respectively, with the resultant bond length discrepancy being more pronounced than that observed in 3a-I, which was indicative of a weaker chelation interaction. Subsequent to the synthesis and detailed structural characterization of these complexes, the anion-binding capabilities of the two diborane derivatives were evaluated *via* competitive binding experiments. Upon mixing 3a and 3b-I, ^1^H NMR spectroscopic monitoring revealed the quantitative transfer of I^−^ from 3b-I to 3a, forming 3a-I and free 3b (Fig. 2F and S34), demonstrating that the cavity size of the boron moiety had a crucial influence on the binding affinity toward I^−^.

**Fig. 3:**
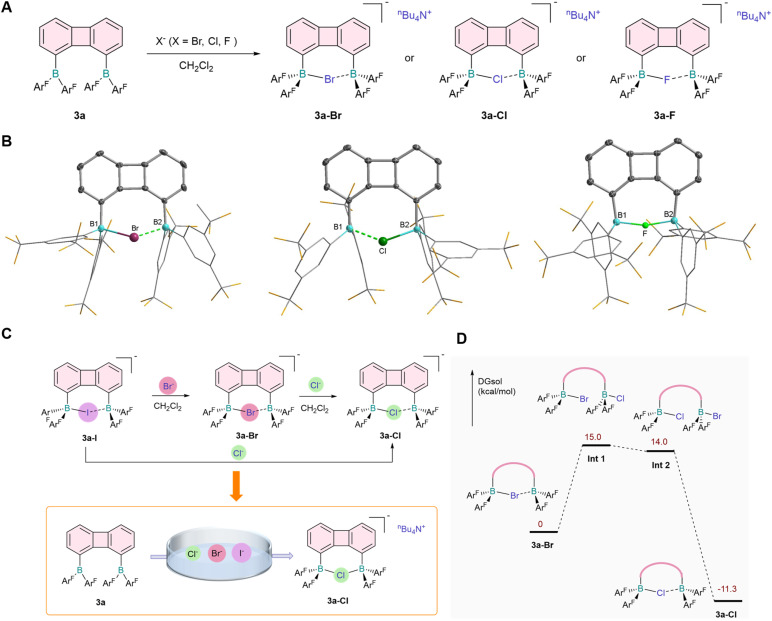
Studies on the affinity of halide anions. (A) Anion-binding studies of 3a with halide anions. (B) Molecular structure of 3a-Br, 3a-Cl and 3a-F. Thermal ellipsoids are set at the 30% probability level. Hydrogen atoms and solvent molecules are omitted for clarity. (C) Competitive experiments of various anions with 3a. (D) Proposed mechanism of the Cl^−^/Br^−^ exchange by DFT calculation.

Given the higher affinity of 3a for I^−^, we chose 3a as a host in other halide binding studies. As expected, when stronger nucleophilic halide ions (Br^−^, Cl^−^ and F^−^) reacted with 3a, the corresponding complexes (3a-Br, 3a-Cl and 3a-F) with anions chelated at bisboron centers were obtained (Fig. 3A). Based on periodic trends, an increase in atomic number within the same group gave rise to a larger ionic radius, which enabled halide ions to be more readily accommodated by diboranes with larger bite angles. The boron–boron bond distances exhibited a gradual decrease in the order of 3a-I < 3a-Br < 3a-Cl < 3a-F (Table 1), which was indicative of distinct interaction strengths between the bisboron moieties and halide ions. In addition, the B–X interactions were regulated by electrostatic effects, as evidenced by the single-crystal structural data (Fig. 3B), where the B–X bond lengths for Br^−^, Cl^−^ and F^−^ were determined to be 2.221(4), 2.056(2) and 1.914(4) Å, respectively. In comparison with the reported monomeric borane–halide adducts,^39–41^ the B–X bond lengths in these bisboron complexes were slightly elongated; this phenomenon was consistent with the chelation-assisted halide binding mode, in which the halide ions were coordinatively shared between two proximal boron centers.

**Table 1:** Selected bond lengths and distances (Å)

	3a-I	3a-Br	3a-Cl	3a-F^a^
B1⋯B2	4.040(8)	3.882(7)	3.736(3)	3.354(5)^α^, 3.264(3)^β^
B1-X	2.407(6)	2.221(4)	2.056(2)	1.914(4)^α^, 1.642(3)^β^
B2-X	2.469(6)	2.314(5)	2.214(2)	1.623(3)^α^, 1.656(3)^β^

a Two coordination configurations occur in a unit cell.

Based on the experimental results, we confirmed that 3a was capable of synergistically chelating different halogen anions, providing a basis for dynamic anion binding studies. Host–guest complexation chemistry of 3a was then explored (Fig. 3C), with Br^−^ employed to study competitive binding against I^−^. We found that 3a-I quantitatively converted to the 3a-Br complex within seconds upon the addition of Br^−^ (see ESI, Fig. S35), demonstrating a higher affinity of the diborane 3a for Br^−^. Analogously, competitive binding experiments with Cl^−^ and 3a-I also resulted in the formation of 3a-Cl (Fig. S37). Competitive binding experiments between Br^−^ and Cl^−^ further revealed the predominant generation of 3a-Cl (Fig. S36), showing the superior affinity of diborane 3a for Cl^−^ relative to Br^−^.

To elucidate the anion exchange mechanism, we performed density functional theory (DFT) calculations and obtained the corresponding energy profile, using the Cl^−^/Br^−^ exchange as a model system (Fig. 3D). An associative substitution pathway was proposed herein: Cl^−^ first coordinated to one boron center of the 3a-Br adduct, leading to the formation of a dianionic intermediate; subsequent chelation of Cl^−^ and terminal binding of Br^−^ then occurred, a process facilitated by the rotation of the two C_aryl_–B single bonds. The exchange was completed followed by desorption of Br^−^. Notably, the addition of F^−^ to a dichloromethane solution of 3a-Cl caused severe disruption of the ^1^H NMR signals (Fig. S38), indicating the formation of cross-multi-coordinated complexes. This reduced chelation selectivity of 3a toward Cl^−^ and F^−^ could be rationalized by combined comparable effects of anion ionic radius and charge density in their interactions with the boron centers of 3a. Furthermore, mixed competitive binding experiments in the presence of Cl^−^, Br^−^ and I^−^ confirmed that 3a exhibited prominent chelation selectivity for Cl^−^ (Fig. 3C and S39), a preference attributable to the well-matched bisboron cavity and favorable electrostatic interactions between Cl^−^ and the boron centers.

**Fig. 4:**
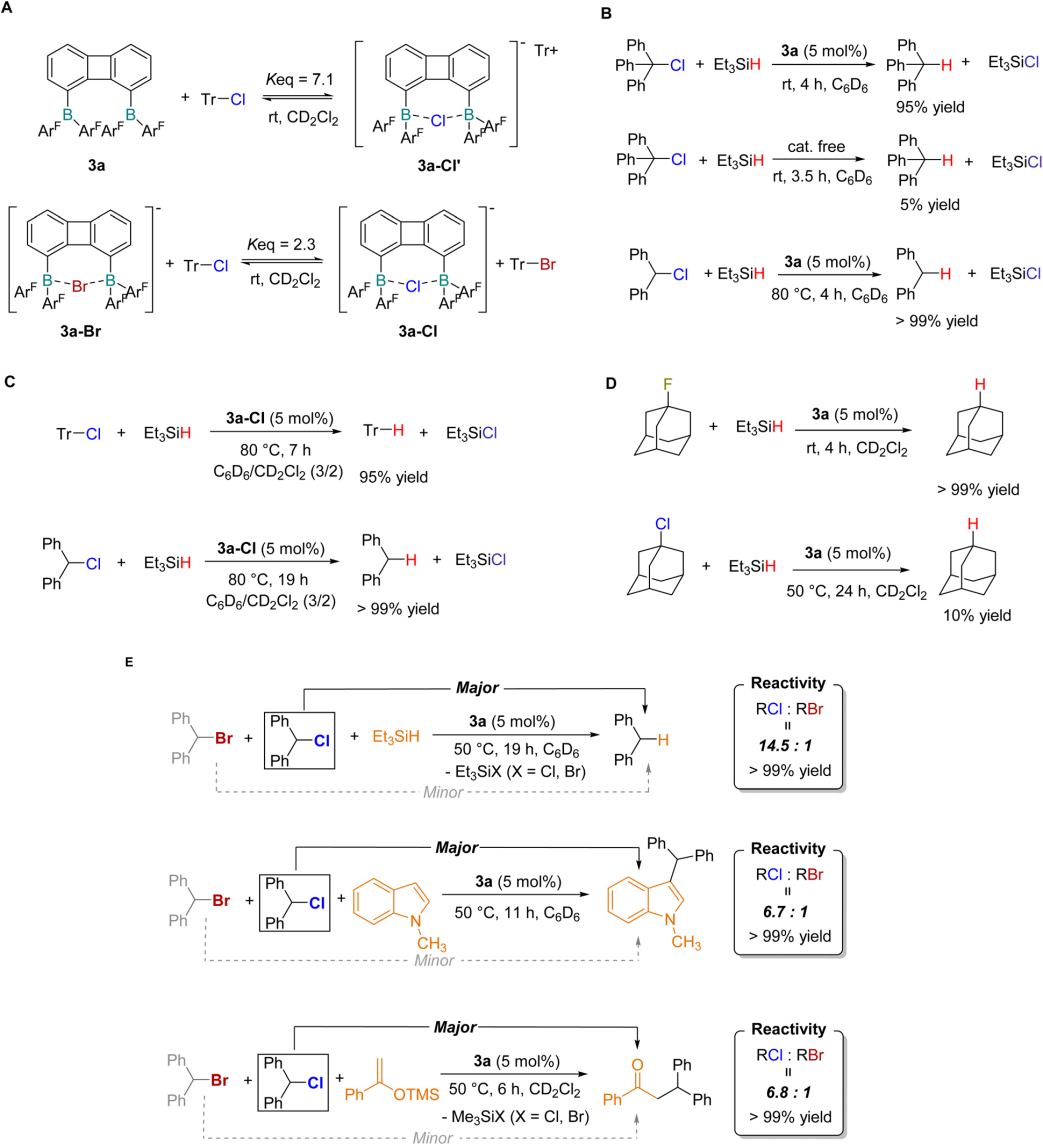
Reverse leaving ability of halides by diborane 3a. (A) C–Cl bond cleavage in trityl chloride and halogen exchange reaction; (B) catalytic hydrodechlorination of trityl chloride and diphenylchloromethane; (C) control experiments catalyzed by diborane-chloride adduct 3a-Cl; (D) catalytic hydrodehalogenation of 1-AdF/1-AdCl by 3a; (E) reversal of the leaving group ability of halides in bisborane catalyzed transformations. *Reaction yields in panels (B–E) were determined by ^1^H NMR spectroscopy.

Furthermore, we investigated the reactivity of 3a toward covalent C-X bonds. Upon reaction of 3a with Ph_3_CCl in CD_2_Cl_2_ at room temperature, complex 3a-Cl′ was formed and the system reached an equilibrium state (*K*_eq_ = 7.1), indicating the reversible activation of the C–Cl bond by 3a (Fig. 4A and S40). Analogously, the equilibrium reaction of 3a-Br with Ph_3_CCl yielded 3a-Cl as the anion exchange product (Fig. 4A and S42). This chelation equilibrium suggested a potential catalytic capability of 3a in C–X bond cleavage. We thus proposed a catalytic pathway where diborane 3a facilitated the reduction of C–X bonds by quenching of carbon cations with a weak hydride source. Experimental results confirmed this hypothesis: triphenylmethane was obtained in excellent yield when triethylsilane was employed as the reductant in the presence of a catalytic amount of 3a (Fig. 4B). When Ph_2_CHCl was used as the substrate, the reaction proceeded at a lower rate at room temperature, and complete conversion was achieved within 3 h upon heating to 80 °C. Control experiments demonstrated that these reactions did not occur in the absence of diborane catalysts. To further elucidate the role of the diborane 3a, we tested the catalytic performance of the 3a–Cl adduct in the reduction of C–Cl bonds with silanes, and the corresponding reduced products were obtained in high yields (Fig. 4C). These results suggested that diborane 3a may act as a catalyst rather than an initiator in the catalytic cycle. In addition, we investigated the reduction of the 1-fluoroadamantane using compound 3a as a catalyst at room temperature, and adamantane was quantitatively produced (Fig. 4D). In contrast, 1-chloroadamantane could not be effectively reduced, even under heating conditions.

Based on the results of halide affinity competition experiments and catalytic reactions described above, we proposed that diborane species could induce a reversal in the leaving group ability of halides in catalytic transformations. To demonstrate this proof of concept, three representative transformations of organohalides were evaluated to assess the selective activation of organic halides by bisboron catalysts (Fig. 4E). First, Ph_2_CHCl and Ph_2_CHBr were used to determine the selectivity of diborane-catalyzed reduction with silanes. The hydrogenated product Ph_2_CH_2_ was obtained in high efficiency in the presence of triethylsilane (1.0 equiv.) and catalyst 3a (5 mol%) at 50 °C, with the chloro-substituted substrate displaying significantly higher reactivity than its bromo congener (reactivity ratio = 14.5 : 1). Under analogous reaction conditions with *N*-methylindole (1.0 equiv.) as the substrate, the Friedel–Crafts alkylation also exhibited a preference for Ph_2_CHCl over the bromo analogue (reactivity ratio = 6.7 : 1). This reversed halide selectivity was further retained in bisboron-catalyzed nucleophilic substitution reactions with an enol silyl ether, albeit with a moderate reactivity ratio of 6.8 : 1. The relatively modest selectivity observed in the nucleophilic substitution reaction was attributed to the presence of competing reaction pathways, including direct nucleophile participation and dynamic halide exchange at the bisboron center, which partially diminished the catalyst-controlled halide recognition process. Control experiments confirmed that a small amount of product formation occurred in the absence of catalyst 3a (Fig. S57 and S62). Additionally, a monoboron analogue (Fxyl)_2_BPh (3c) was synthesized, and under otherwise identical reaction conditions, it exhibited a substantial reduction in Cl/Br selectivity (Fig. S63–S66). Collectively, these findings demonstrated that the rational design of the bisboron framework enabled the reversal of halide leaving group reactivity, thus providing a versatile strategy for the precise control of reaction selectivity in organohalide transformations.

## Conclusions

In conclusion, we have developed a class of bidentate boron-based strong Lewis acids featuring a large bite angle, which enables chelation with monoatomic halide anions. These diboranes exhibit tunable and dynamic halide-binding behavior. Importantly, the prominent anion recognition ability of 3a has been successfully applied to the catalytic transformation of organohalides, achieving a reversal of the conventional reactivity of C–X bonds. These findings establish anion recognition as a promising strategy for modulating organohalide reactivity and provide valuable insights for the rational design of host-based catalytic systems for selective bond activation.

## Author contributions

L. T.-T. and S. Z.-J. directed the research and developed the concept of the reaction. L. T.-T. and L. X.-W. designed and carried out the experiments and characterized the products. C. Y. S. performed the computation. D. Z.-H. performed the data collection for some complexes. L. T.-T., L. X.-W., L. F., Z. D.-D., and S. Z.-J. analysed data and prepared the manuscript.

## Conflicts of interest

There are no conflicts to declare.

## Supplementary Material

SC-017-D5SC10013E-s001

SC-017-D5SC10013E-s002

## Data Availability

CCDC 2323469 (3a), 2323477 (3b), 2323472 (3a-I), 2323470 (3b-I), 2323475 (3a-F), 2323474 (3a-Cl), 2323473 (3a-Br) contains the supplementary crystallographic data for this paper.^42*a*–*g*^ The data supporting this article have been included as part of the supplementary information (SI). Supplementary information is available. See DOI: https://doi.org/10.1039/d5sc10013e.
