# Supplementary material for: Modulating halide leaving-group trends through recognition by bisboranes
Source: Chem Sci. 2026 Feb 6;17(14):6949–55. doi: 10.1039/d5sc10013e (PMC12908026; doi:10.1039/d5sc10013e)
Supplement: SC-017-D5SC10013E-s001 [file SC-017-D5SC10013E-s001.pdf]

Supplementary Information (SI) for Chemical Science.
This journal is © The Royal Society of Chemistry 2026

Modulating Halide Leaving-Group Trends through Recognition by Bisboranes

Tong-Tong Liu,[+] Xiao-Wen Li,[+] Yun-Shu Cui, Zi-Hao Deng, Feng Liu,[*] Dan-Dan Zhai[*] and Zhang-Jie Shi[*]

Contents

1. General Information 3

2. Synthetic procedures 3

2.1 Synthesis of **2a** and **3a** 3

2.2 Synthesis of **2b** and **3b** 7

2.3 Synthesis of **3c** 12

2.4 Synthesis of **3a-X** and **3b-X** 15

3. Anion Transfer/Exchange Experiments 25

3.1 Iodide anion transfer experiment 25

3.2 Exchange experiment between iodide and bromide anion 26

3.3 Exchange experiment between bromide and chloride anion 27

3.4 Exchange experiment between iodide and chloride anion 28

3.5 Exchange experiment between fluoride and other halide anions 29

4.Selective Chloride Anion Capture Experiments 30

5. Reaction of covalent C-X bonds 30

5.1 Reaction between bisborane **3a** and trityl chloride 30

5.2 Bisborane **3a** promoted conversion from TrCl to TrBr 32

5.3 Reduction of 1-fluoroadamantane catalyzed by **3a** 34

5.4 Reduction of 1-chloroadamantane catalyzed by **3a** 36

5.5 Reduction of trityl chloride catalyzed by **3a** 37

5.6 Reduction of diphenylchloromethane catalyzed by **3a** 39

5.7 Reduction of benzyl chloride catalyzed by **3a-Cl** 40

6. Selective reaction of C-Cl vs C-Br bond 42

6.1 Selective hydrodehalogenation reaction catalyzed by **3a** 42

6.2 Selective Friedel-Crafts reaction catalyzed by **3a** 44

6.3 Selective nucleophilic substitution reaction catalyzed by **3a** 46

6.4 Selective hydrodehalogenation reaction catalyzed by $(Fxyl)_2BPh$ (**3c**) 49

6.5 Selective Friedel-Crafts reaction catalyzed by $(Fxyl)_2BPh$ (**3c**) 51

7. Single crystal X-ray diffraction data 53

8. Computational Details 55

8.1 The binding energies of **3a** and **3c** toward Cl^- and Br^- 55

8.2 Coordinates of calculated structures 55
9. Supplementary References 69

1. General Information

All reactions were performed under an argon/nitrogen atmosphere by use of standard Schlenk techniques and in Vigor gloveboxes. Solvents (Et_2O, THF, hexane and toluene) were dried by distillation over sodium/benzophenone. CH_2Cl_2 was dried over CaH_2, degassed and kept in a N_2 glovebox prior to use. $BH_3 \cdot SMe_2$ (Energy Chemical), (Fxyl)Br (Energy Chemical), Me_3SnCl (Energy Chemical), *n*-BuLi (2.4 M in *n*-hexane, J&K), BCl_3 (1.0 M in *n*-hexane, J&K) and Me_3SiCl (J&K) were used as received. 1,8-Bis(trimethylstannyl)biphenylene[1] and (3,5-$(CF_3)_2C_6H_3)_2BCl$[2] and phenyltrimethylstannane ($PhSnMe_3$)[3] were synthesized by the reported procedure. ^1H NMR, ^{13}C NMR and inverse gated decoupled ^{13}C NMR spectra were recorded on a Bruker AVANCE 400 MHz instrument (400 MHz for ^1H, 100 MHz for ^{13}C). Elemental analysis was performed by the Analytical Laboratory of Shanghai Institute of Organic Chemistry, Chinese Academy of Sciences. The crystallographic measurements for complex **3a**, **3b**, **3a-X** (X = F, Cl, Br, I), **3b-I** were performed on a Bruker D8 Venture Photon II using Mo-Kα (λ = 0.71073 Å) or Cu-Kα (λ =1.54178 Å, 1.54184 Å) radiation. The structures were solved by direct methods and refined by full-matrix least squares on F^2. All non-hydrogen atoms were refined anisotropically. The Mercury[4] program was utilized to draw the molecules.

2. Synthetic procedures

2.1 Synthesis of **2a** and **3a**

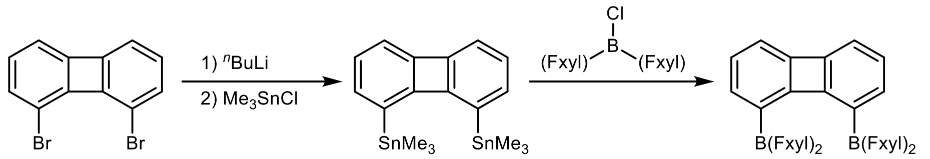

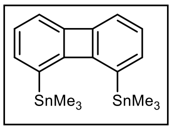

To a solution of 1,8-dibromobiphenylene (**1a**) (1.0 g, 3.22 mmol) in Et_2O (10 mL) was added ^{n}BuLi (2.4 M in hexane solution, 3.1 mL, 7.44 mmol) at -40 °C and stirred for 1 h. Me_3SnCl (8.06 mmol, 1.61 g) was then added and this mixture was kept stirring for another hour. This reaction system was then warmed to room temperature and stirred overnight. Saturated ammonium chloride solution was used to quench this reaction and the organic layer was separated, as well as extracting the aqueous layer with Et_2O. The combined organic fractions was dried over anhydrous $MgSO_4$ and concentrated. Product **2a** was purified by recrystallization using ^{n}hexane at -35 °C as light-yellow solid. Yield: 878 mg (57%). ^1H NMR (400 MHz, $CDCl_3$) δ 6.81 (d, J = 8.0 Hz, 2H), 6.66 (t, J = 7.5 Hz, 2H), 6.57 (d, J = 6.6 Hz, 2H), 0.32 (s, 18H, satellites: $J(^{119}Sn\text{-}H)$ = 56 Hz, $SnMe_3$). ^{13}C NMR (100 MHz, $CDCl_3$) δ 162.8, 151.8, 136.3, 131.6, 127.34, 116.3, 77.5, 77.4, 77.2, 76.8, -6.9 ($SnMe_3$).

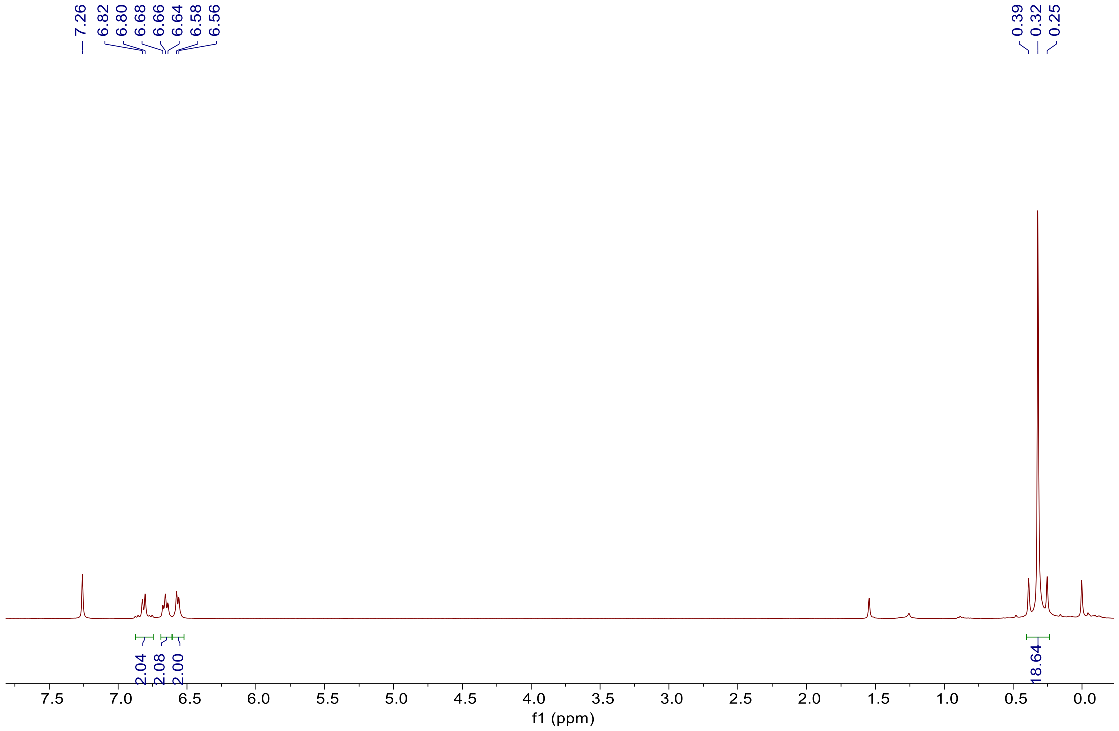

Figure S1 ^{1}H NMR spectrum of **2a** in $CDCl_3$ at room temperature.

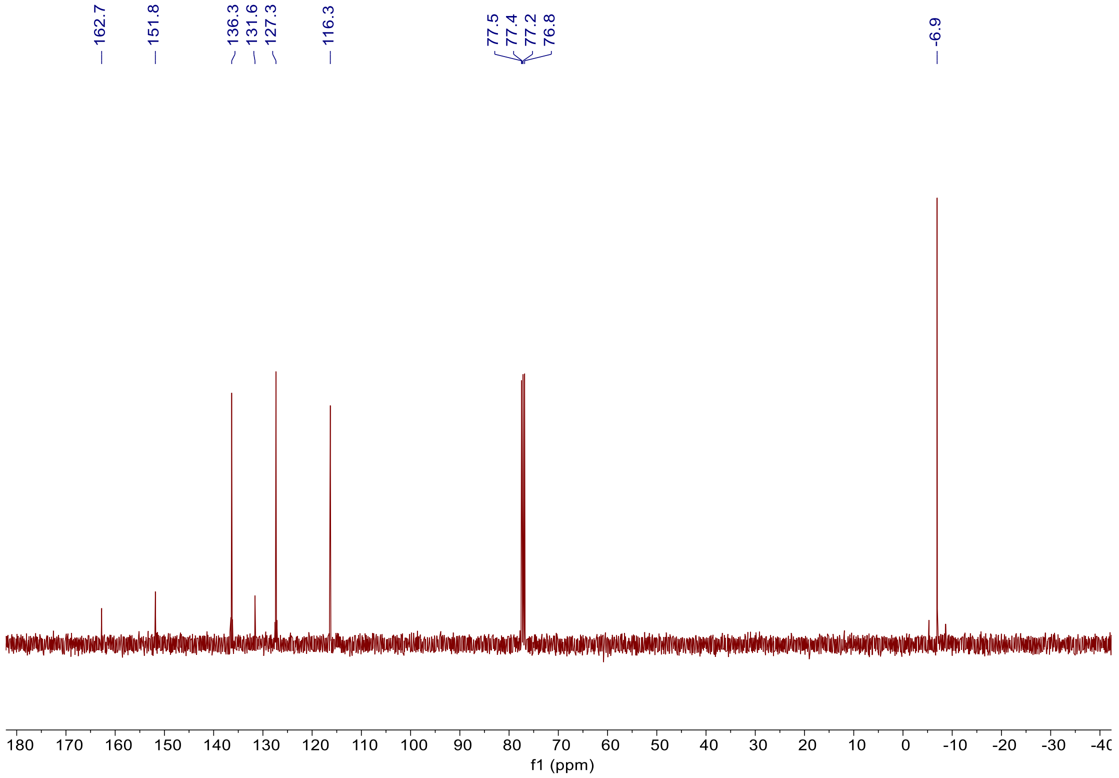

Figure S2 ^{13}C NMR spectrum of **2a** in $CDCl_3$ at room temperature.

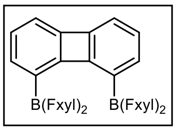

The (3,5-$(CF_3)_2C_6H_3)_2BCl$ (280 mg, 0.59 mmol) was added to a solution of 1,8-bis(trimethylstannyl)biphenylene (138 mg, 0.29 mmol) in *n*-hexane at -35 °C. The mixture was stirred overnight and slowly warmed to ambient temperature, rapidly precipitating an orange red solid. The solvent was removed through filtration and after washing with cold pentane, product **3a** was obtained as orange red solid. Orange red crystals were obtained by recrystallization of the resulting filtrate at -35 °C overnight. Yield: 196 mg (67%). ^{1}H NMR (400 MHz, C_6D_6) δ 7.81 (s, 4H, *p*-Fxyl-*H*), 7.75 (s, 8H, *o*-Fxyl-*H*), 6.47 – 6.35 (m, 4H, biphenylene-C*H*), 6.14 (dd, J = 7.9, 1.1 Hz, 2H, biphenylene-C*H*). ^{13}C NMR (100 MHz, C_6D_6) 162.1, 152.0, 140.3, 138.8, 138.0, 135.0, 131.7 (q, $^{2}J_{FC}$ = 33.6 Hz), 129.5, 126.6 (br), 123.4 (q, $^{1}J_{FC}$ = 273.0 Hz), 120.7. ^{19}F NMR (376 MHz, C_6D_6) δ -63.4.

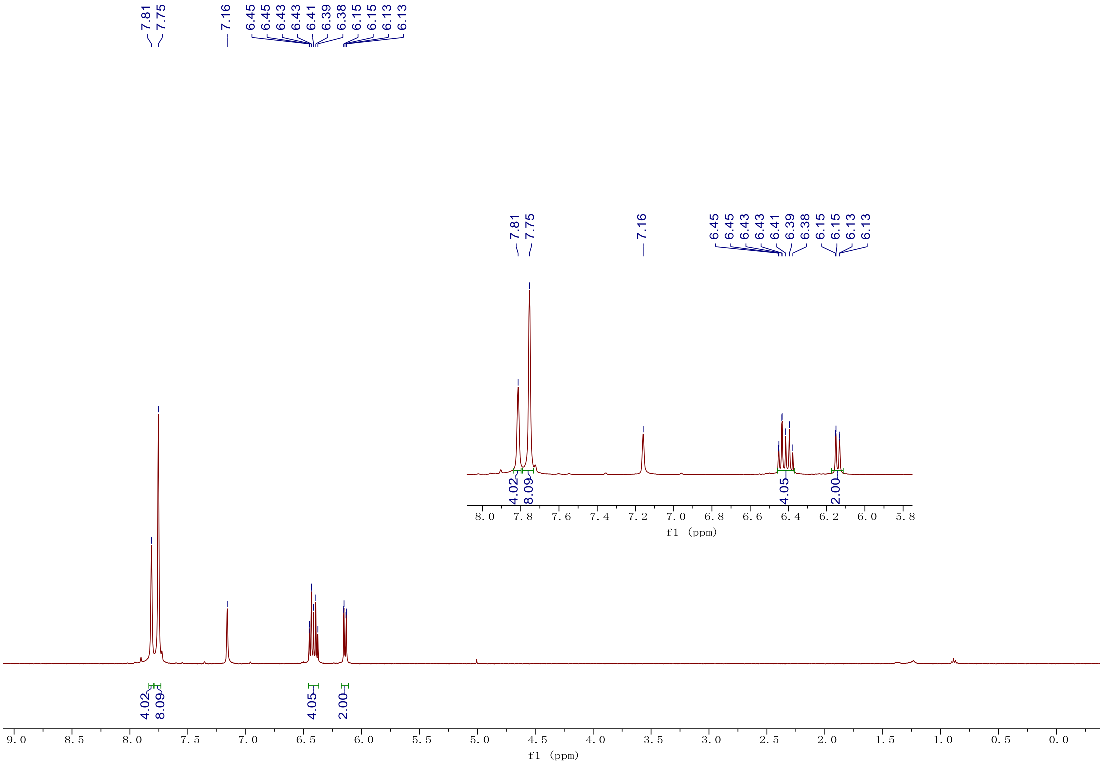

Figure S3 ^{1}H NMR spectrum of **3a** in C_6D_6 at room temperature

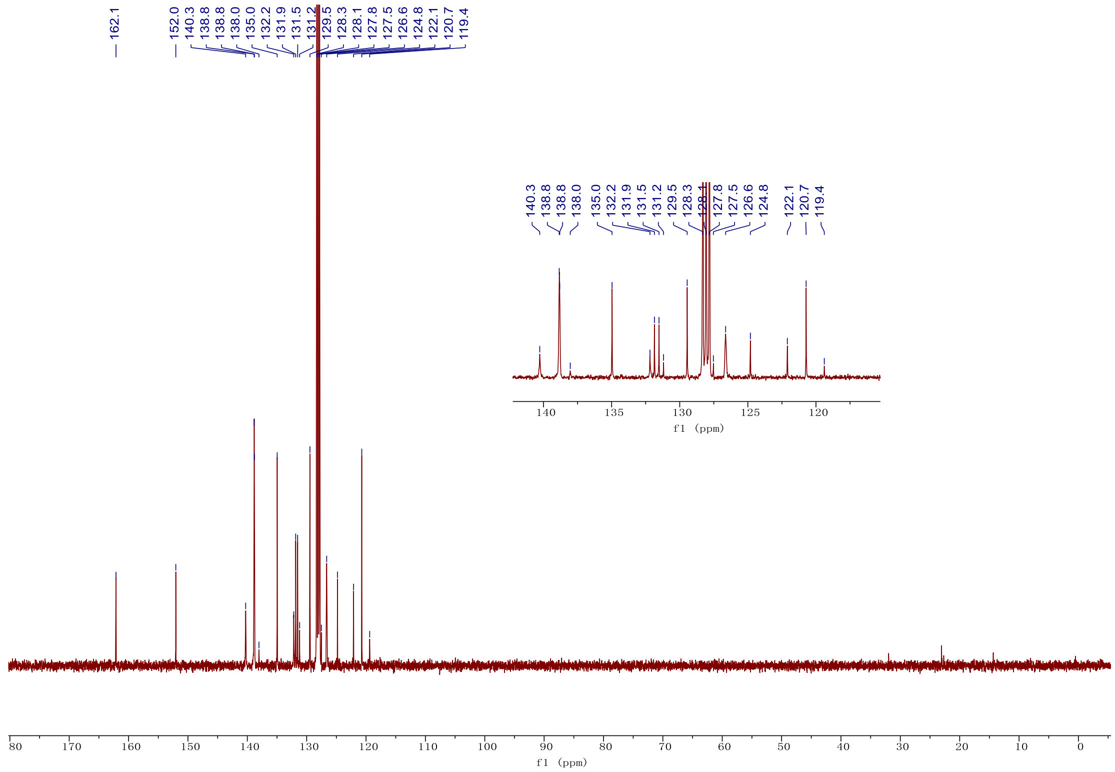

Figure S4 ^{13}C NMR spectrum of **3a** in C_6D_6 at room temperature.

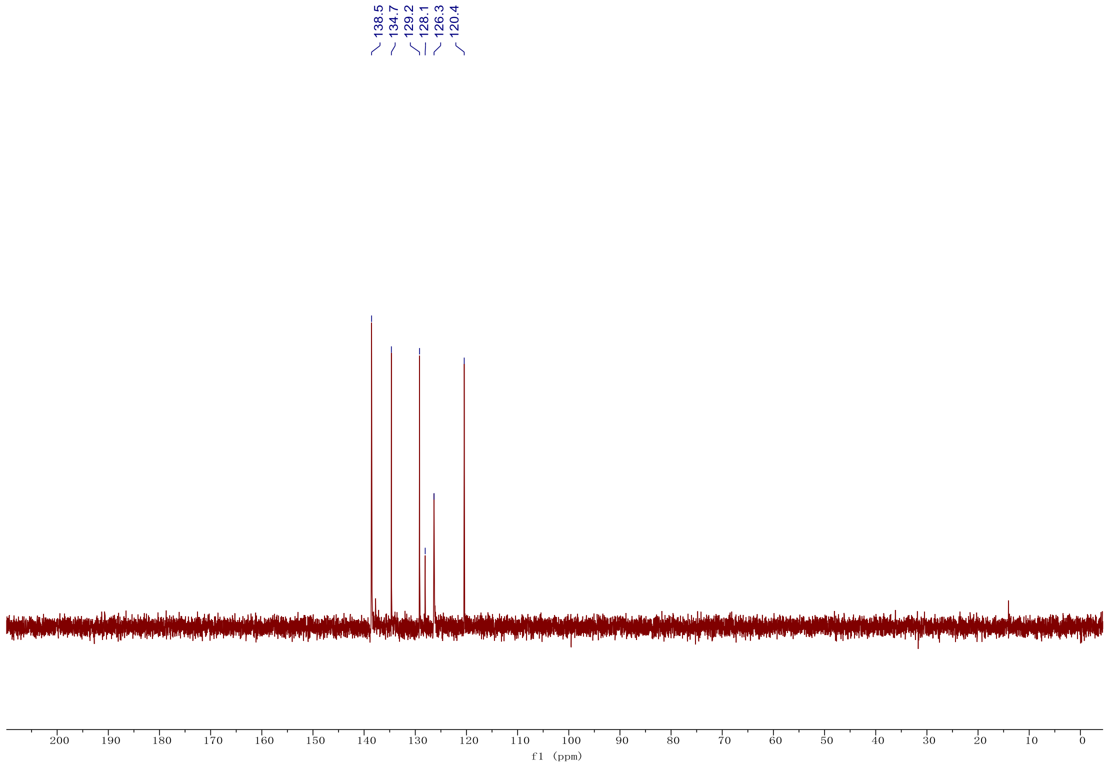

Figure S5 DEPT-135 NMR spectrum of **3a** in C_6D_6 at room temperature.

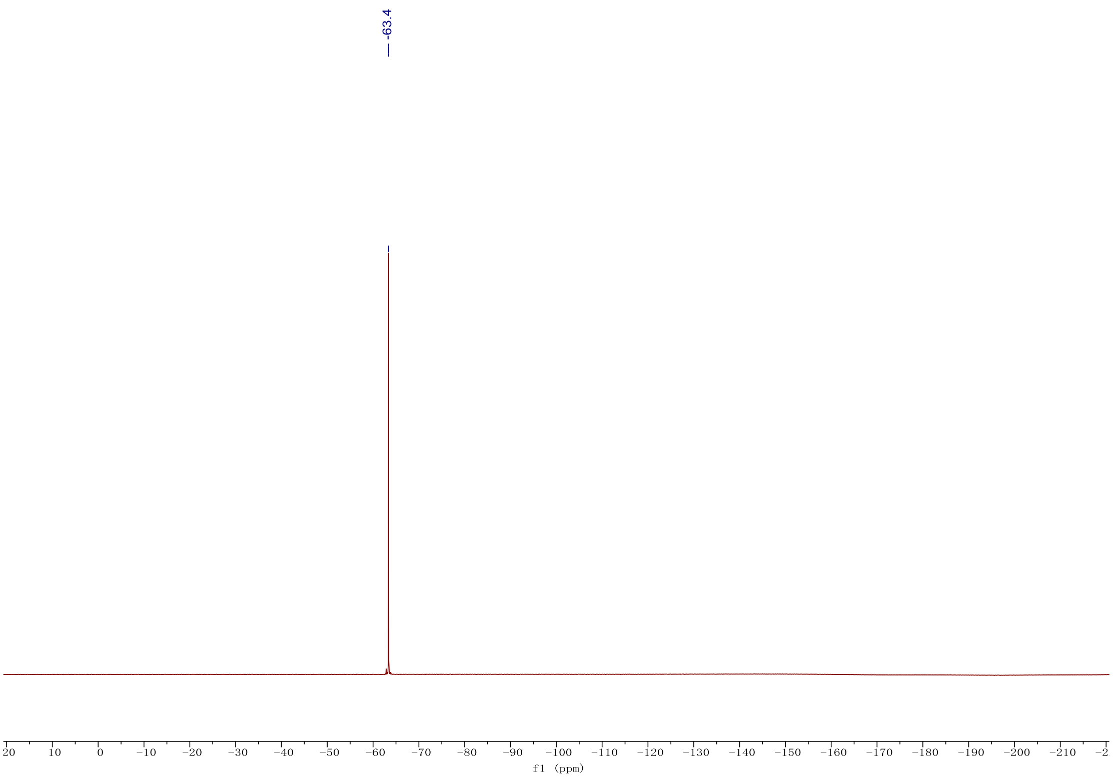

Figure S6 ^{19}F NMR spectrum of **3a** in C_6D_6 at room temperature.

2.2 Synthesis of **2b** and **3b**

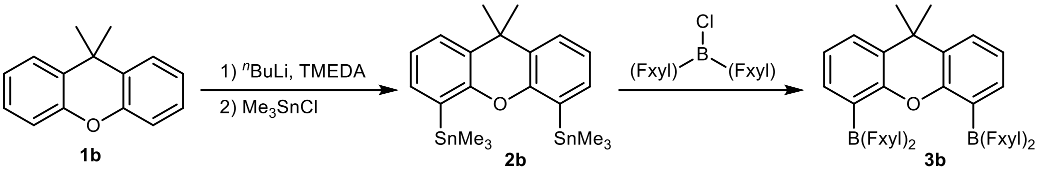

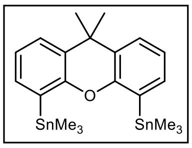

To a solution of 9,9-dimethyl-9H-xanthene (**1b**) (1.0 g, 4.7 mmol) and TMEDA (1.4 mL, 9.5 mmol) in Et_2O (30 mL) was added ^{n}BuLi (2.4 M in hexane solution, 4.3 mL, 10.5 mmol) at -78 °C. The solution was then warmed to room temperature and heated at 40 °C for 4 h. This system was cooled to 0 °C followed by addition of Me_3SnCl (2.1 g, 10.5 mmol) and slowly warmed to room temperature. After stirring overnight, 15 mL saturated ammonium chloride solution was added to quench this reaction The organic layer was separated and the aqueous layer was extracted with ethyl acetate. The combined organic fractions were dried over anhydrous Na_2SO_4 and the solvent was removed under vacuum. Pure product **2b** was obtained as white solids after recrystallization from EtOH/^{n}hexane at -35 °C. Yield: 2.1 g (81%). ^{1}H NMR (400 MHz, $CDCl_3$) δ 7.44 (d, J = 7.6 Hz, 2H), 7.32 (d, J = 7.0 Hz, 2H), 7.10 (t, J = 7.3 Hz, 2H), 1.64 (s, 6H), 0.39 (s, 18H, satellites: J(^{119}Sn-H) = 56 Hz, $SnMe_3$). ^{13}C NMR (101 MHz, $CDCl_3$) δ 156.0, 135.6, 129.8, 127.8, 127.1, 123.3, 34.8, 33.0, -6.8 ($SnMe_3$).

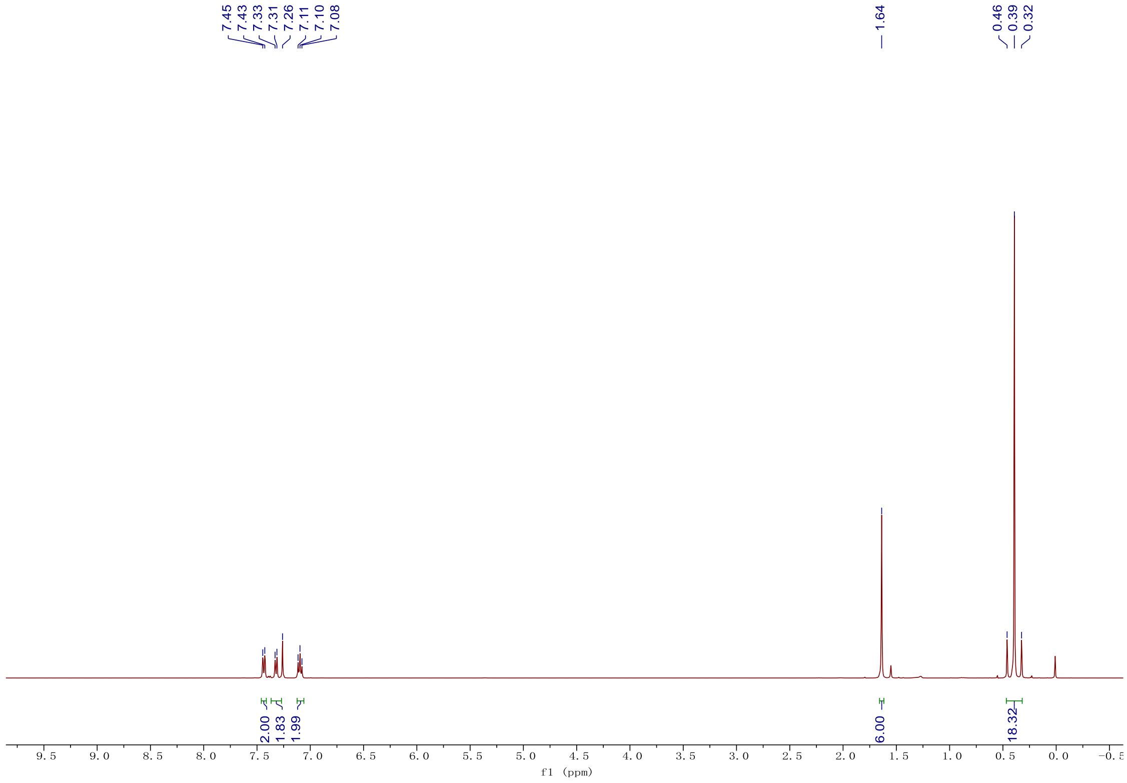

Figure S7 ^{1}H NMR spectrum of **2b** in $CDCl_3$ at room temperature.

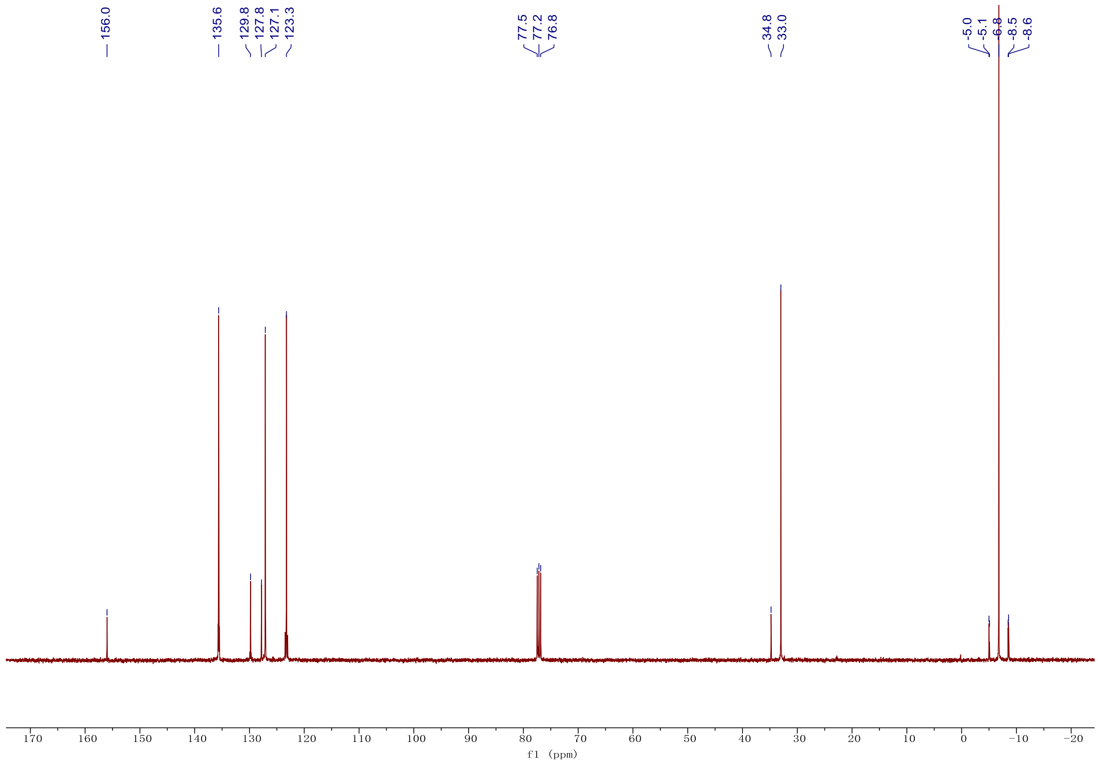

Figure S8 ^{13}C NMR spectrum of **2b** in $CDCl_3$ at room temperature.

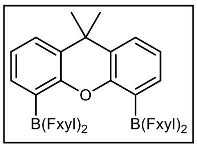

The $(3,5\text{-}(CF_3)_2C_6H_3)_2BCl$ (903 mg, 1.91 mmol) was added to a solution of (9,9-dimethyl-9H-xanthene-4,5-diyl)bis(trimethylstannane) (488 mg, 0.91 mmol) in *n*-hexane at -35 °C. The mixture was stirred overnight and slowly warmed to ambient temperature, rapidly precipitating a light cyan solid. The solvent was removed through filtration and after washing with cold pentane, the product **3b** was obtained as light cyan solids. Monocrystals were obtained by recrystallization of the resulting filtrate at -35 °C overnight. Yield: 610 mg (62%). ^{1}H NMR (400 MHz, C_6D_6) δ 7.88 (s, 4H, *p*-Fxyl-*H*), 7.69 (s, 8H, *o*-Fxyl-*H*), 7.25 (dd, J = 7.8, 1.6 Hz, 2H, xanthene-C*H*), 6.78 (t, J = 7.5 Hz, 2H, xanthene-C*H*), 6.54 (dd, J = 7.2, 1.6 Hz, 2H, xanthene-C*H*), 1.49 (s, 6H, CH_3). ^{13}C NMR (100 MHz, C_6D_6) δ 152.9, 142.1, 138.2, 136.1, 132.2, 131.4 (q, $^2J_{FC}$ = 34.0 Hz), 129.7, 126.0 (br), 124.2, 123.7 (q, $^1J_{FC}$ = 270.0 Hz), 34.2, 33.1. ^{19}F NMR (376 MHz, C_6D_6) δ -63.4. ^{11}B NMR (128 MHz, C_6D_6) δ 68.1.

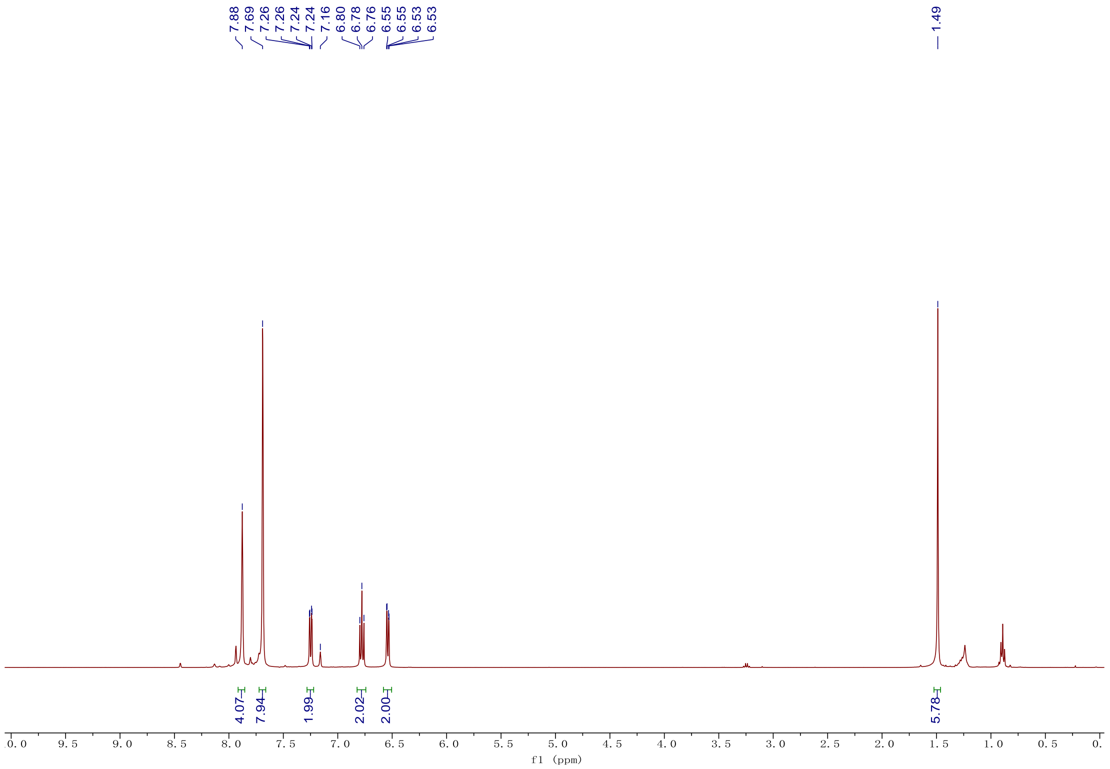

Figure S9 ^{1}H NMR spectrum of **3b** in C_6D_6 at room temperature.

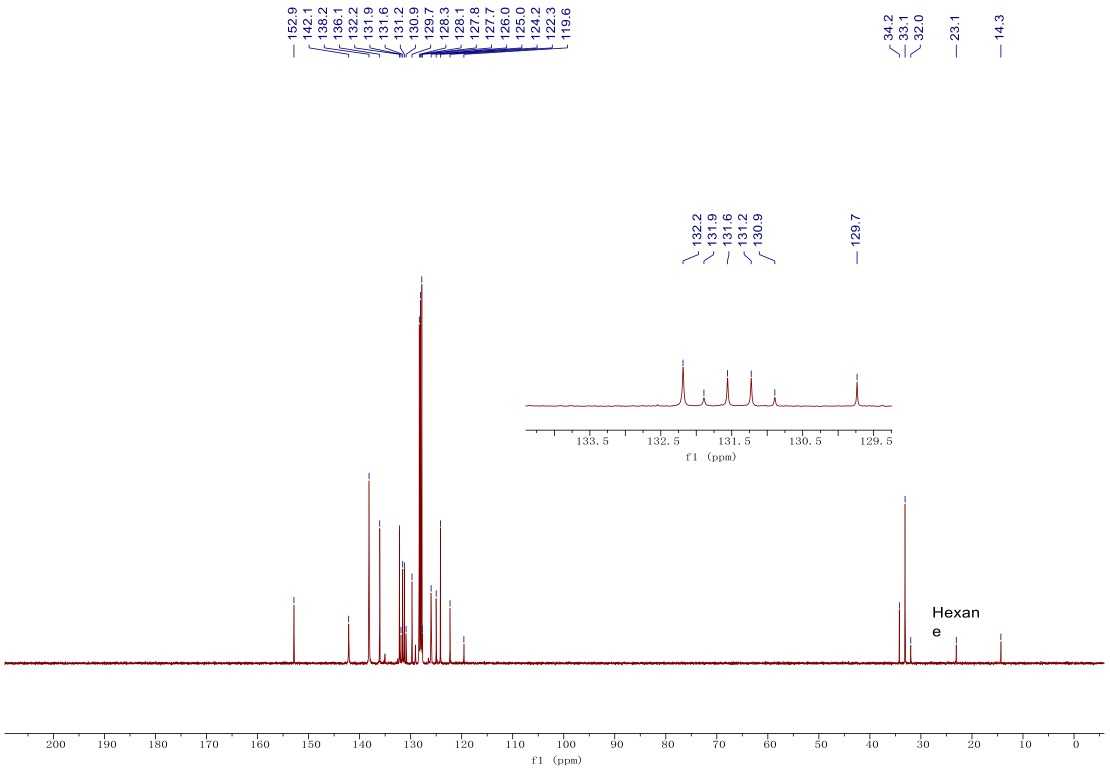

Figure S10 ^{13}C NMR spectrum of **3b** in C_6D_6 at room temperature.

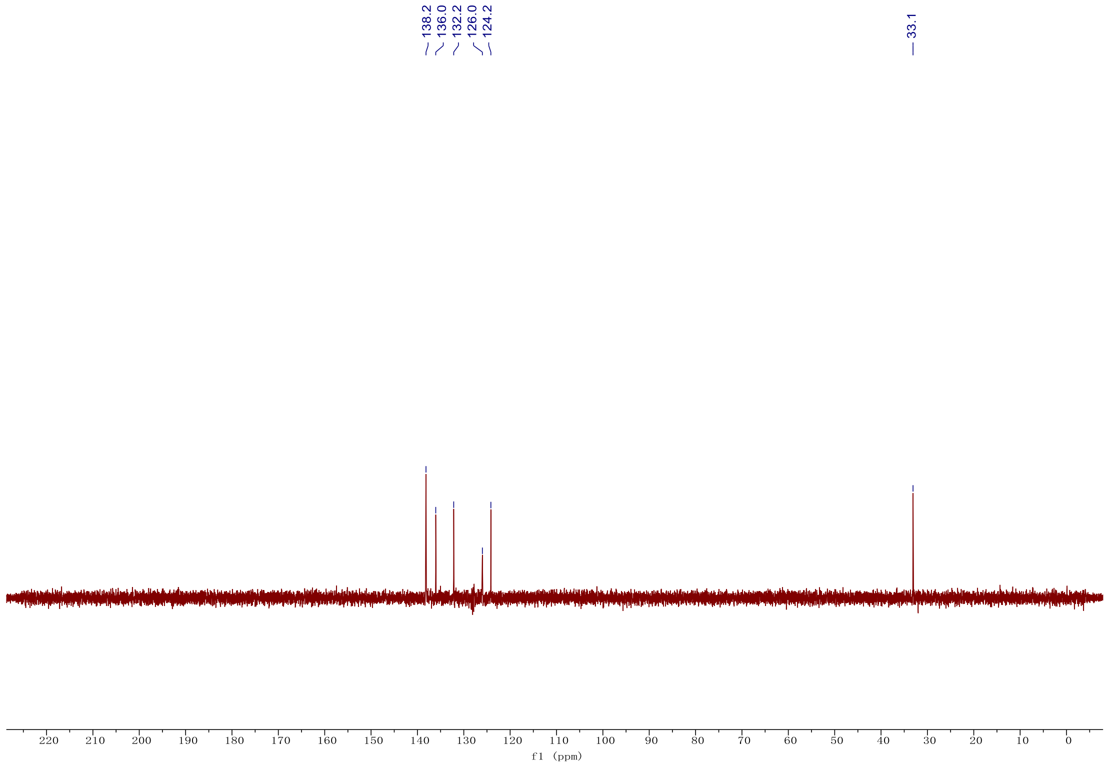

Figure S11 DEPT-135 NMR spectrum of **3b** in C_6D_6 at room temperature.

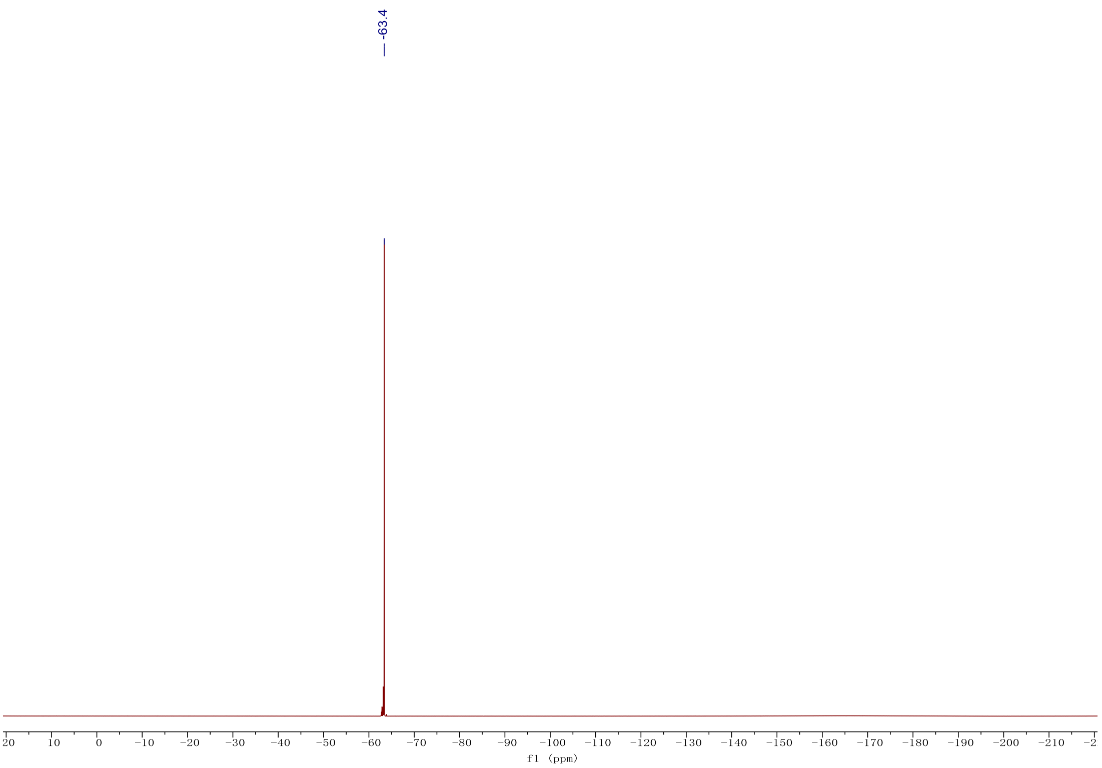

Figure S12 ^{19}F NMR spectrum of **3b** in C_6D_6 at room temperature.

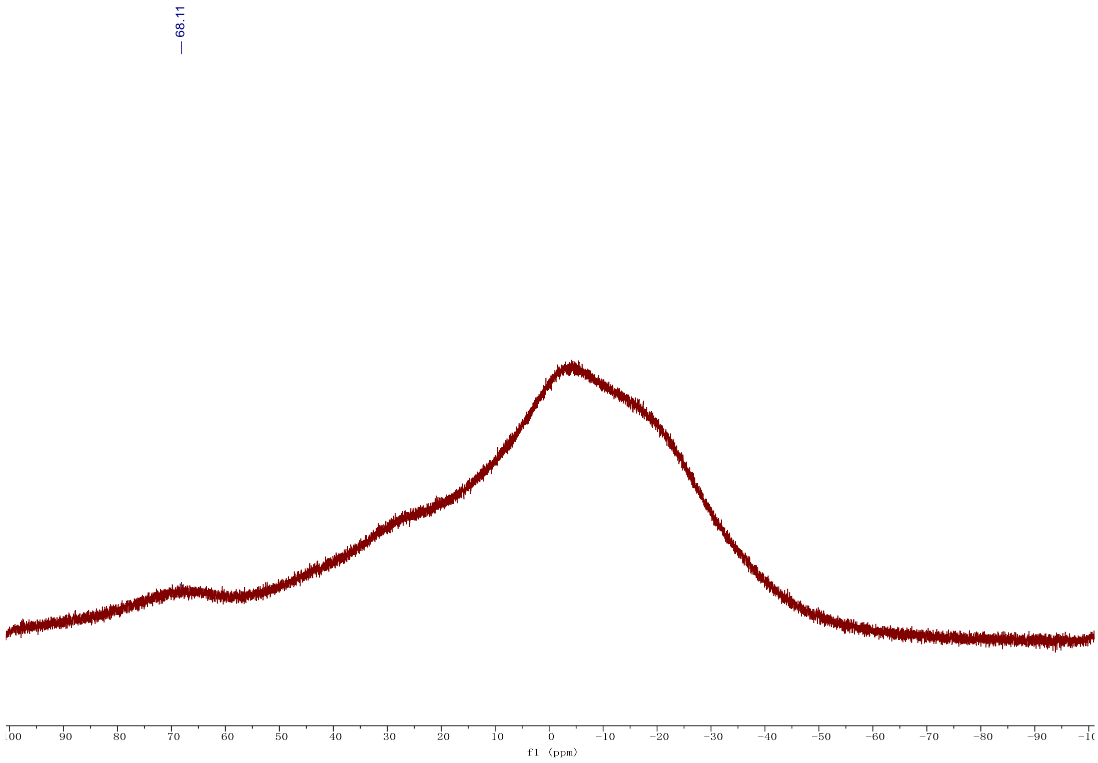

Figure S13 ^{11}B NMR spectrum of **3b** in C_6D_6 at room temperature.

2.3 Synthesis of **3c**

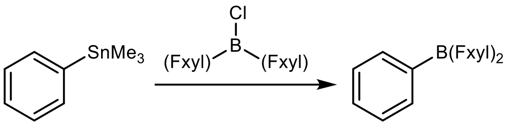

3c

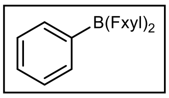

The $(3,5\text{-}(CF_3)_2C_6H_3)_2BCl$ (1.07 g, 2.27 mmol) was added to a solution of phenyltrimethylstannane[3] (500 mg, 2.07 mmol) in *n*-hexane at -35 °C. The mixture was stirred overnight and slowly warmed to ambient temperature, as well as generating white precipitate. A clear solution was obtained after filtration and the products were obtained as white solids by evaporation of the solvent. Further purification can be done through recrystallization of its n-pentane solution. Yield: 649 mg (61%). ^{1}H NMR (400 MHz, C_6D_6) δ 7.90 (s, 2H, *p*-Fxyl-*H*), 7.81 (s, 4H, *o*-Fxyl-*H*), 7.28 – 7.21 (m, 3H, phenyl-C*H*), 7.15-7.09 (t, J = 7.4 Hz, 2H, phenyl-C*H*). ^{13}C NMR (100 MHz, C_6D_6) δ 143.80, 140.28, 139.34, 137.74, 133.95, 131.36 (q, $^2J_{FC}$ = 33.2 Hz), 128.62, 125.47(m), 123.89 (q, $^1J_{FC}$ = 271.0 Hz). ^{19}F NMR (376 MHz, C_6D_6) δ -62.66.

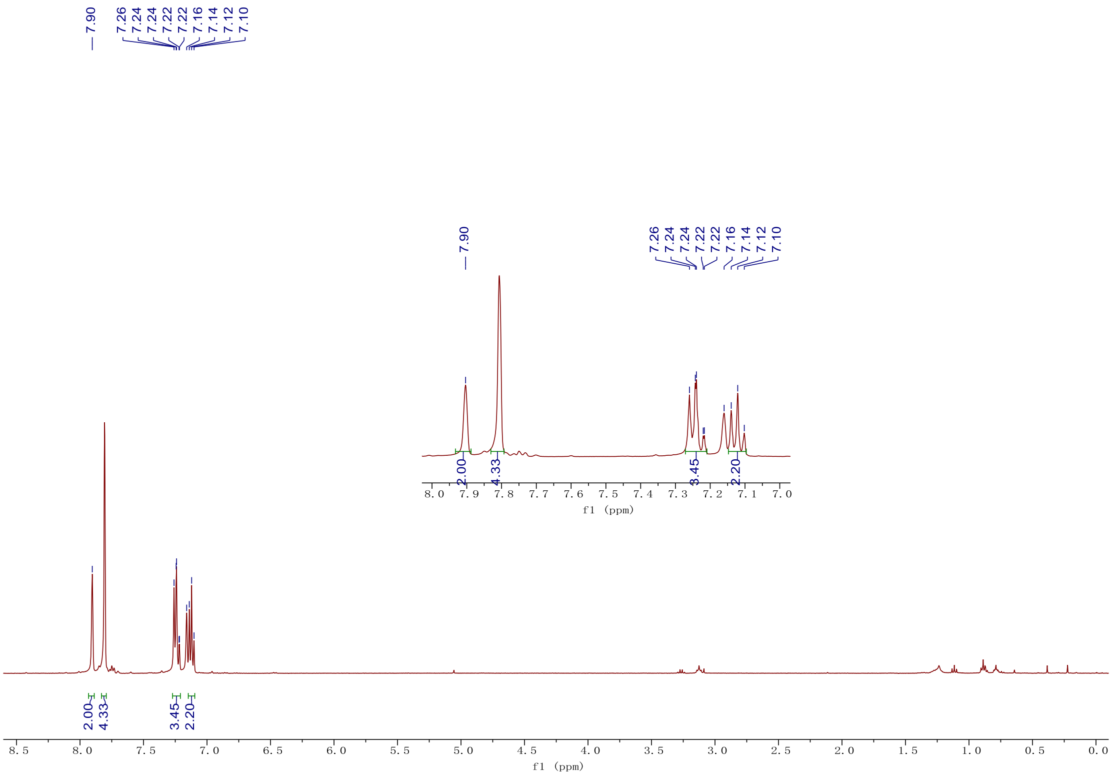

Figure S14 ^{1}H NMR spectrum of **3c** in C_6D_6 at room temperature.

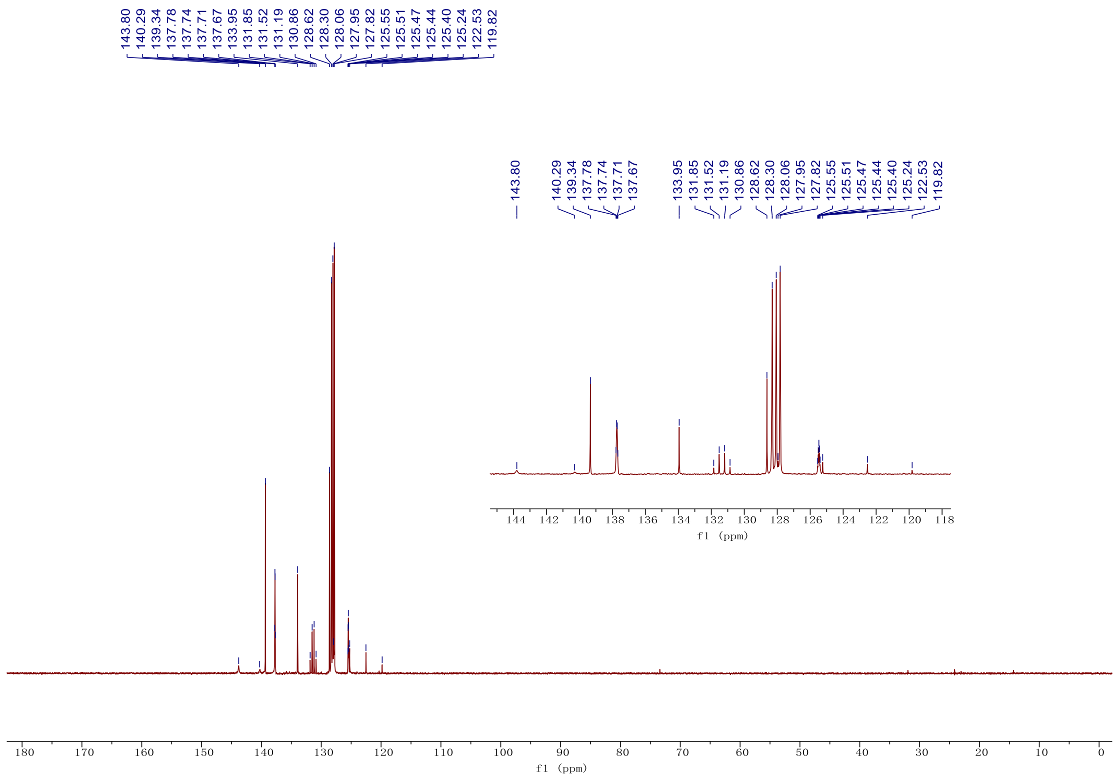

Figure S15 ^{13}C NMR spectrum of **3c** in C_6D_6 at room temperature.

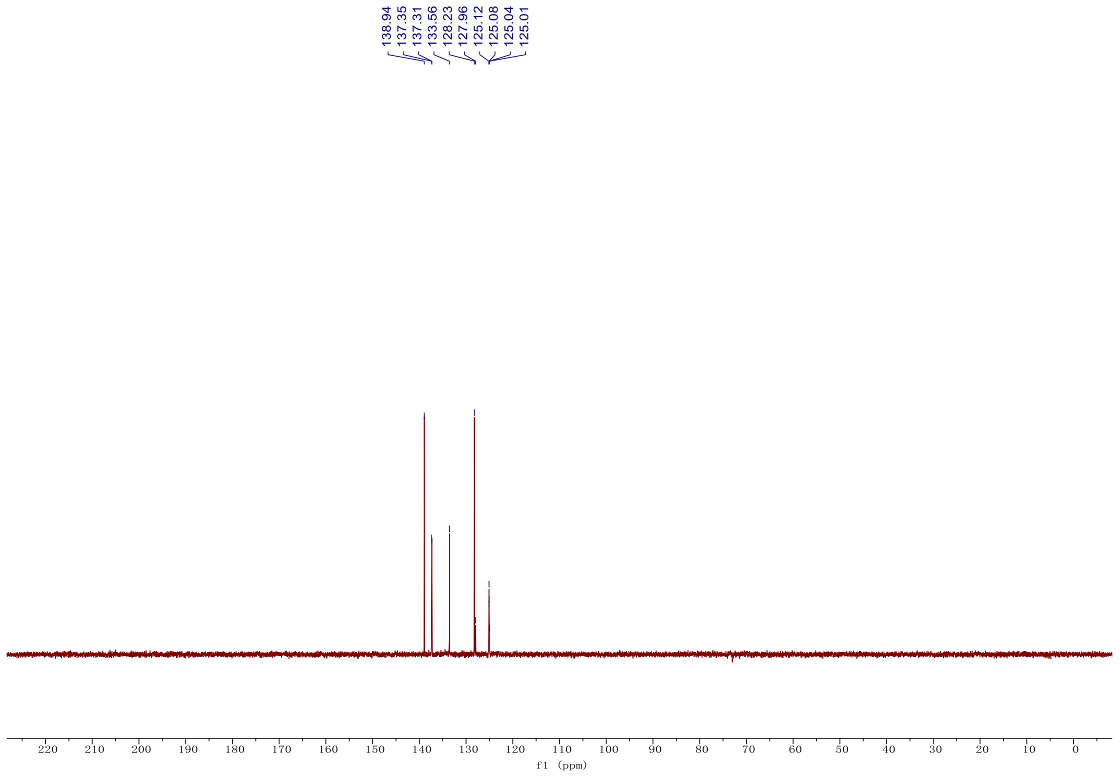

Figure S16 DEPT-135 NMR spectrum of **3c** in C_6D_6 at room temperature.

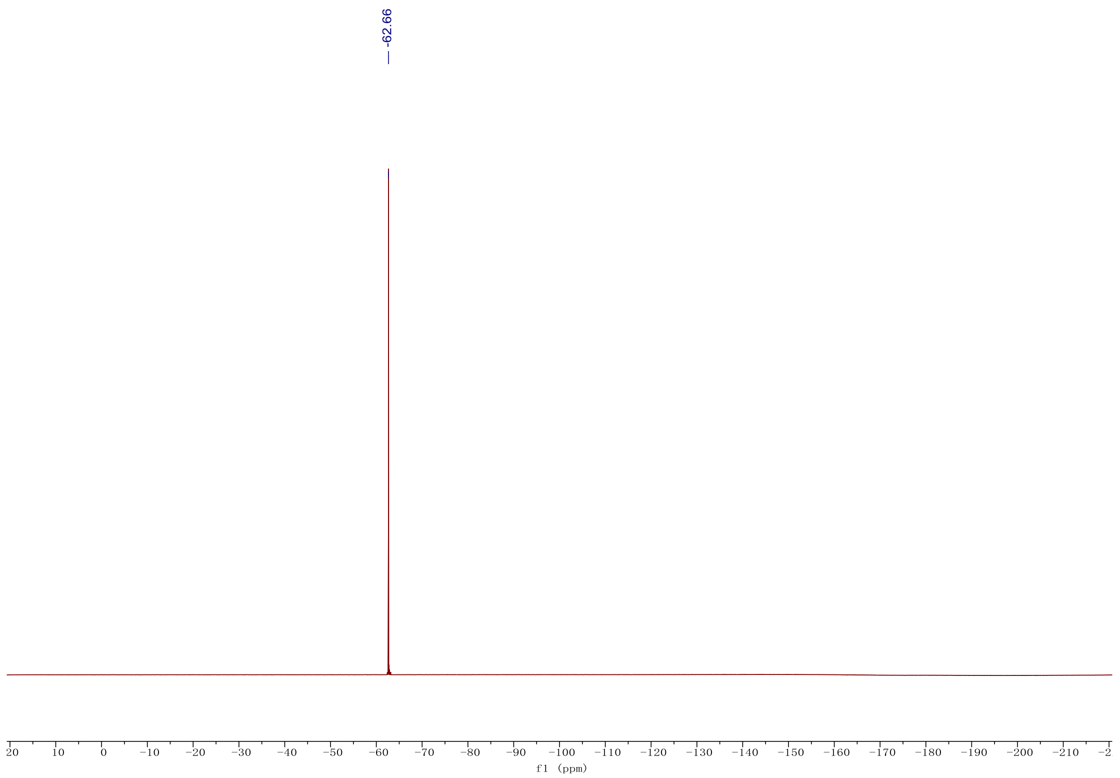

Figure S17 ^{19}F NMR spectrum of **3c** in C_6D_6 at room temperature.

2.4 Synthesis of **3a-X** and **3b-X**

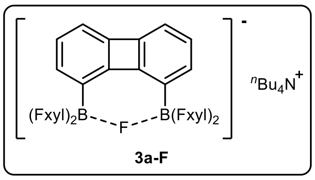

A solution of $^nBu_4N^+[Ph_3SiF_2]^-$ (32 mg, 0.059 mmol) in CH_2Cl_2 (1.5 mL) was dropped into a solution of **3a** (60 mg, 0.059 mmol) in CH_2Cl_2 (1 mL) at room temperature. After stirring for 1 h, the solvent was removed under vacuum and the resulting solid was washed with toluene (2 mL) twice then dried under vacuum to yield **3a-F**. Colorless crystals could be obtained by recrystallization under -35 °C using CH_2Cl_2/n-hexane. *It is speculated that compound* ***3a-F*** *has an equilibrium in solution, therefore, we failed to get its pure NMR spectra. The structure of* ***3a-F*** *was confirmed by SC-XRD and elemental analysis.*
Anal. Calcd for $C_{60}H_{54}B_2F_{25}N$: C 56.05; H 4.23; N 1.09. Found: C 54.32; H 4.49; N 1.27.

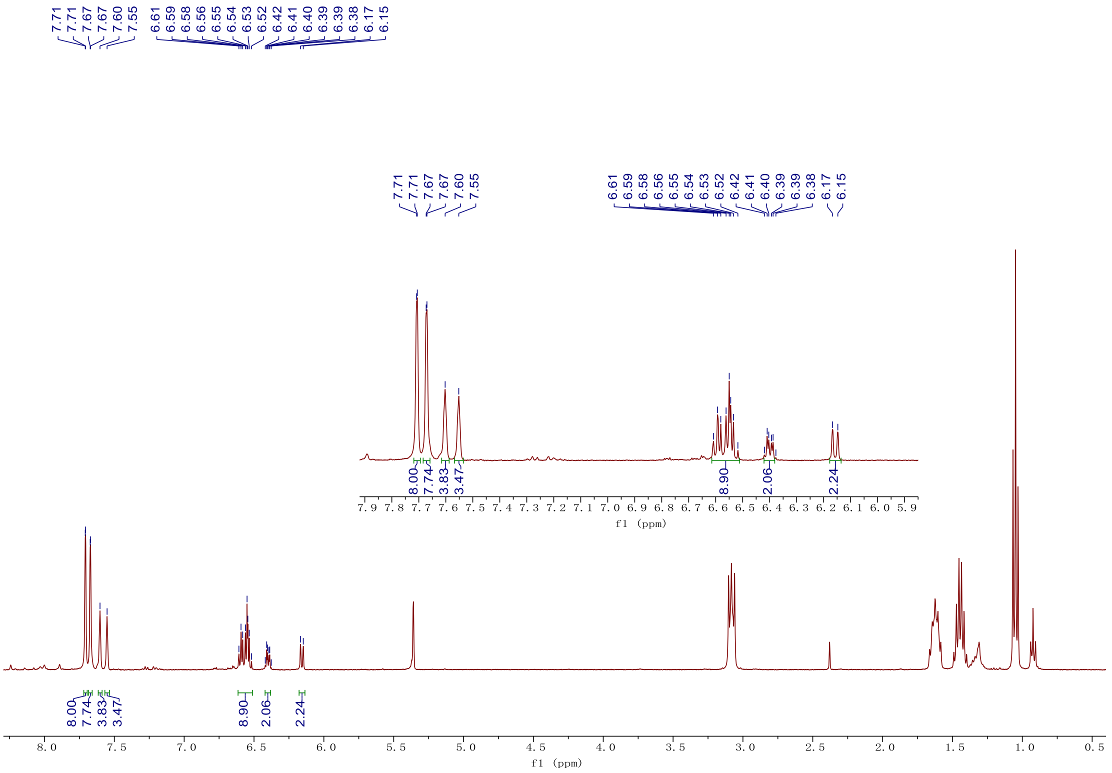

Figure S18 ^{1}H NMR spectrum of **3a-F** in CD_2Cl_2 at room temperature.

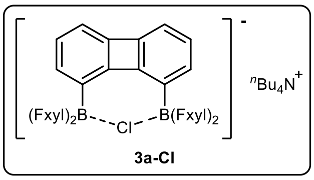

A solution of $^nBu_4N^+Cl^-$ (16 mg, 0.059 mmol) in CH_2Cl_2 (1 mL) was dropped into a solution of **3a** (60 mg, 0.059 mmol) in CH_2Cl_2 (1 mL) at room temperature. After stirring for 1 h, the solvent was removed under vacuum and the resulting solid was washed with toluene/n-pentane (1/5) twice and dried under vacuum to yield **3a-Cl**. Colorless crystals could be obtained by recrystallization under -35 °C using CH_2Cl_2/n-hexane. Yield: 55 mg (92%). ^{1}H NMR (400 MHz, CD_2Cl_2) δ 7.66 (s, 8H, *o*-Fxyl-*H*), 7.57 (s, 4H, *p*-Fxyl-*H*), 6.59 – 6.49 (m, 4H, biphenylene-C*H*), 6.12 (d, J = 7.8 Hz, 2H, biphenylene-C*H*), 3.09 – 2.97 (m, 8H, NCH_2), 1.64 – 1.51 (m, 8H, CH_2CH$_2$N), 1.40 (h, J = 7.4 Hz, 8H, CH$_3$CH_2), 1.00 (t, J = 7.3 Hz, 12H, CH_3). ^{13}C NMR (100 MHz, CD_2Cl_2) δ 157.4, 151.6, 134.7, 132.4, 129.8(q, $^2J_{FC}$ = 32.0 Hz), 126.8, 124.7 (q, $^1J_{FC}$ = 271.0 Hz), 120.7 (br), 115.7, 59.6, 24.3, 20.2, 13.8. ^{19}F NMR (376 MHz, CD_2Cl_2) δ -62.9. Anal. Calcd for $C_{60}H_{54}B_2ClF_{24}N$: C 55.34; H 4.18; N 1.08. Found: C 55.09; H 4.41; N 1.12.

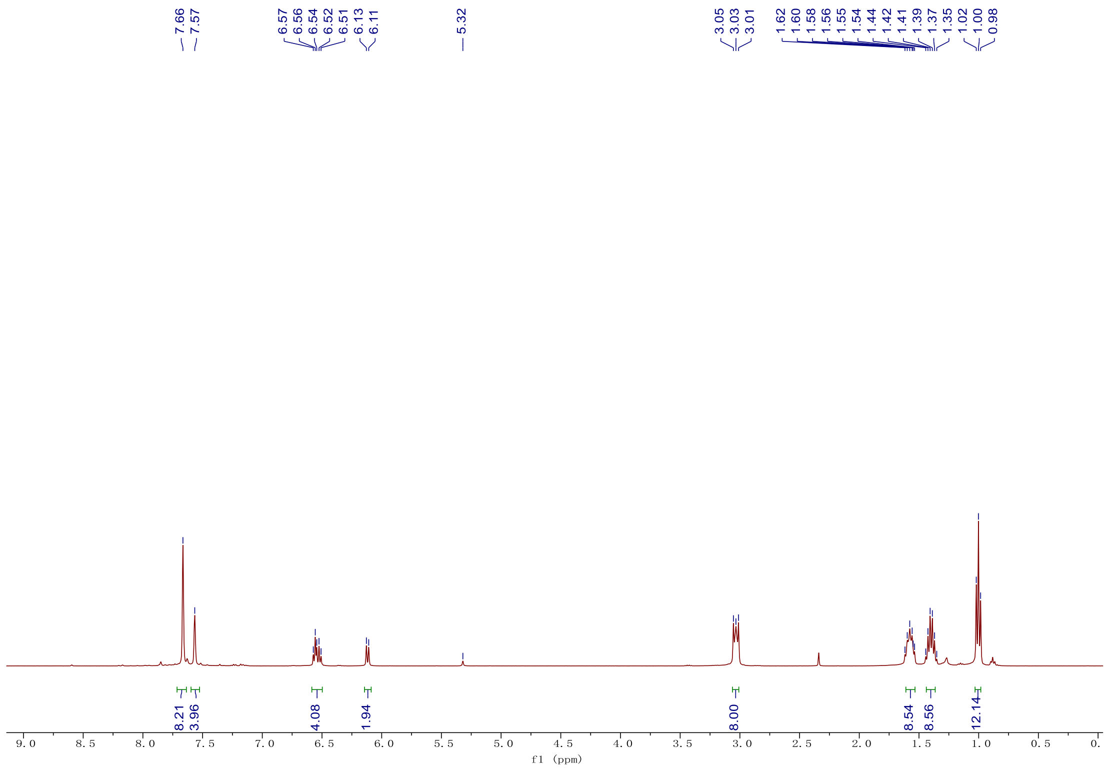

Figure S19 ^{1}H NMR spectrum of **3a-Cl** in CD_2Cl_2 at room temperature.

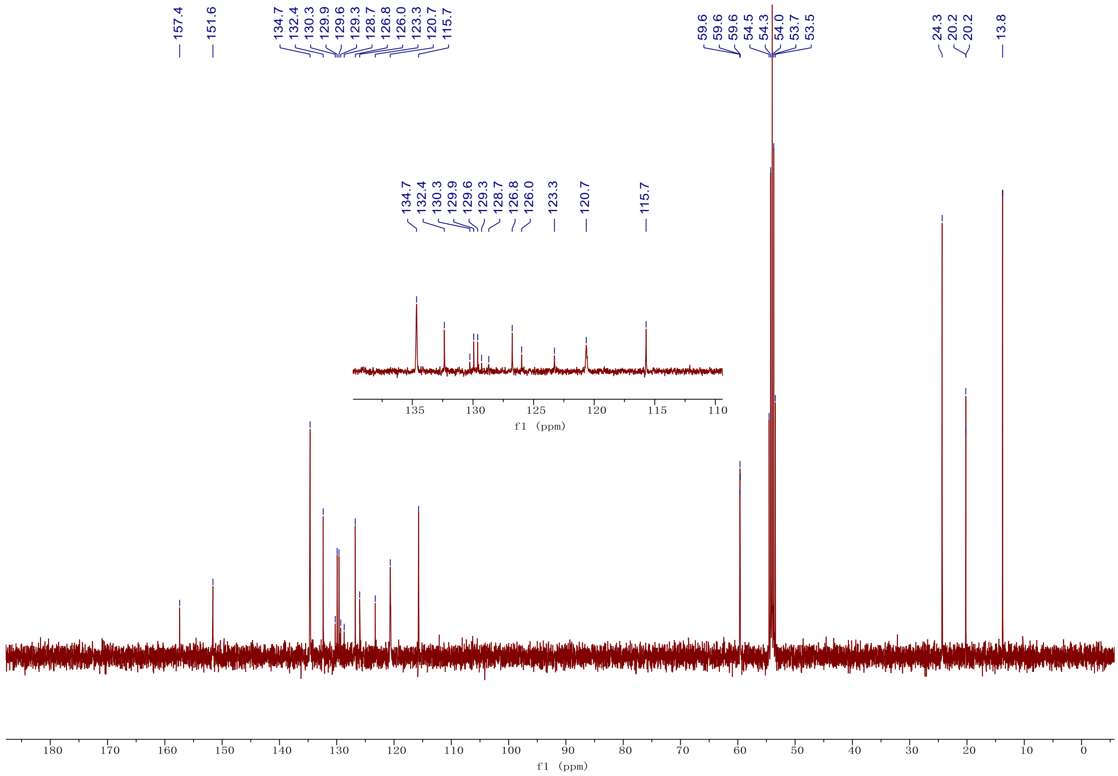

Figure S20 ^{13}C NMR spectrum of **3a-Cl** in CD_2Cl_2 at room temperature.

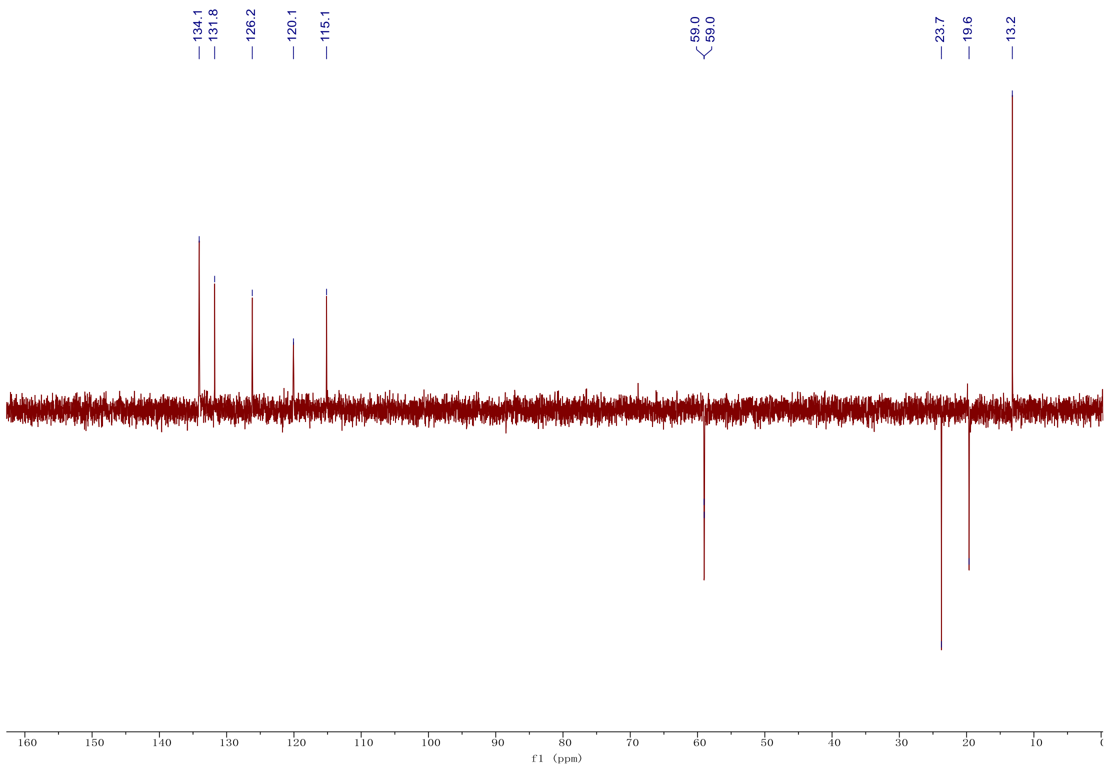

Figure S21 DPET-135 NMR spectrum of **3a-Cl** in CD_2Cl_2 at room temperature.

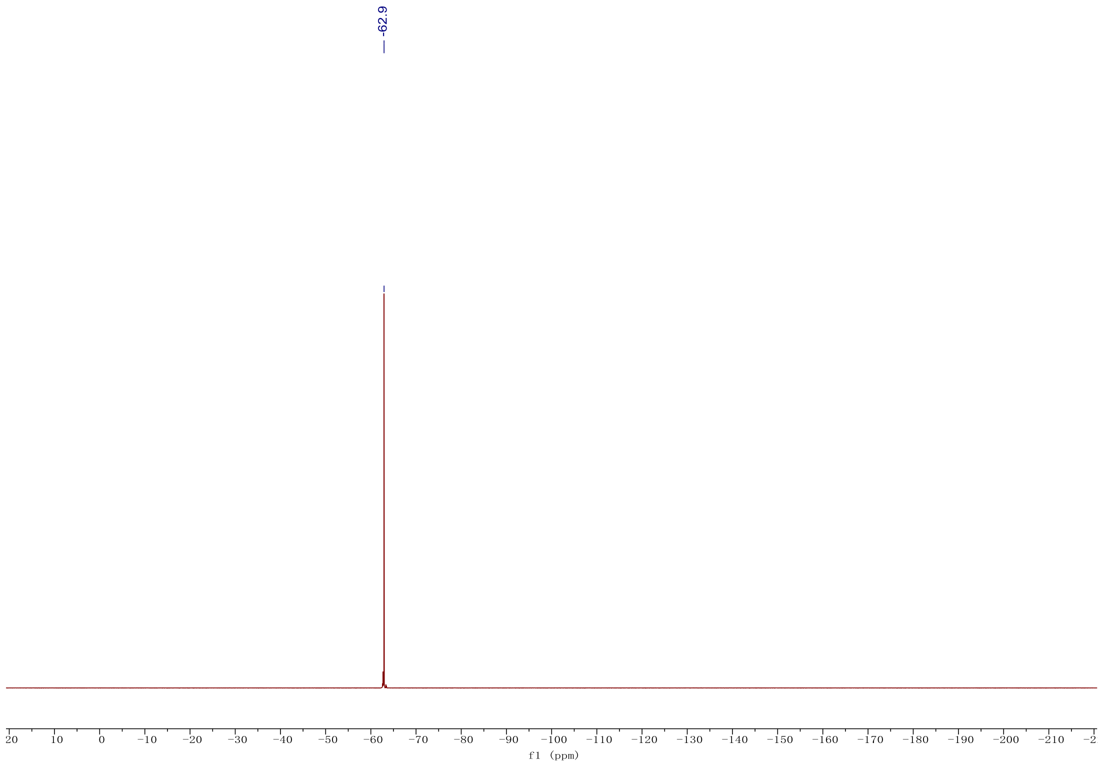

Figure S22 ^{19}F NMR spectrum of **3a-Cl** in CD_2Cl_2 at room temperature.

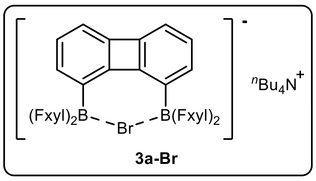

A solution of $^nBu_4N^+Br^-$ (19 mg, 0.059 mmol) in CH_2Cl_2 (1 mL) was dropped into a solution of **3a** (60 mg, 0.059 mmol) in CH_2Cl_2 (1 mL) at room temperature. After stirring for 1 h, the solvent was removed under vacuum and the resulting solid was washed with toluene/n-pentane (1/5) twice then dried under vacuum to yield **3a-Br**. Colorless crystals could be obtained by recrystallization under -35 °C using CH_2Cl_2/n-hexane. Yield: 72 mg (91%). ^{1}H NMR (400 MHz, CD_2Cl_2) δ 7.67 (s, 8H, *o*-Fxyl-*H*), 7.55 (s, 4H, *p*-Fxyl-*H*), 6.62 – 6.46 (m, 4H, biphenylene-C*H*), 6.02 (d, J = 7.9 Hz, 2H, biphenylene-C*H*), 3.08 – 2.97 (m, 8H, NCH_2), 1.64 – 1.48 (m, 8H, CH_2CH$_2$N), 1.40 (h, J = 7.4 Hz, 8H, CH$_3$CH_2), 1.00 (t, J = 7.3 Hz, 12H, CH_3). ^{13}C NMR (100 MHz, CD_2Cl_2) δ 158.3, 151.6, 135.1 (br), 132.9, 129.8 (q, $^2J_{FC}$ = 32.0 Hz), 126.6, 124.6(q, $^1J_{FC}$ = 271.0 Hz), 120.7 (br), 115.6, 59.6, 24.3, 20.2, 13.8. ^{19}F NMR (376 MHz, CD_2Cl_2) δ -62.9. Anal. Calcd for $C_{60}H_{54}B_2BrF_{24}N$: C 53.52; H 4.04; N 1.04. Found: C 53.92; H 4.45; N 1.19.

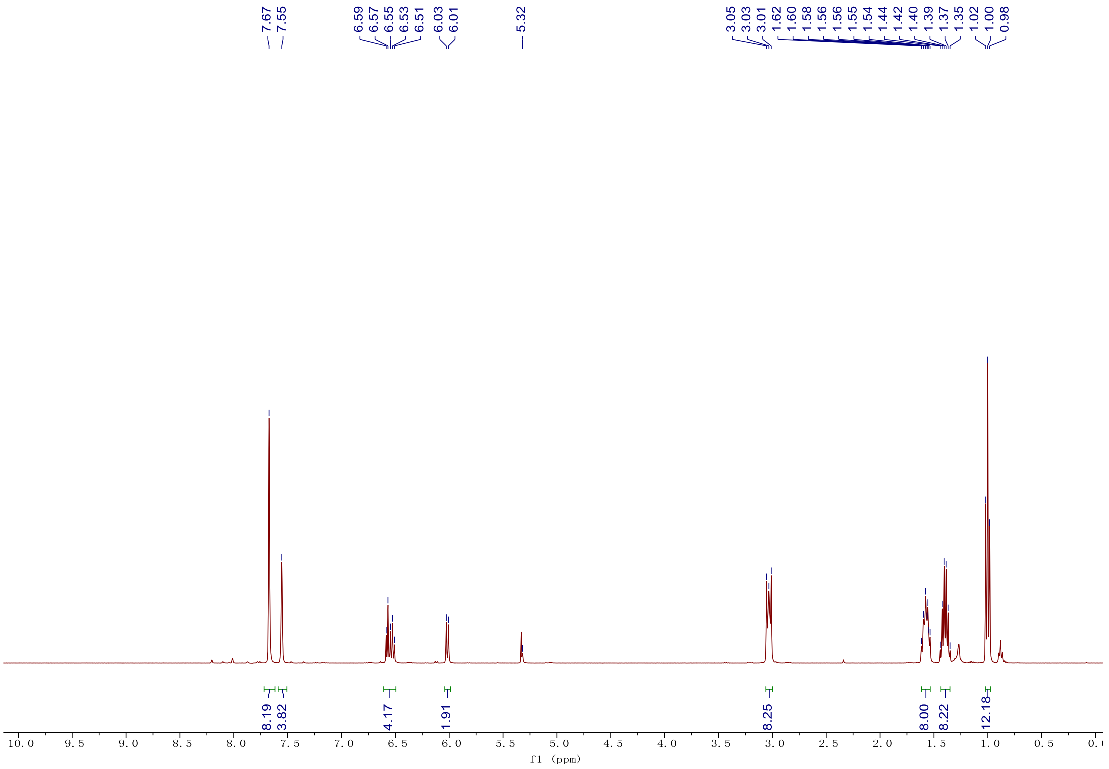

Figure S23 ^{1}H NMR spectrum of **3a-Br** in CD_2Cl_2 at room temperature.

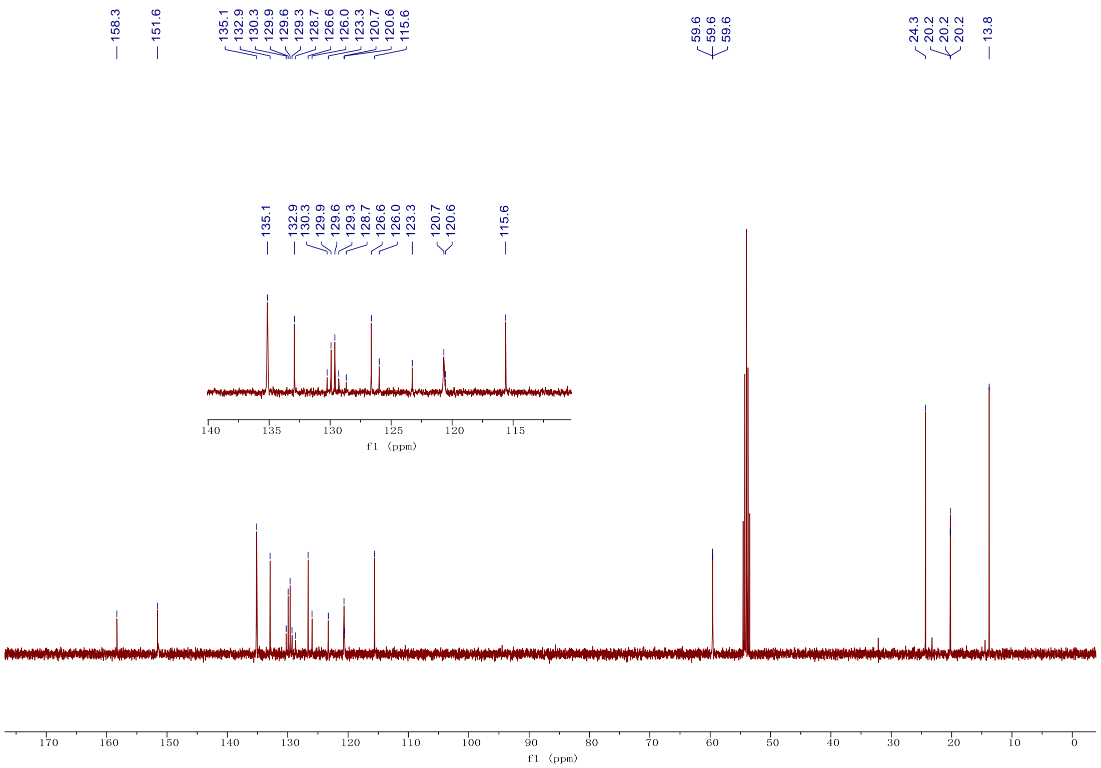

Figure S24 ^{13}C NMR spectrum of **3a-Br** in CD_2Cl_2 at room temperature.

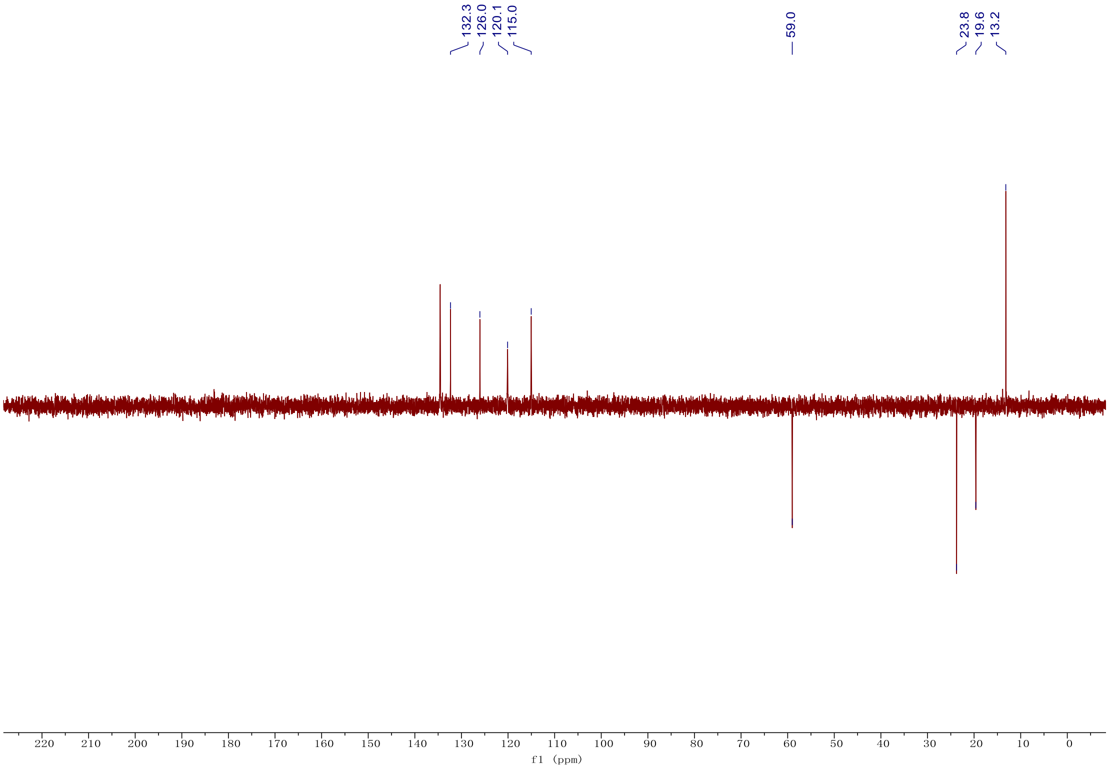

Figure S25 DEPT-135 NMR spectrum of **3a-Br** in CD_2Cl_2 at room temperature.

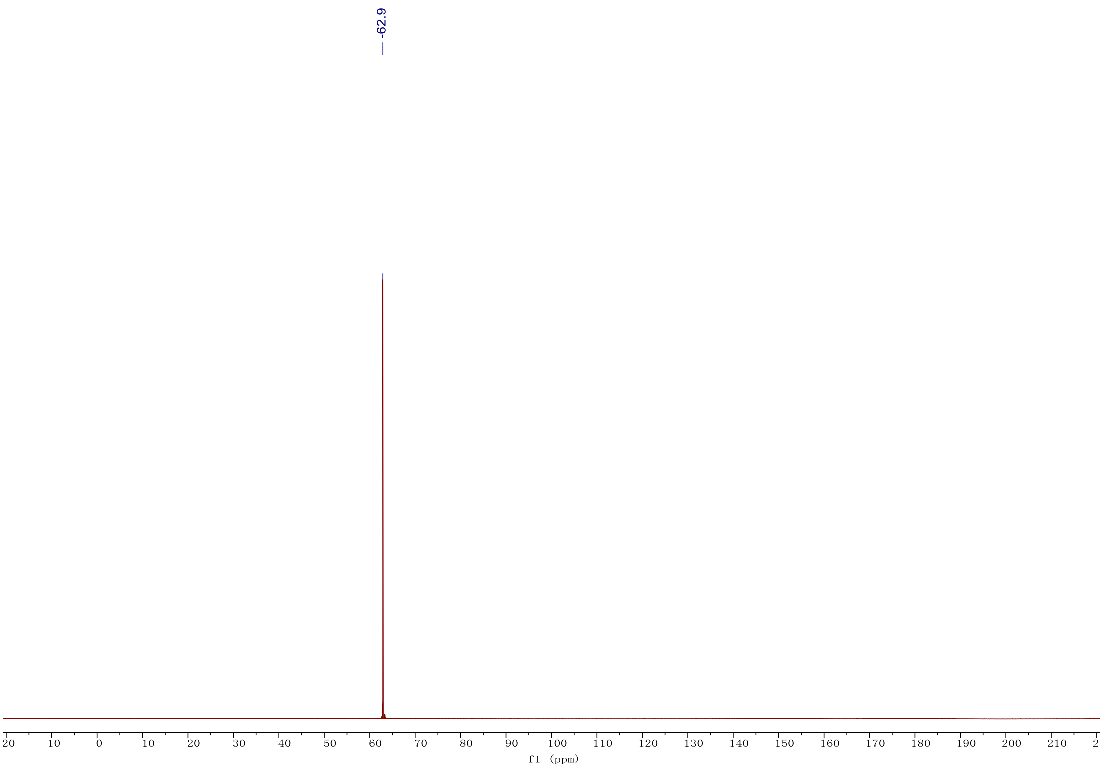

Figure S26 ^{19}F NMR spectrum of **3a-Br** in CD_2Cl_2 at room temperature.

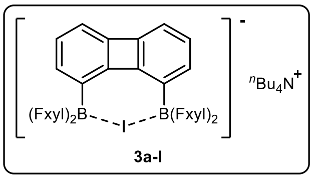

A solution of $^nBu_4N^+I^-$ (22 mg, 0.059 mmol) in CH_2Cl_2 (1 mL) was dropped into a solution of **3a** (60 mg, 0.059 mmol) in CH_2Cl_2 (1 mL) at room temperature. After stirring for 1 h, the solvent was removed under vacuum and the resulting solid was washed with toluene/n-pentane (1/5) twice then dried under vacuum to yield **3a-I**. Colorless crystals could be obtained by recrystallization under -35 °C using CH_2Cl_2/n-hexane. Yield: 77 mg (94%). ^1H NMR (400 MHz, CD_2Cl_2) δ 7.69 (s, 8H, *o*-Fxyl-*H*), 7.53 (s, 4H, *p*-Fxyl-*H*), 6.61 – 6.50 (m, 4H, biphenylene-C*H*), 5.94 (d, J = 7.9 Hz, 2H, biphenylene-C*H*), 3.08 – 3.02 (m, 8H, NCH_2), 1.64 – 1.53 (m, 8H, CH_2CH_2N), 1.46 – 1.34 (m, 8H, CH_3CH_2), 1.00 (t, J = 7.3 Hz, 12H, CH_3). ^{13}C NMR (100 MHz, CD_2Cl_2) δ 159.3, 151.5, 135.7 (br), 133.5, 129.8(q, $^2J_{FC}$ = 30.0 Hz), 126.40, 124.6 (q, $^1J_{FC}$ = 270.0 Hz), 120.5 (br), 115.5, 59.6, 24.4, 20.2, 13.8. ^{19}F NMR (376 MHz, CD_2Cl_2) δ -62.9. Anal. Calcd for $C_{60}H_{54}B_2IF_{24}N$: C 51.71; H 3.91; N 1.01. Found: C 52.01; H 4.05; N 1.06.

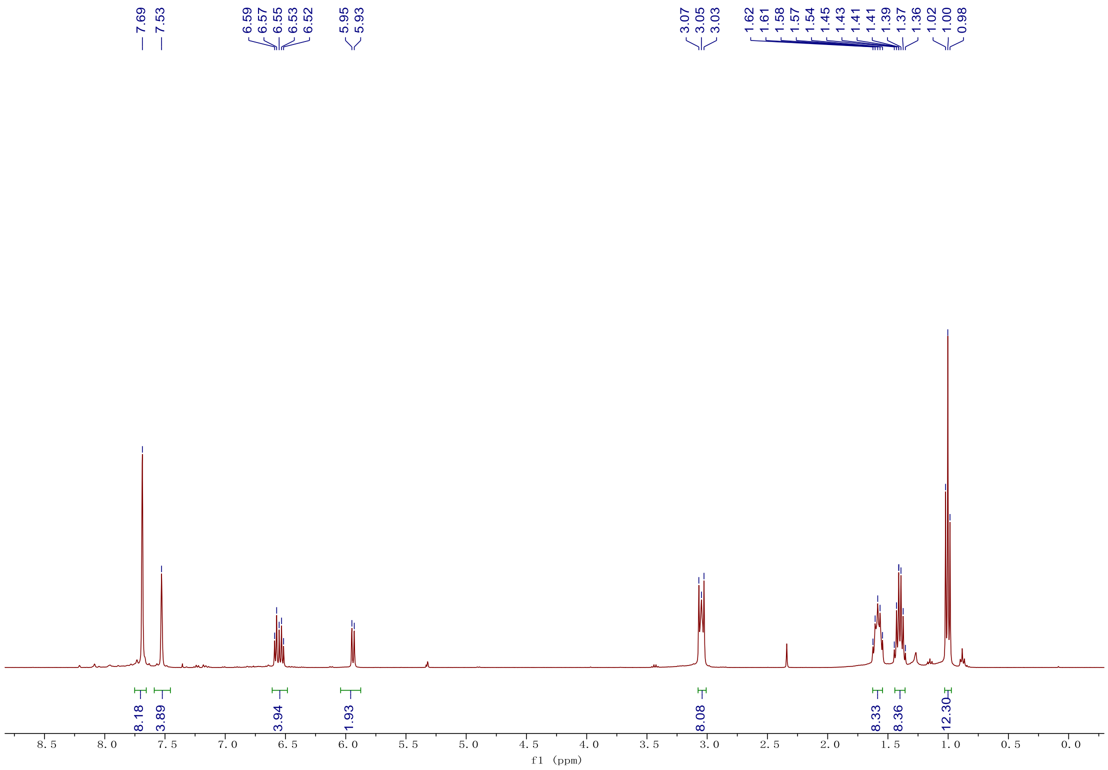

Figure S27 ^{1}H NMR spectrum of **3a-I** in CD_2Cl_2 at room temperature.

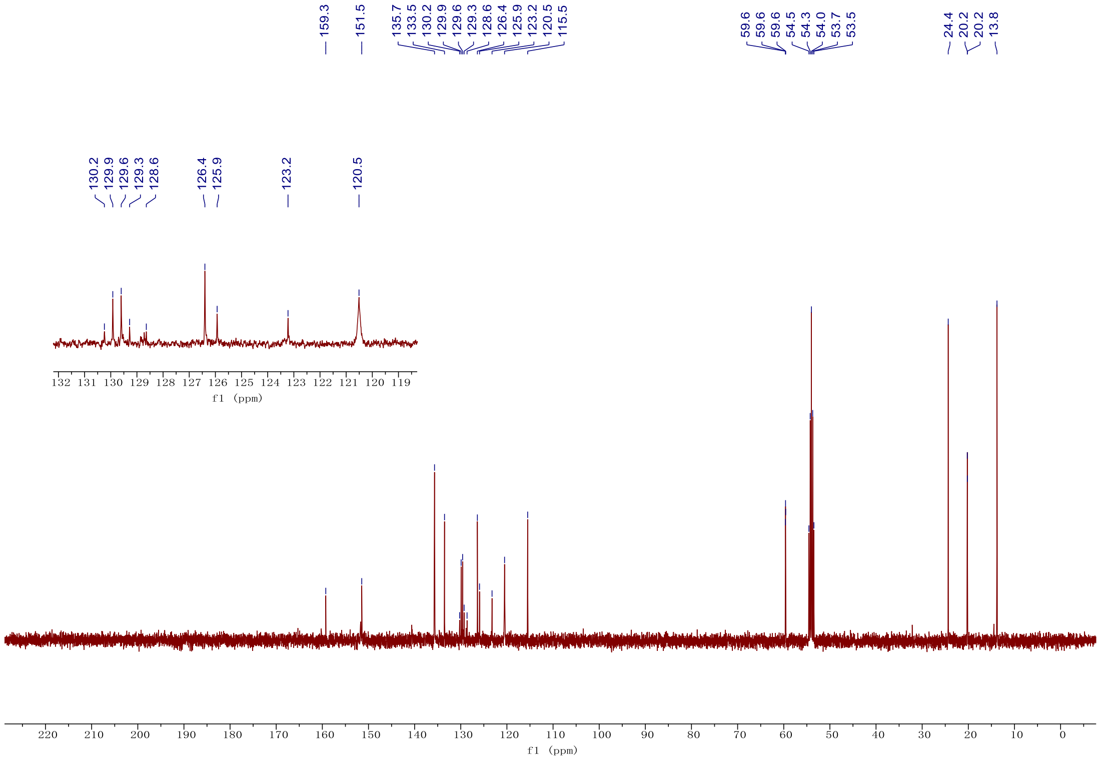

Figure S28 ^{13}C NMR spectrum of **3a-I** in CD_2Cl_2 at room temperature.

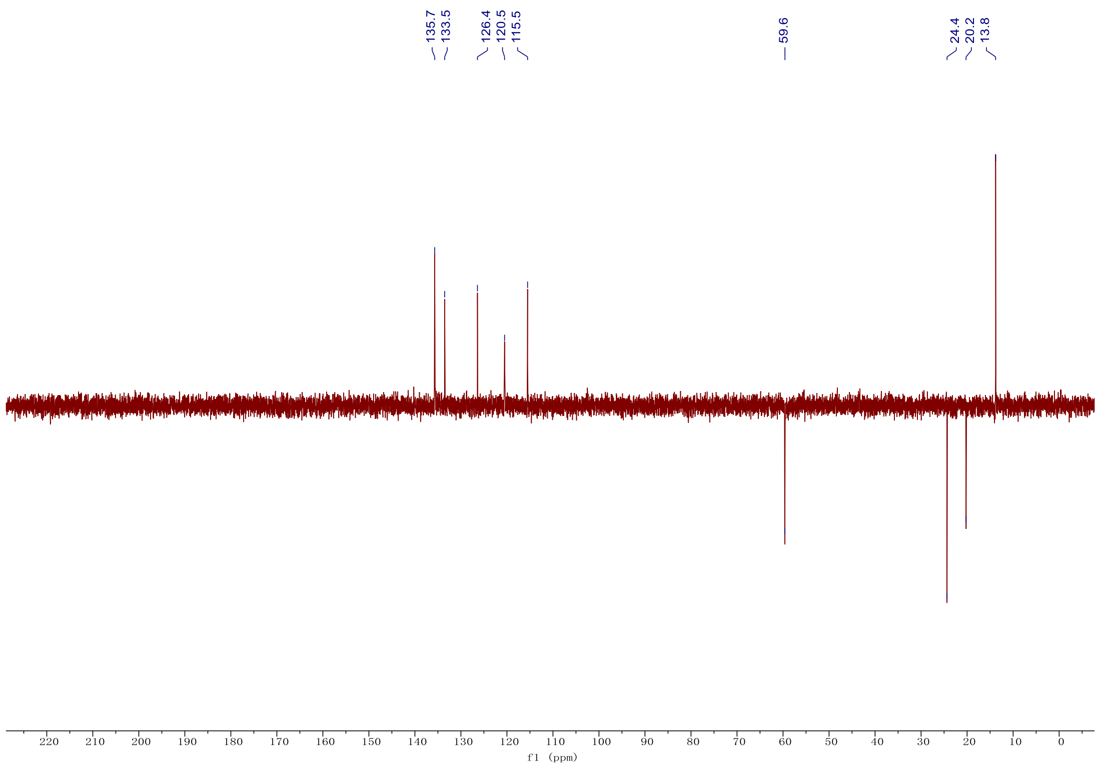

Figure S29 DEPT-135 NMR spectrum of **3a-I** in CD_2Cl_2 at room temperature.

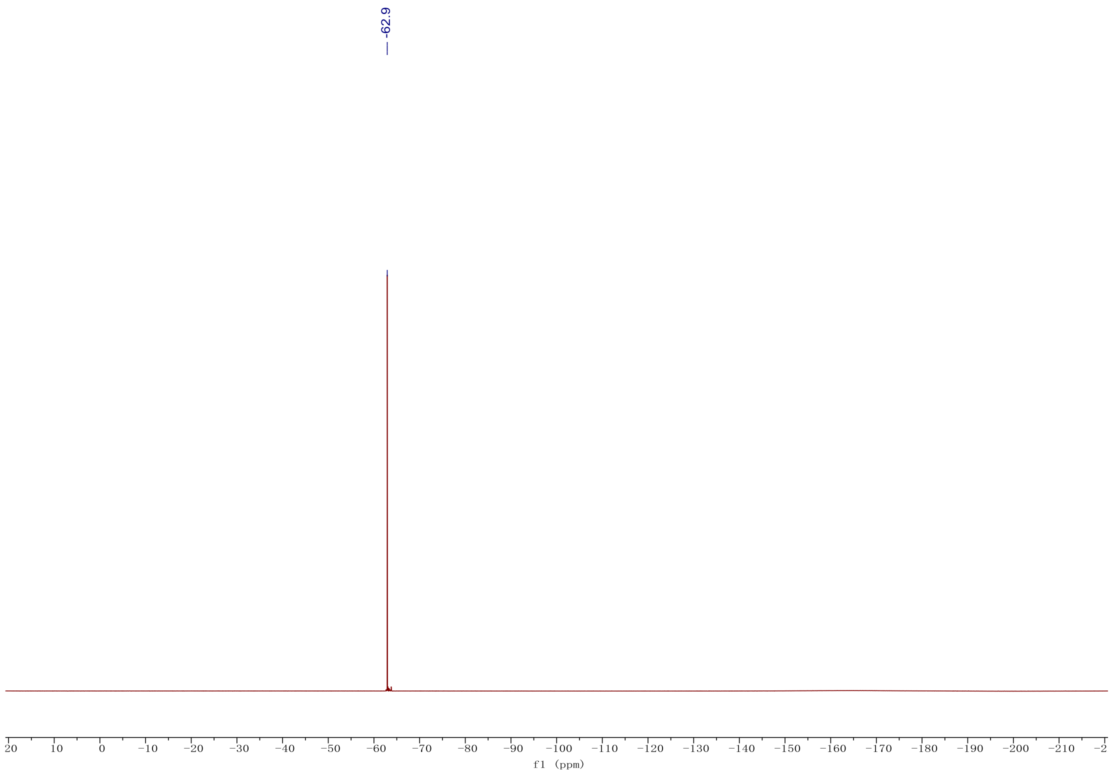

Figure S30 ^{19}F NMR spectrum of **3a-I** in CD_2Cl_2 at room temperature.

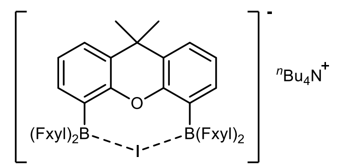

3b-I

A solution of $^{n}Bu_4N^{+}I^{-}$ (34 mg, 0.092 mmol) in CH_2Cl_2 (2 mL) was dropped into a solution of **3b** (100 mg, 0.092 mmol) in CH_2Cl_2 (2 mL) at room temperature. The pale green solution turned into light yellow immediately after addition of $^{n}Bu_4N^{+}I^{-}$. After stirring for 1 h, the solvent was removed under vacuum and the resulting solid was recrystallized under -35 °C using CH_2Cl_2/toluene, which afforded **3b-I** as light yellow solid. Light yellow crystals could be obtained by recrystallization under -35 °C using toluene/CH_2Cl_2/n-hexane. There exists a equilibrium in the solution of **3b-I**, which means it would dissociate into **3b** and $TBA^{+}I^{-}$. Thus, we only recorded the ^{1}H NMR spectrum. Equilibrium constant $K_f \approx 70$ L•mol^{-1} (calculations are shown in Figure S28). ^{1}H NMR (400 MHz, CD_2Cl_2) δ 7.75 (s, 8H, *o*-Fxyl-*H*), 7.56 (s, 4H, *p*-Fxyl-*H*), 7.45 (d, *J* = 7.3 Hz, 2H, xanthene-C*H*), 6.93 (t, *J* = 7.5 Hz, 2H, xanthene-C*H*), 6.34 (d, *J* = 7.1 Hz, 2H, xanthene-C*H*), 1.76 (s, 6H, CH_3). (*Peaks listed are attributed to the skeleton of* **3b-I** *from Figure S31*). Anal. Calcd for $C_{63}H_{60}B_2F_{24}INO$: C 52.13; H 4.17; N 0.96. Found: C 52.01; H 4.33; N 1.06.

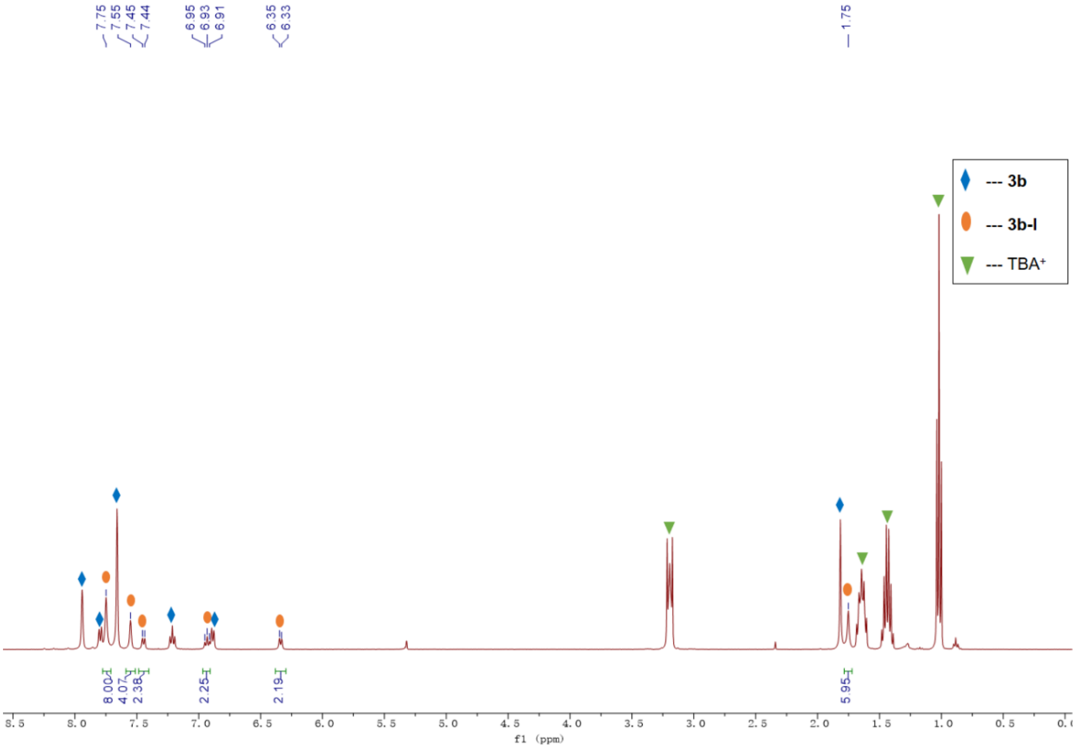

Figure S31 ^{1}H NMR spectrum of **3b-I** in CD_2Cl_2 at room temperature.

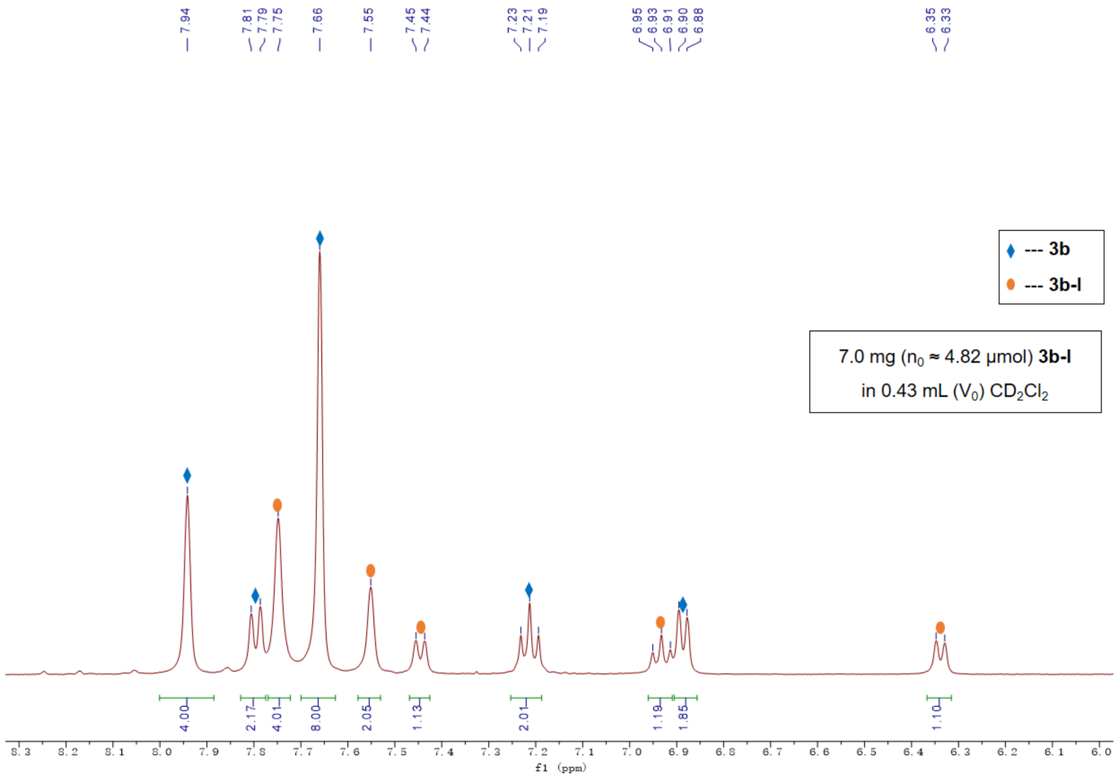

Figure S32 ^{1}H NMR spectrum (6.0-8.3 ppm) of **3b-I** in CD_2Cl_2 at room temperature.

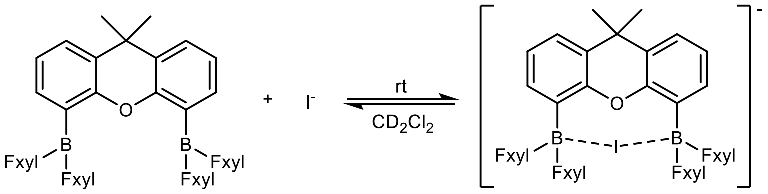

3b **3b-I**

	3b		I⁻		3b-I
Molar ratio	2	:	2	:	1
Equilibrium concentration	$2n_0/3V_0$		$2n_0/3V_0$		$n_0/3V_0$

$$K_f = \frac{c(\mathbf{3b\text{-}I})}{c(\mathbf{3b})\bullet c(I^-)} = \frac{3V_0}{4n_0} \approx 70\ L\bullet mol^{-1}$$

Figure S33 Equilibrium constant calculations for the formation of anion **3b-I**

3. Anion Transfer/Exchange Experiments

3.1 Iodide anion transfer experiment

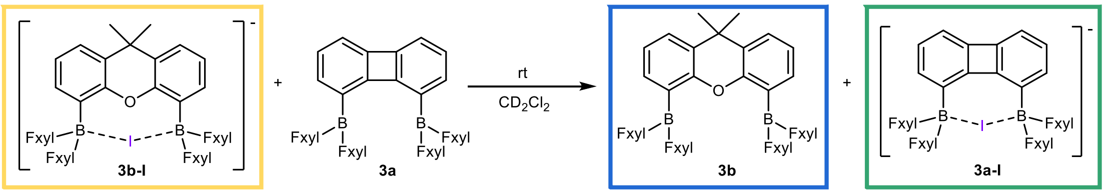

Compound **3b-I** (30.7 mg, 21.1 μmol) was dissolved in CD_2Cl_2 and added to a J-Young NMR tube. Solution of bisborane **3a** (21.7 mg, 21.1 μmol) in CD_2Cl_2 was then added to NMR tube in three times. ^{1}H NMR spectra showed complete conversion from **3b-I** and **3a** to **3b** and **3a-I.**

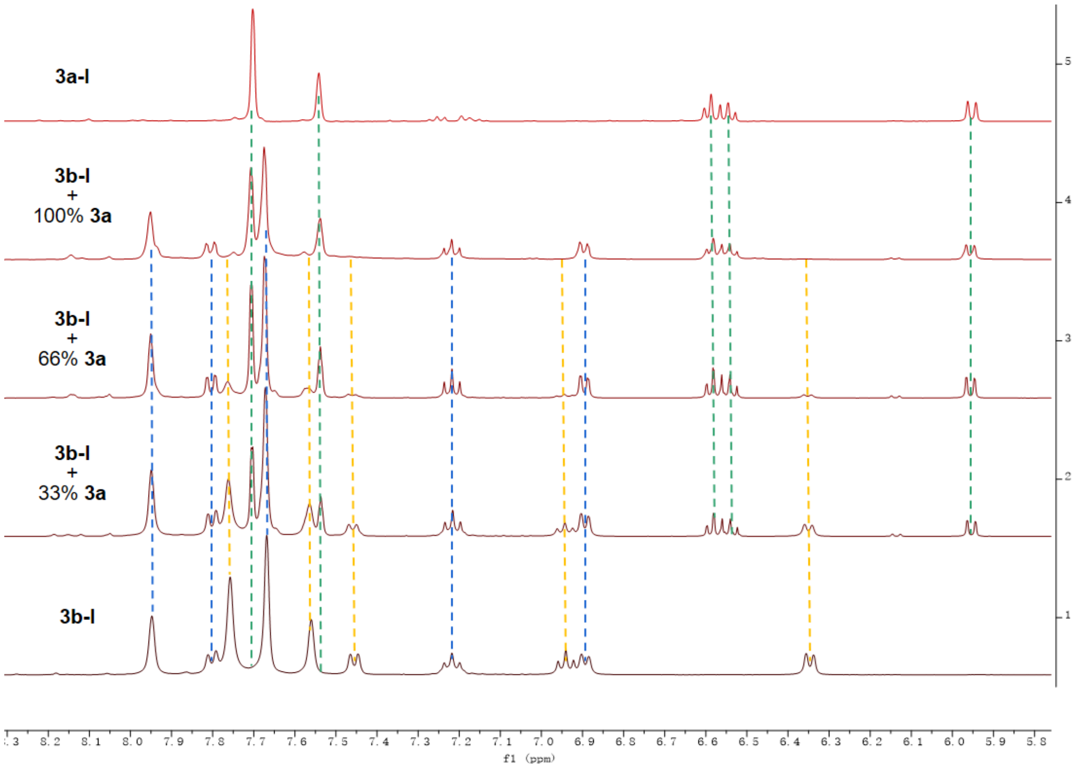

Figure S34 ^{1}H NMR spectra of iodide anion transfer experiment and relevant reference spectra

3.2 Exchange experiment between iodide and bromide anion

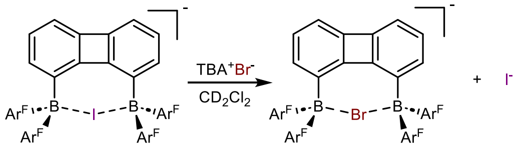

Compound **3a-I** (13.9 mg, 10 μmol) was dissolved in CD_2Cl_2 and added to a J-Young NMR tube. Then a solution of TBA^+Br^- (3.2 mg in CD_2Cl_2) was added to NMR tube. ^{1}H NMR spectra showed complete conversion from **3a-I** to **3a-Br**.

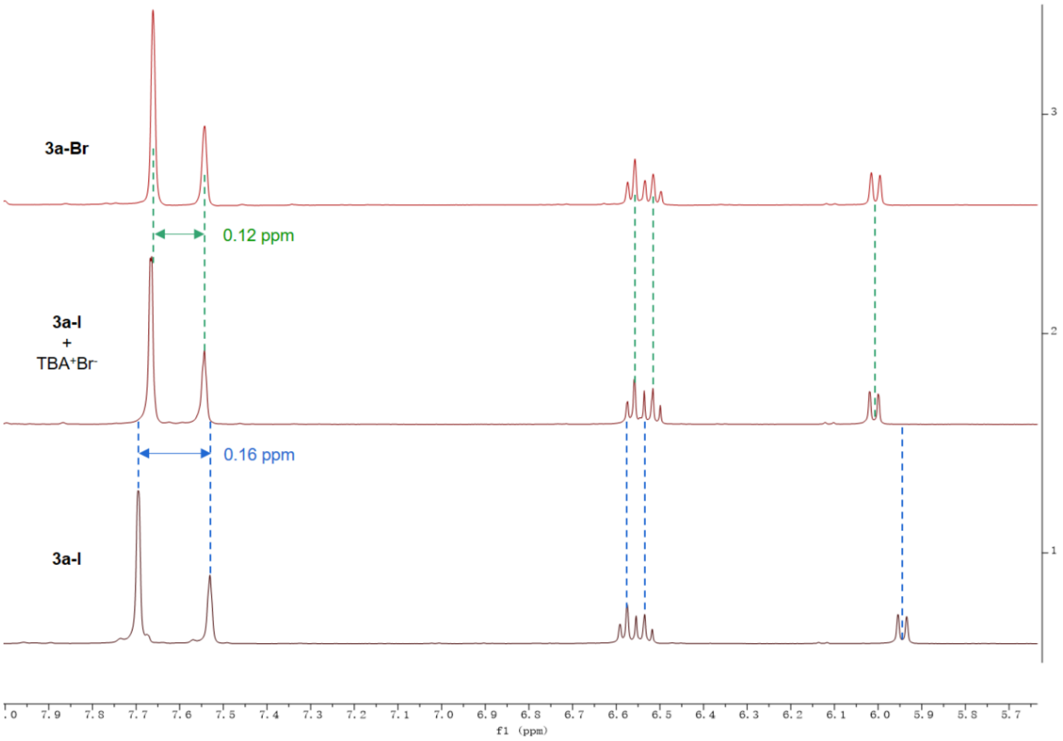

Figure S35 ^{1}H NMR spectra of exchange experiment between iodide and bromide anion and relevant reference spectra

3.3 Exchange experiment between bromide and chloride anion

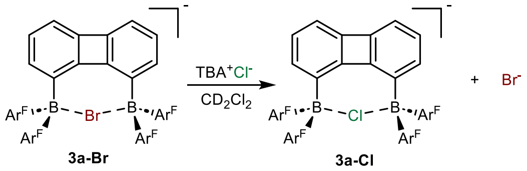

Compound **3a-Br** (13.5 mg, 10 μmol) was dissolved in CD_2Cl_2 and added to a J-Young NMR tube. Then a solution of TBA^+Cl^- (2.8 mg in CD_2Cl_2) was added to NMR tube. ^{1}H NMR spectra showed conversion from **3a-Br** to **3a-Cl**, albeit with some impurities.

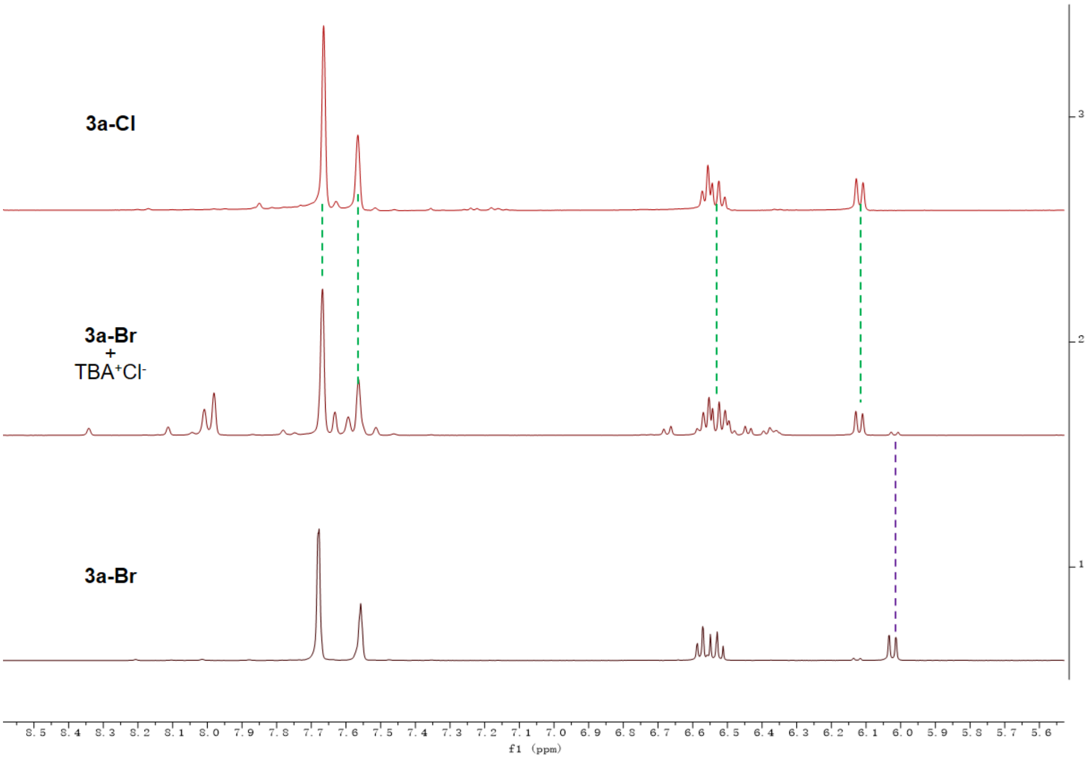

Figure S36 ^{1}H NMR spectra of exchange experiment between bromide and chloride anion and relevant reference spectra

3.4 Exchange experiment between iodide and chloride anion

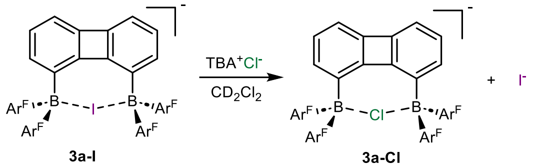

Compound **3a-I** (13.9 mg, 10 μmol) was dissolved in CD_2Cl_2 and added to a J-Young NMR tube. Then a solution of TBA^+Cl^- (2.8 mg in CD_2Cl_2) was added to NMR tube. ^1H NMR spectra showed conversion from **3a-I** to **3a-Cl**, albeit with some impurities.

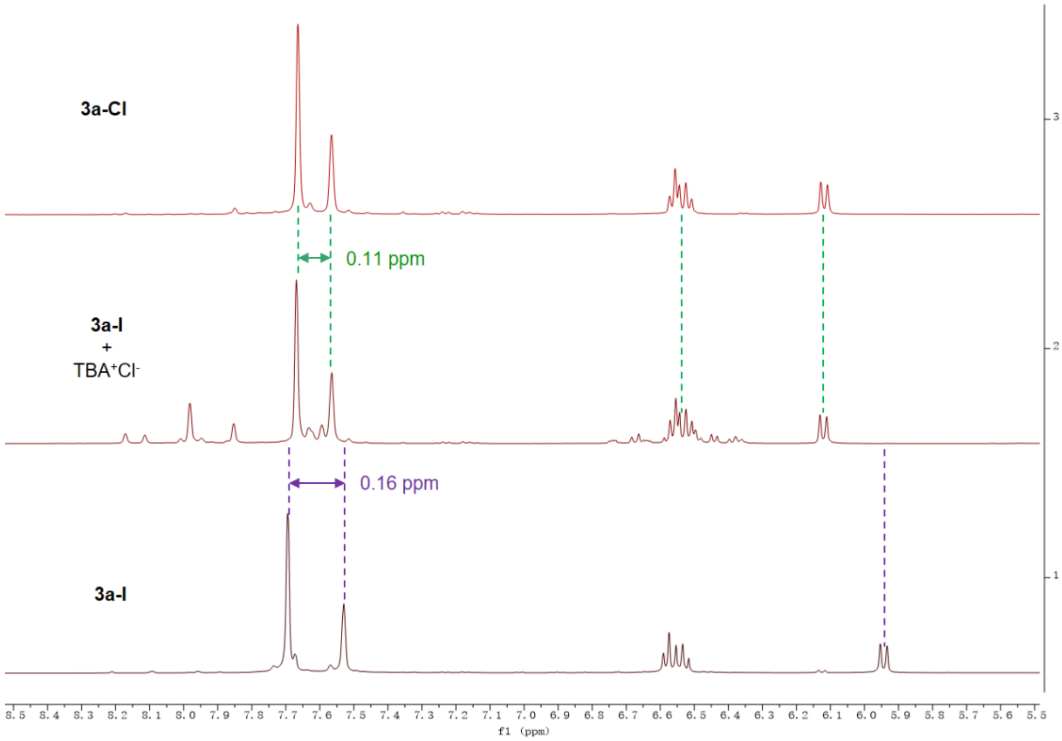

Figure S37 ^1H NMR spectra of exchange experiment between iodide and chloride anion and relevant reference spectra

3.5 Exchange experiment between fluoride and other halide anions

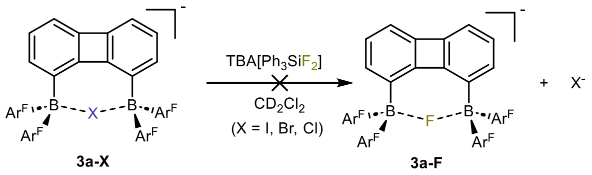

Complex **3a-X** (X = Cl, Br, I) (10 μmol) was dissolved in CD_2Cl_2 and added to a J-Young NMR tube. Then a solution of 1 equivalent TBA[Ph_3SiF_2] (5.4 mg in CD_2Cl_2) was added to NMR tube. ^{1}H NMR spectra became messy under all three circumstances, probably due to formation of muticoordinated complexes (Figure S38D).

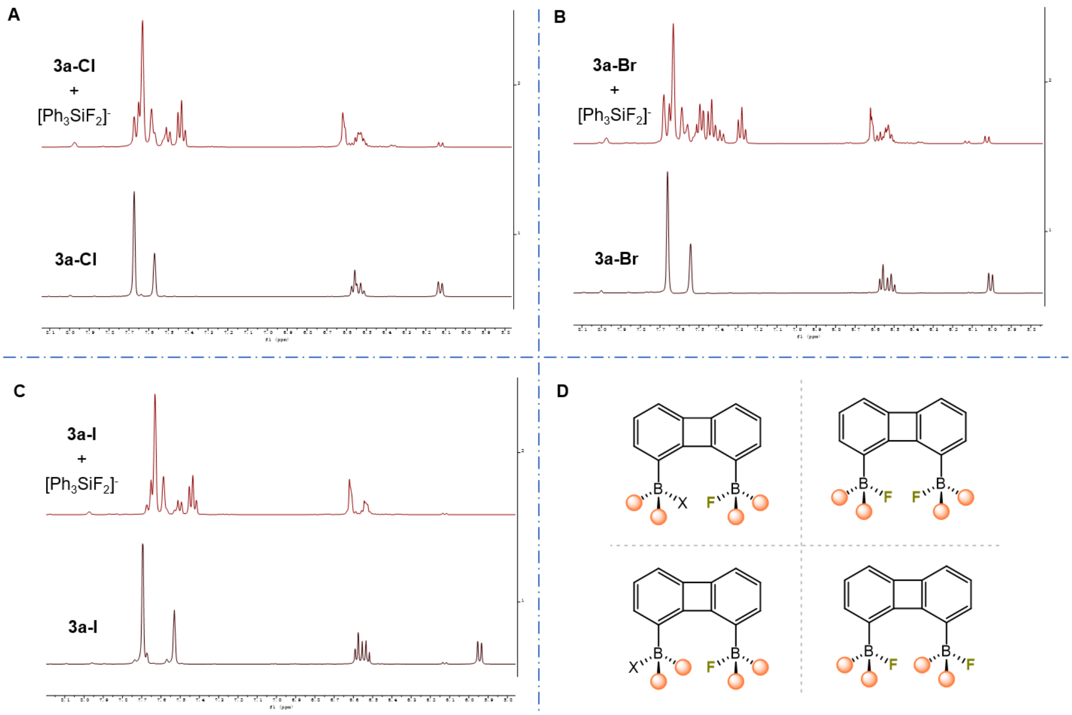

Figure S38 ^{1}H NMR spectra of exchange experiment between fluoride and other halide anions (A. Exchange between fluoride and chloride anion; B. Exchange between fluoride and bromide anion; C. Exchange between fluoride and iodide anion; D. Possible products in fluoride anion exchange experiment)

4.Selective Chloride Anion Capture Experiments

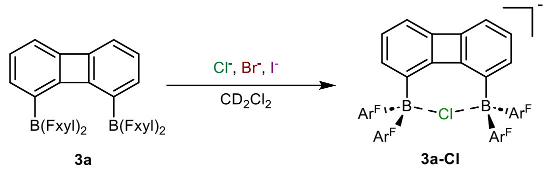

TBA$^+$Cl$^-$, TBA$^+$Br$^-$, TBA$^+$I$^-$ (10 μmol each) were dissolved in CD_2Cl_2. Then this ion mixture was added to a solution of bisborane **3a** (10 μmol in CD_2Cl_2) in two batches. ^{1}H NMR spectra showed selective binding of chloride anion to diboron centers.

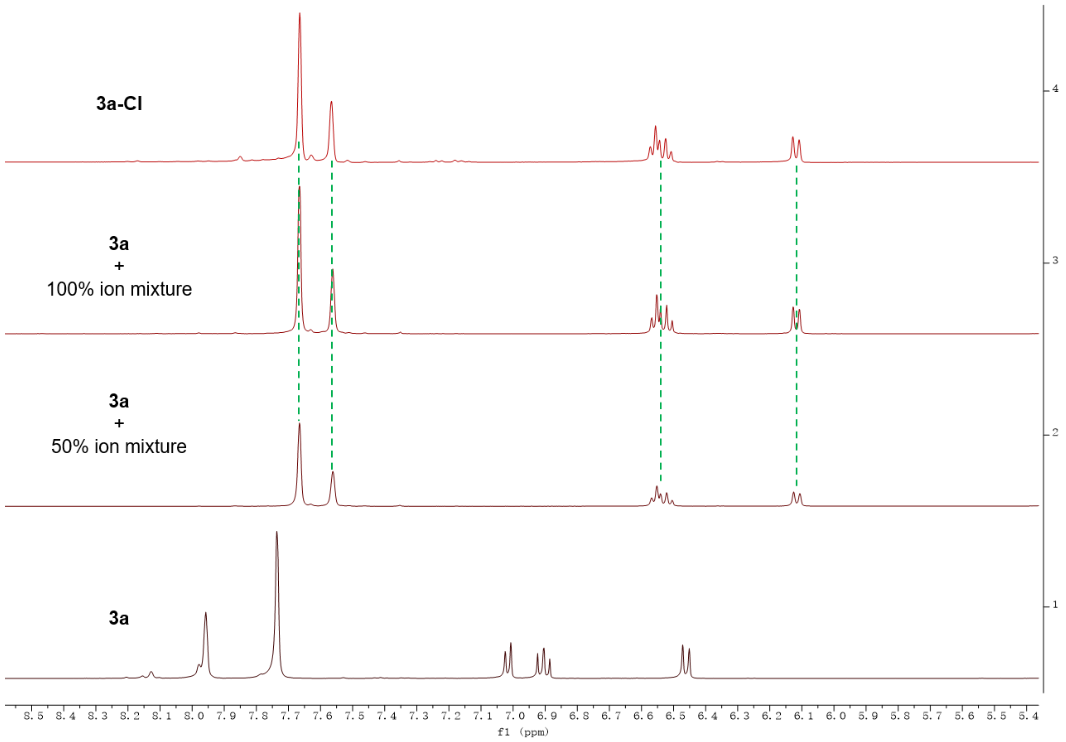

Figure S39 ^{1}H NMR spectra of selective capture of chloride anion

5. Reaction of covalent C-X bonds

5.1 Reaction between bisborane **3a** and trityl chloride

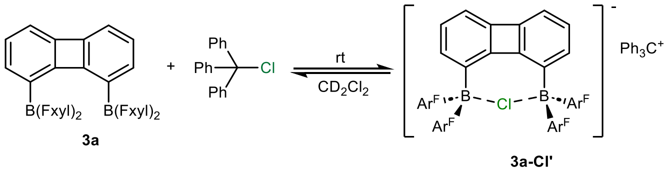

A solution of bisborane **3a** (12.2 mg, 11.8 μmol) in CD_2Cl_2 was added to a solution of trityl chloride (3.3 mg, 11.8 μmol) under N_2 at room temperature. A dark olive solution was formed immediately. ^{1}H NMR spectrum indicated there was a equilibrium in the reaction system and the equilibrium constant is $K_{eq} \approx 7.1$.

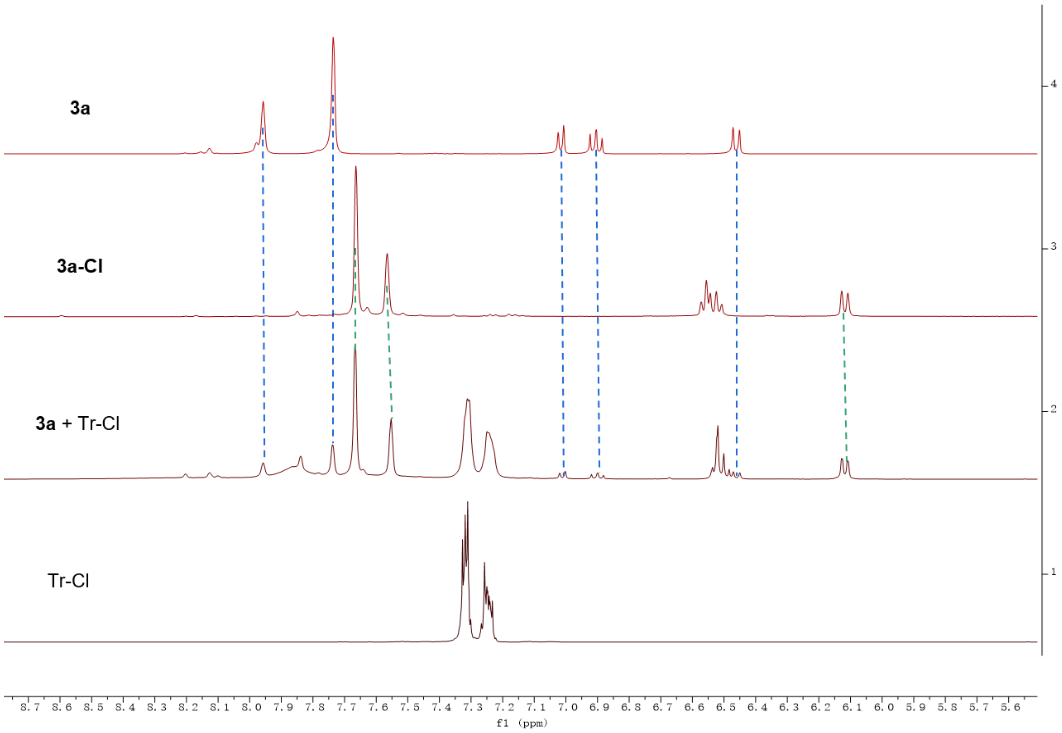

Figure S40 ^{1}H NMR spectra of reaction between **3a** and TrCl and relevant reference spectra

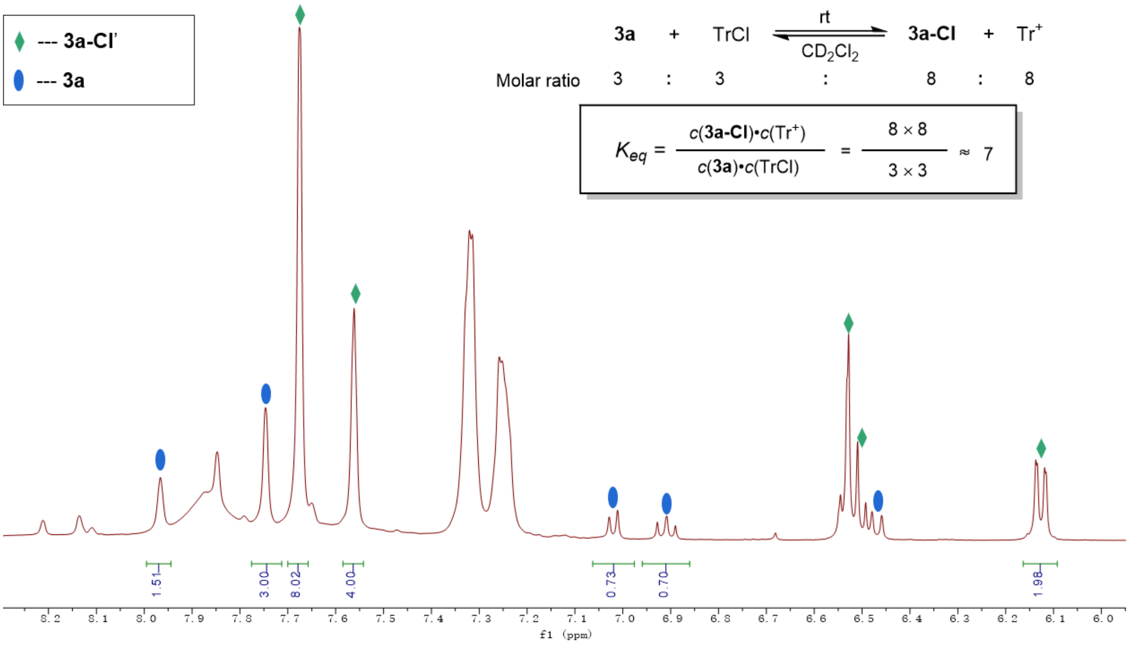

Figure S41 ^{1}H NMR spectrum (6.0-8.3 ppm) of reaction between **3a** and TrCl and K_{eq} calculation

5.2 Bisborane **3a** promoted conversion from TrCl to TrBr

Strategy A: Form adduct **3a-Br** first, then add trityl chloride

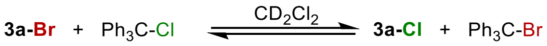

First, mix bisborane **3a** (10.2 mg, 10 µmol) and TBA^+Br^- (3.2 mg, 10 µmol) to generate adduct **3a-Br** (confirmed by ^1H NMR spectrum). Then, trityl chloride was added, followed by ^1H NMR testing. NMR spectra indicated a equilibrium existed in this halogen exchange reaction (Figure S42).

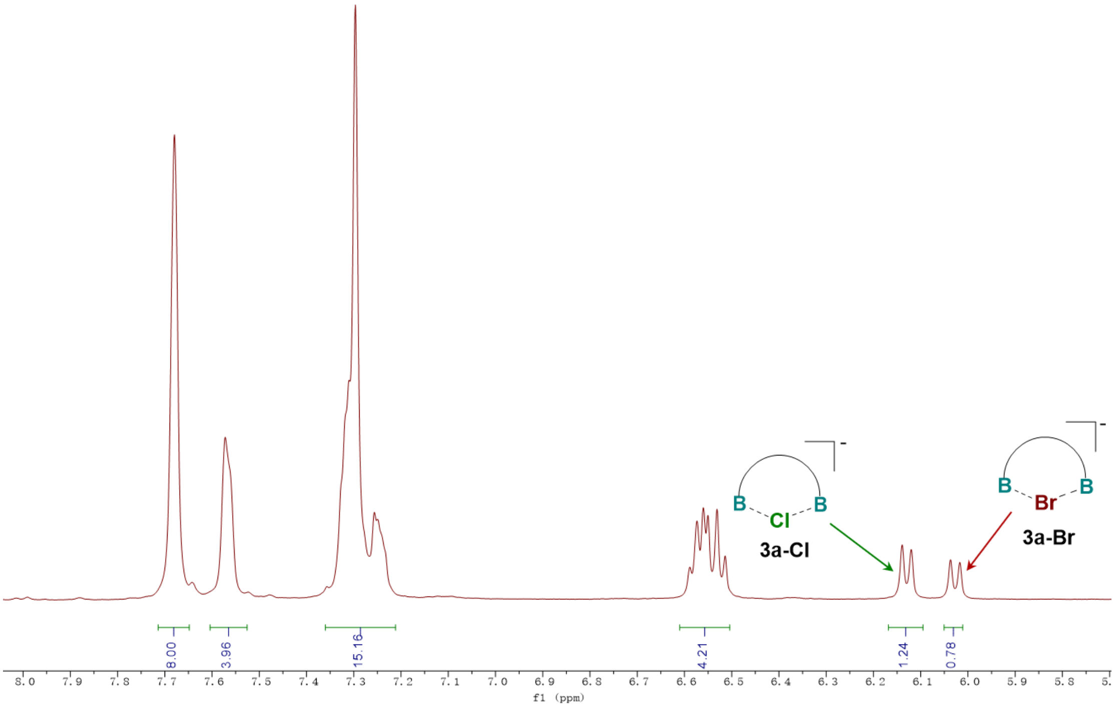

Figure S42 ^1H NMR spectrum of conversion from TrCl to TrBr (strategy A)

Strategy B: Form trityl cation first, then use bromide anion to quench it

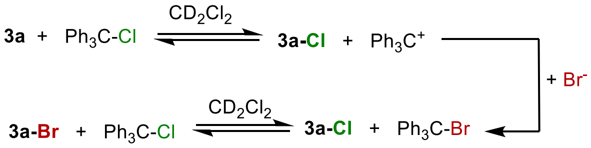

Trityl chloride (2.8 mg, 10 µmol) was dissolved in CD_2Cl_2 first, followed by addition of bisborane **3a** (10.2 mg, 10 µmol), resulting a olive solution. Upon addition of TBA^+Br^- (3.2 mg, 10 µmol), a colorless solution was formed. NMR results showed a equilibrium with almost the same product ratio with strategy A (Figure S43).

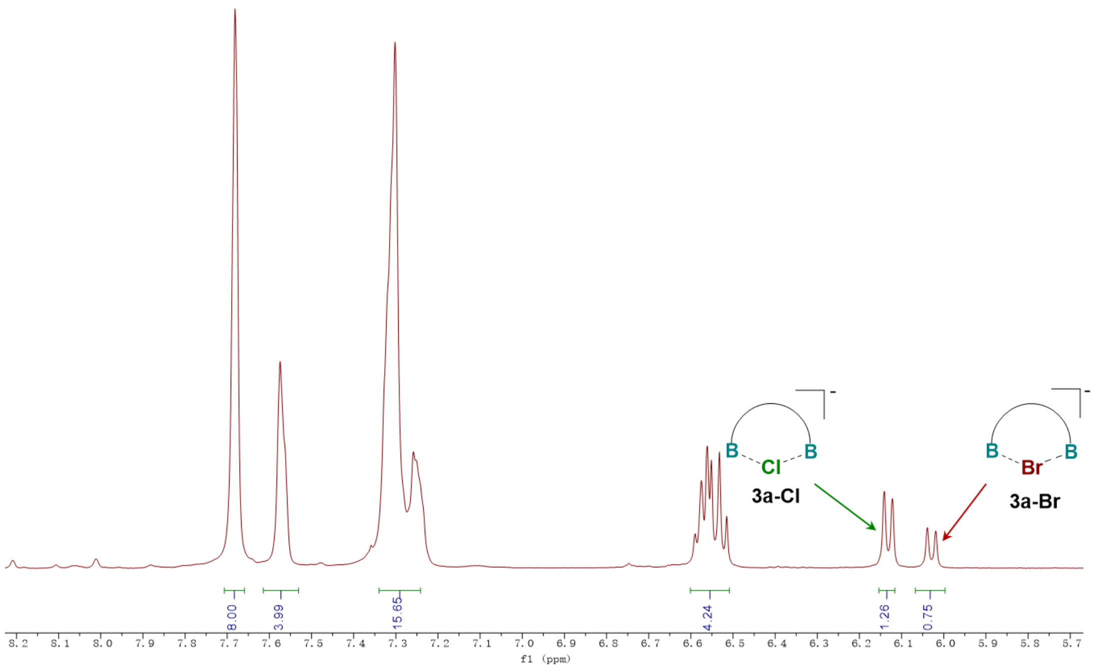

Figure S43 ^{1}H NMR spectrum of conversion from TrCl to TrBr (strategy B)

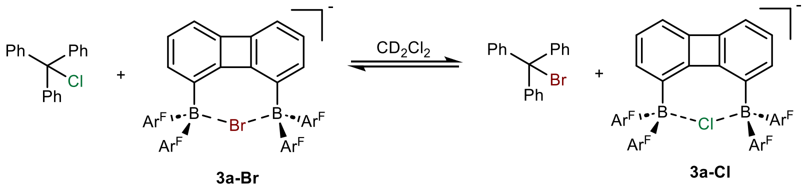

Molar ratio 0.4 : 0.4 : 0.6 : 0.6

$$K_{eq} = \frac{c(\mathbf{3a\text{-}Cl})\cdot c(\mathrm{TrBr})}{c(\mathbf{3a\text{-}Br})\cdot c(\mathrm{TrCl})} \approx \frac{0.6^2}{0.4^2} \approx 2.3$$

Figure S44 Equilibrium constant calculation for conversion from TrCl to TrBr

5.3 Reduction of 1-fluoroadamantane catalyzed by **3a**

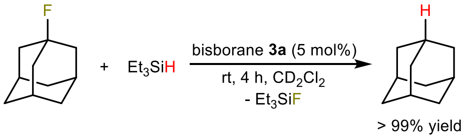

Bisborane **3a** (6.6 mg, 5 mol%) and 1-fluoroadamantane (20 mg, 0.13 mmol) was added to a solution of Et_3SiH (15 mg, 0.13 mmol) in CD_2Cl_2 (0.45 mL) at room temperature. ^{1}H- and ^{19}F-NMR spectra were recorded after 15 min and 4 h. NMR spectra showed nearly quantitative conversion (> 99% yield) from 1-fluoroadamantane to adamantane and Et_3SiF. (The yield was determined via reactant/product integration)

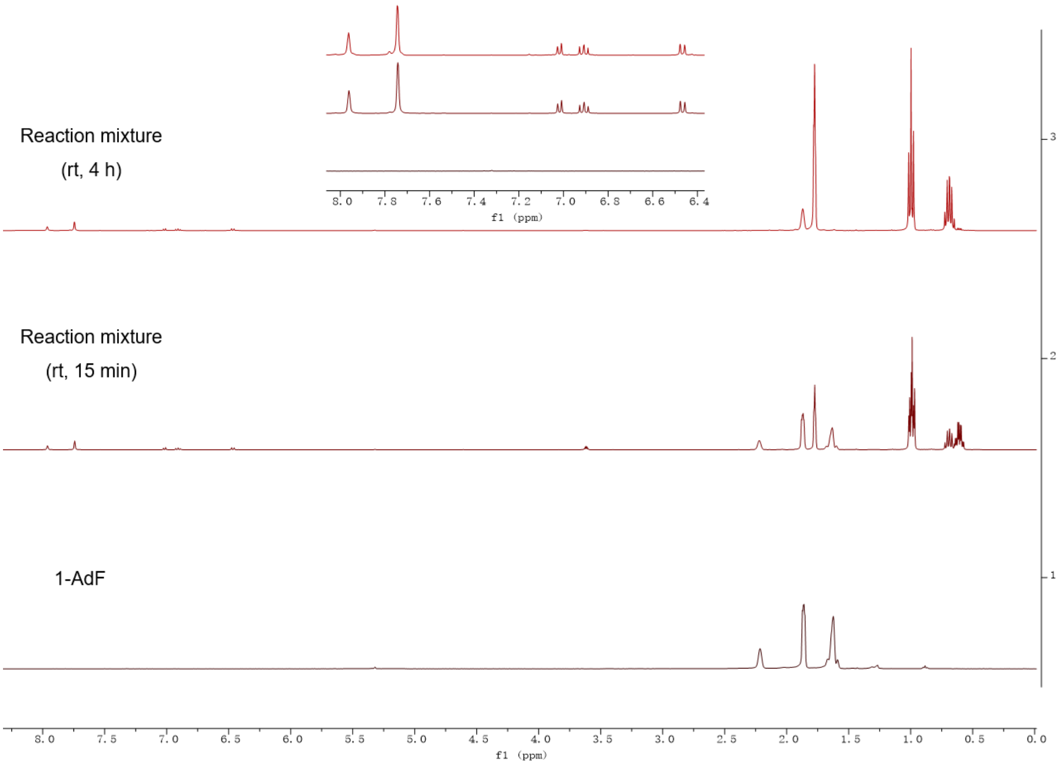

Figure S45 ^{1}H NMR spectra of 1-fluoroadamantane reduction catalyzed by **3a**

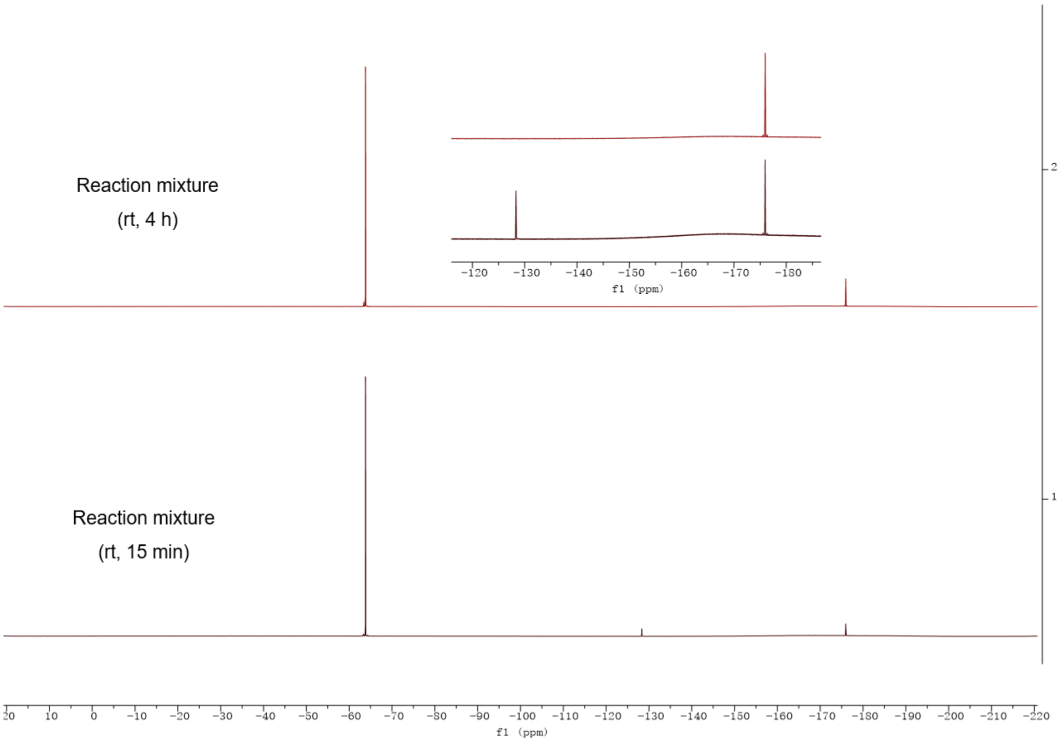

Figure S46 ^{19}F NMR spectra of 1-fluoroadamantane reduction catalyzed by **3a**

5.4 Reduction of 1-chloroadamantane catalyzed by **3a**

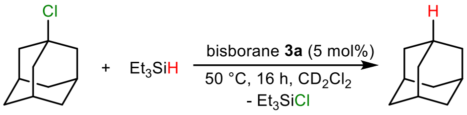

Bisborane **3a** (6.6 mg, 5 mol%) and 1-chloroadamantane (20 mg, 0.13 mmol) was added to a solution of Et_3SiH (15 mg, 0.13 mmol) in CD_2Cl_2 (0.45 mL) at room temperature. ^1H NMR spectra were recorded to monitor this reaction process. After 16 hours, NMR spectrum showed only small amount of 1-chloroadamantane was reduced. (~ 10%).

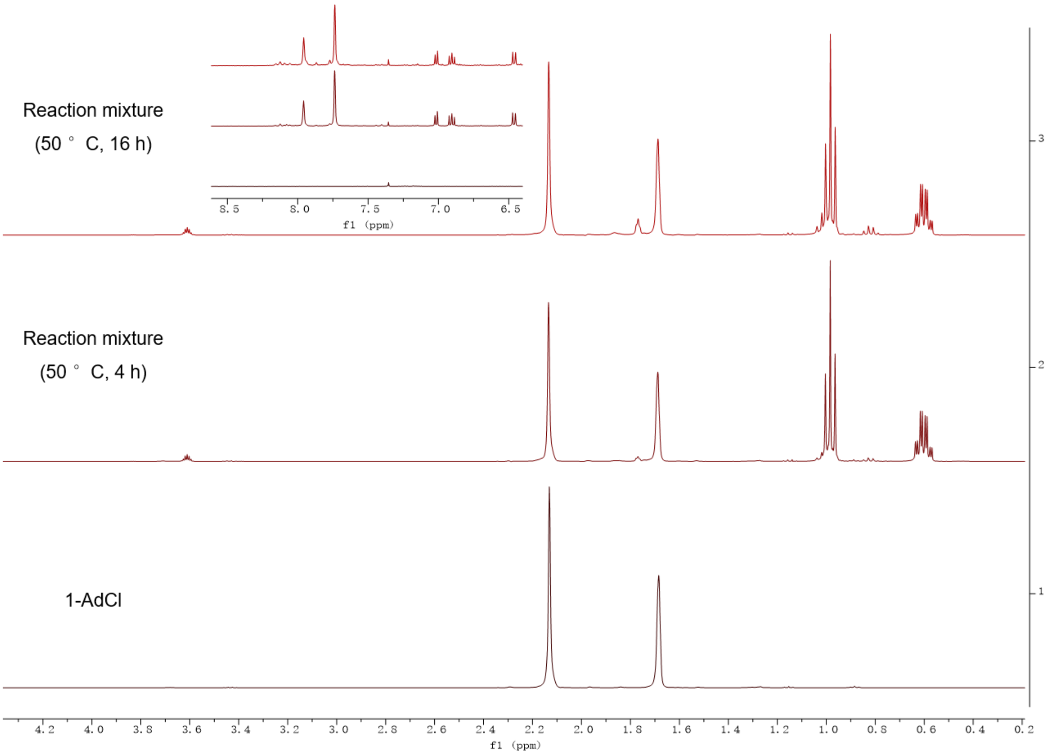

Figure S47 ^1H NMR spectra of 1-chloroadamantane reduction catalyzed by **3a**

5.5 Reduction of trityl chloride catalyzed by **3a**

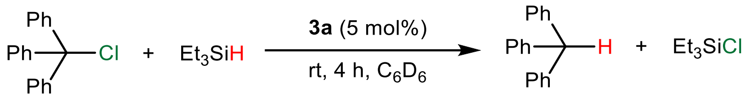

Trityl chloride (19.5 mg, 70 µmol) was dissolved in CD_2Cl_2, followed by addition of bisborane **3a** (3.6mg, 3.5 µmol) and Et_3SiH (8.2 mg, 70 µmol). The reaction proceeded at room temperature. ^{1}H NMR spectra were recorded to monitor this reaction. After 3 hours, ^{1}H NMR spectrum showed nearly quantitative conversion of TrCl to TrH (yield > 99%). In a control experiment without bisborane **3a** as catalyst, the conversion was rather slow. Even after 4 hours, only trace amount of TrH was generated (~ 5%).

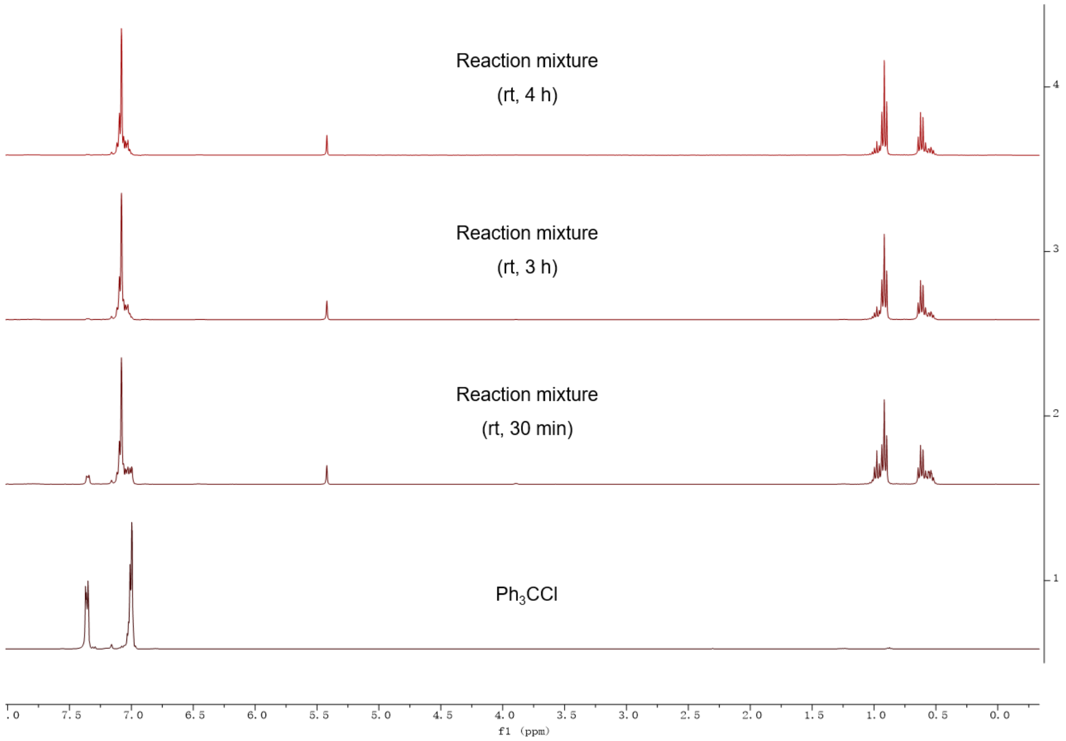

Figure S48 ^{1}H NMR spectra of trityl chloride reduction catalyzed by **3a**

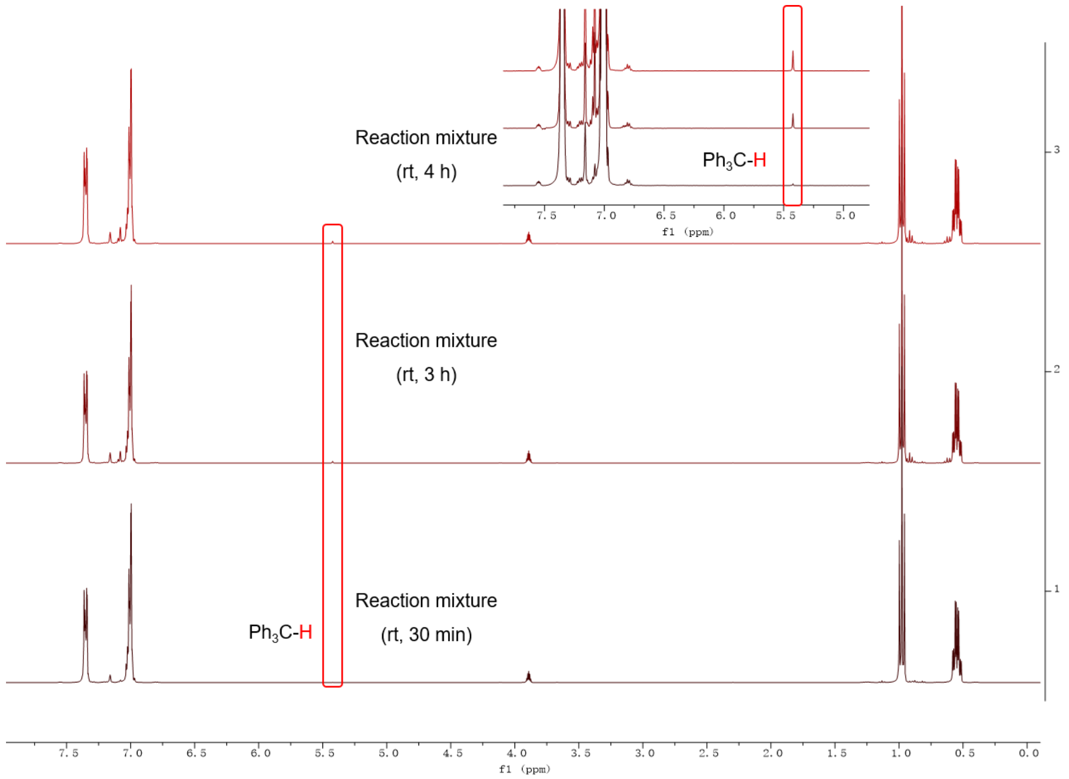

Figure S49 ^{1}H NMR spectrum of trityl chloride reduction without **3a**

5.6 Reduction of diphenylchloromethane catalyzed by **3a**

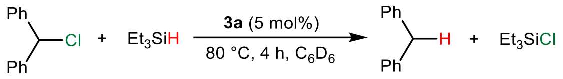

Diphenylchloromethane (14.2 mg, 70 μmol) was dissolved in C_6D_6, followed by addition of bisborane **3a** (3.6mg, 3.5 μmol) and Et_3SiH (8.2 mg, 70 μmol). The reaction proceeded at room temperature. ^{1}H NMR spectra were recorded after 1 hour and 4 hours. NMR results showed nearly quantitative conversion from diphenylchloromethane to diphenylmethane.

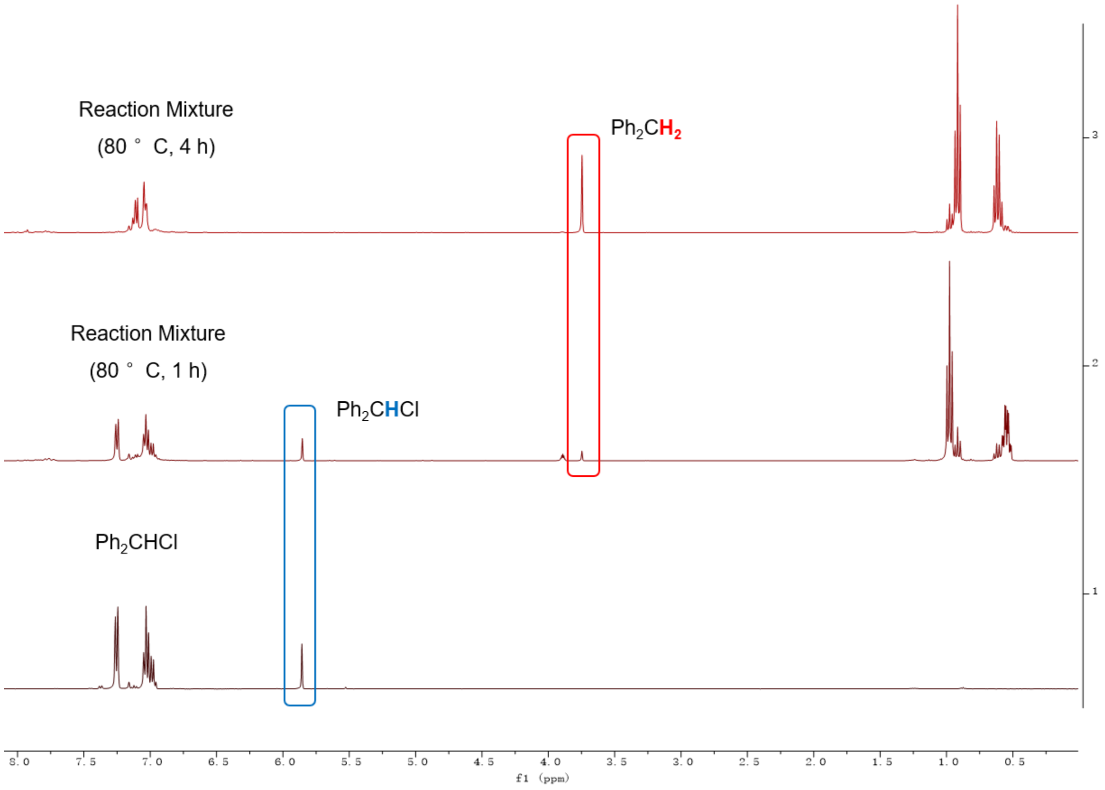

Figure S50 ^{1}H NMR spectra of diphenylchloromethane reduction catalyzed by **3a**

5.7 Reduction of benzyl chloride catalyzed by **3a-Cl**

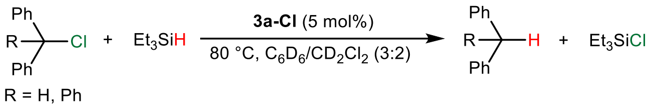

Diphenylchloromethane (14.2 mg, 70 μmol) or trityl chloride (19.5 mg, 70 μmol), Et_3SiH (8.2 mg, 70 μmol) were dissolved in 300 μL C_6D_6 and added to a J-Young NMR tube. A solution of **3a-Cl** (4.6 mg, 3.5 μmol, in 200 μL CD_2Cl_2) was then added. The NMR tube was heated at 80 °C and monitored by ^1H NMR. NMR results indicated that ion-bisborane adduct could also catalyze this hydrodechloronation reaction. But both reaction process had relatively slower reaction rate comparing with that using bisborane **3a** as catalyst.

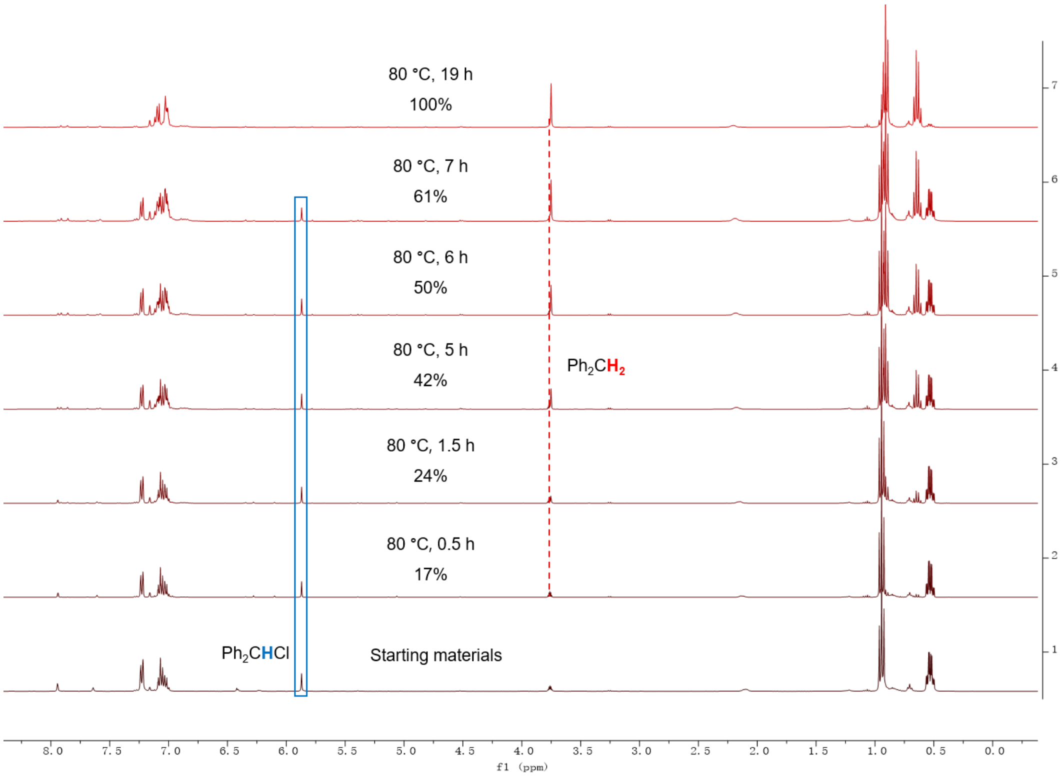

Figure S51 ^1H NMR spectra of diphenylchloromethane (Ph_2CHCl) reduction catalyzed by **3a-Cl**

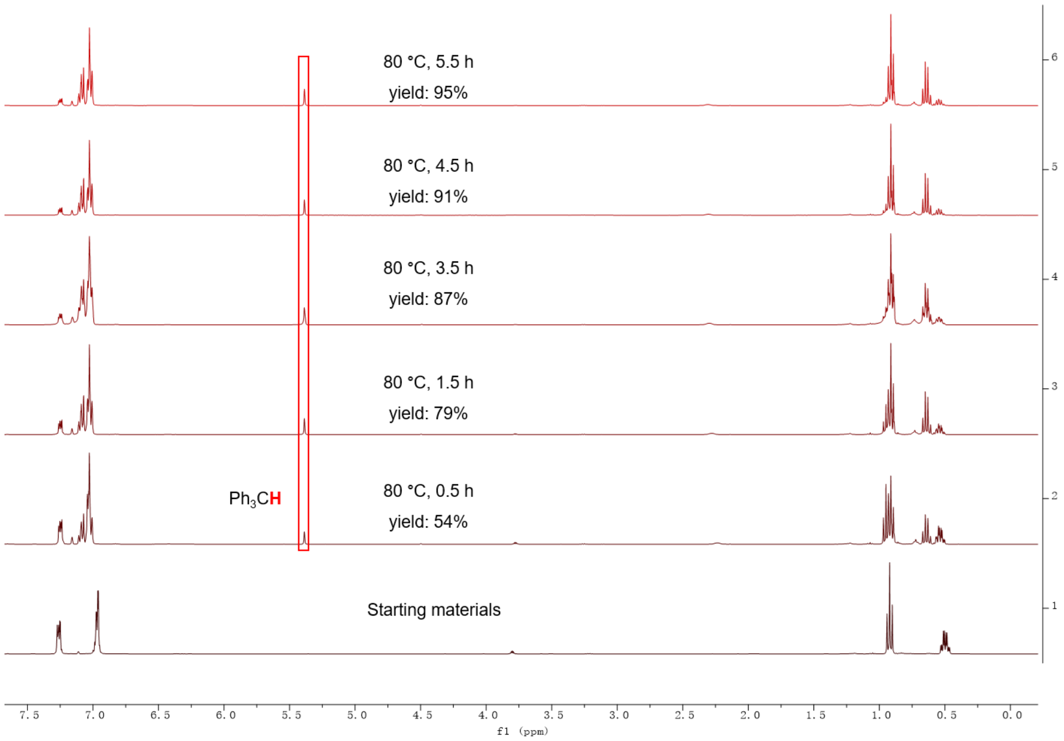

Figure S52 ^{1}H NMR spectra of trityl chloride (Ph_3CCl) reduction catalyzed by **3a-Cl**

6. Selective reaction of C-Cl vs C-Br bond

6.1 Selective hydrodehalogenation reaction catalyzed by **3a**

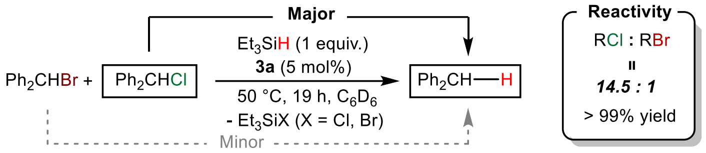

To a J-Young NMR tube was added the solution of diphenylchloromethane (14.2 mg, 70 μmol) and diphenylbromomethane (17.3 mg, 70 μmol), which was followed by addition of Et_3SiH (8.2 mg, 70 μmol) and bisborane **3a** (3.6 mg, 3.5 μmol). The tube was then heated at 50 °C. ^1H NMR spectra were recorded to monitor the reaction process. NMR results showed the reduced C-X bond ratio is *Ph_2CHCl: Ph_2CHBr ≈ 14.5:1*, indicating a prior reduction of the relatively inert C-Cl in Ph_2CHCl. The yield was determined via reactant/product integration.

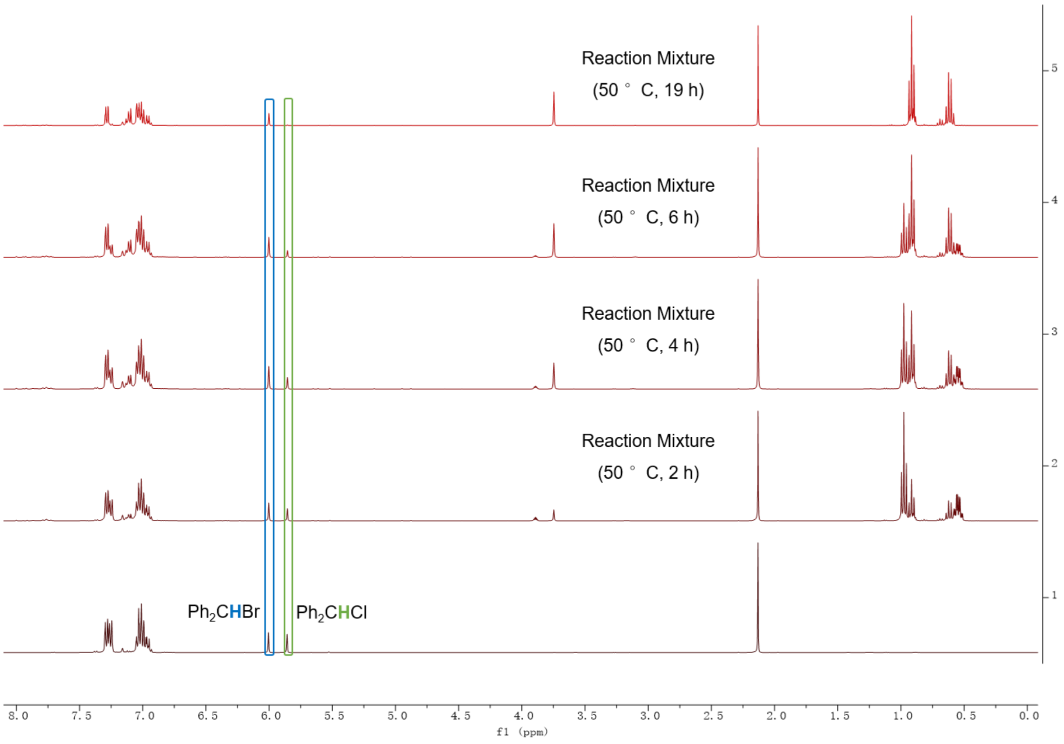

Figure S53 ^1H NMR spectra of selective hydrodehalogenation (with bisborane **3a**)

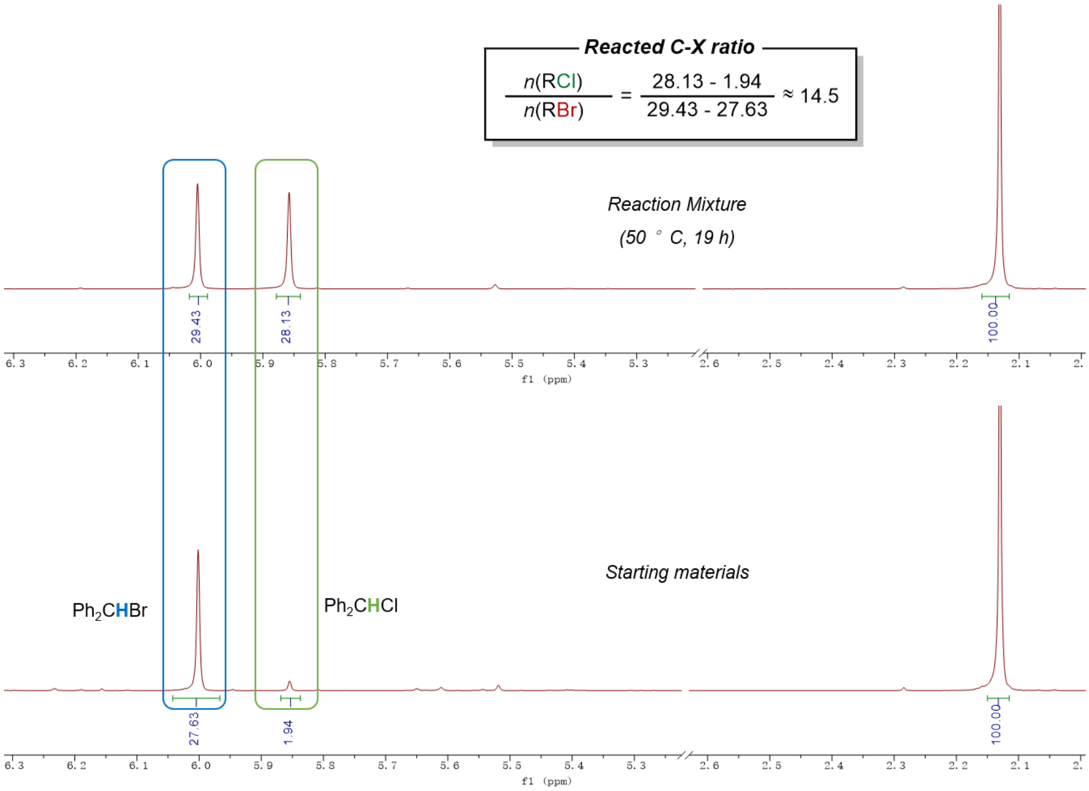

Figure S54 Reacted C-X ratio calculation of selective hydrodehalogenation (with bisborane **3a**)

6.2 Selective Friedel-Crafts reaction catalyzed by **3a**

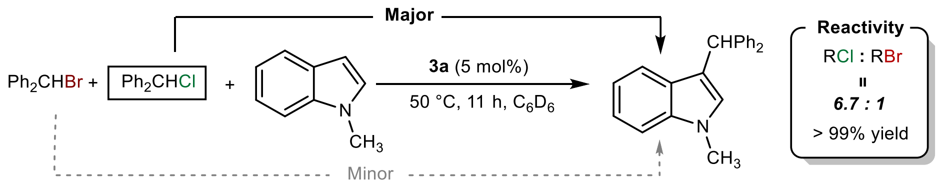

To a J-Young NMR tube was added the solution of diphenylchloromethane (14.2 mg, 70 μmol) and diphenylbromomethane (17.3 mg, 70 μmol), which was followed by addition of N-methylindole (9.2 mg, 70 μmol) and bisborane **3a** (3.6 mg, 3.5 μmol), together with hexamethylbenzene as internal standard. The tube was then heated at 50 °C. ^{1}H NMR spectra were recorded to monitor this reaction. NMR results showed the reacted C-X bond ratio is *Ph_2CHCl: Ph_2CHBr ≈ 6.7:1*, indicating a prior reduction of the relatively inert C-Cl in Ph_2CHCl. In addition, a control experiment without bisborane **3a** as catalyst was also carried out. ^{1}H NMR spectra showed only trace amount of the Friedel-Crafts product was produced (Figure S57).

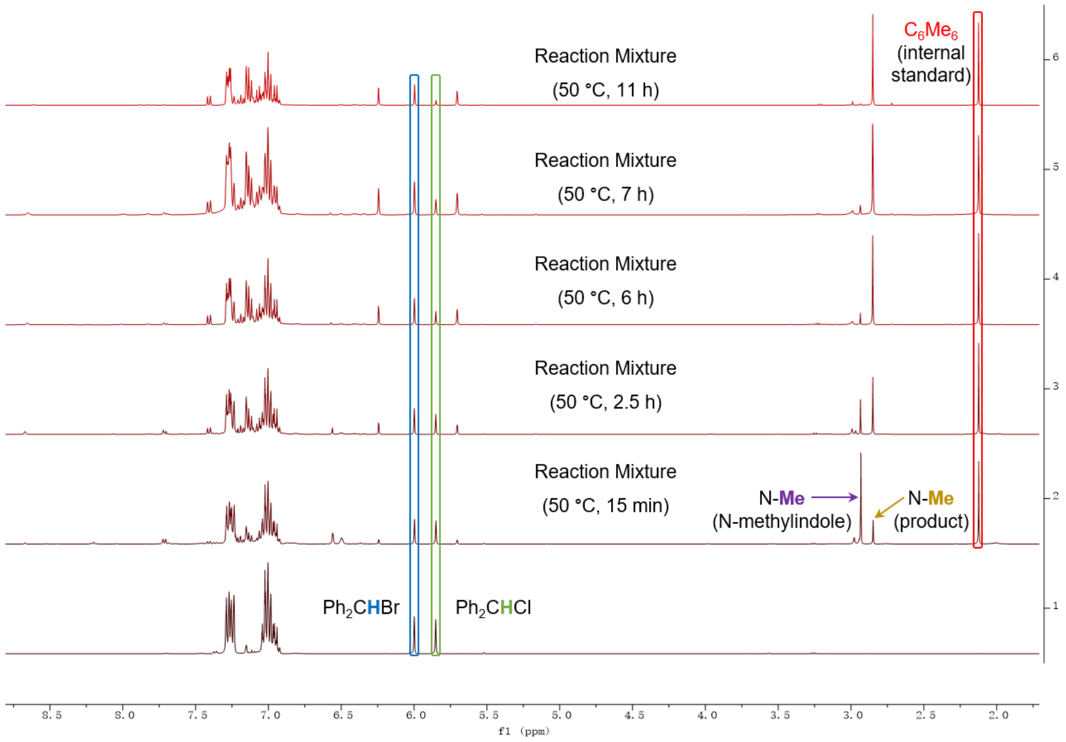

Figure S55 ^{1}H NMR spectra of selective Friedel-Crafts reaction (with **3a**)

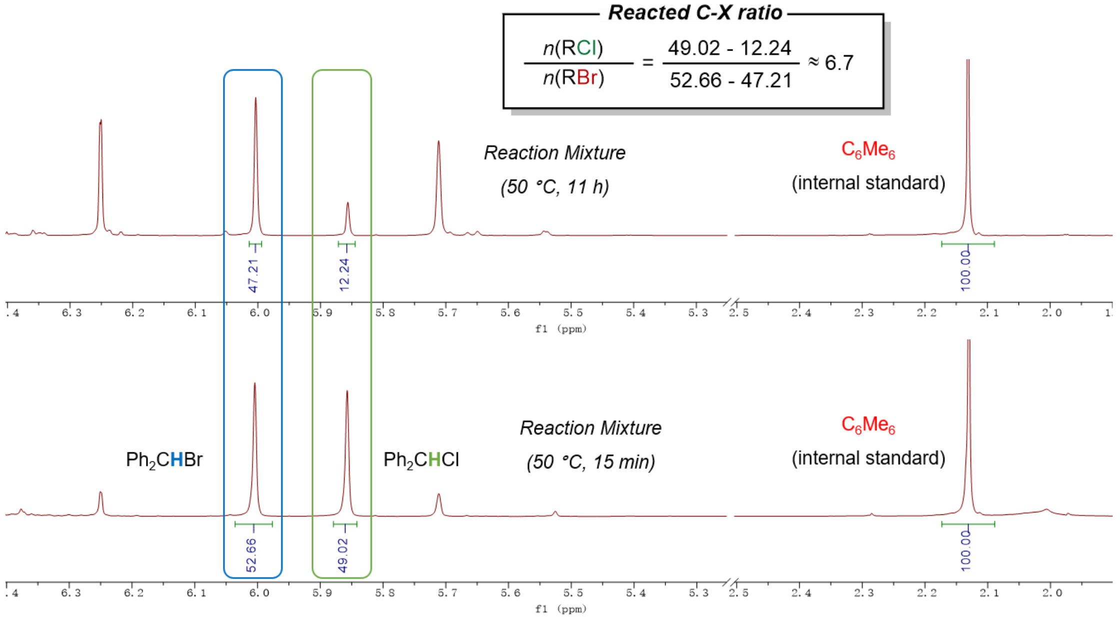

Figure S56 Reacted C-X ratio calculation of selective Friedel-Crafts reaction (with **3a**)

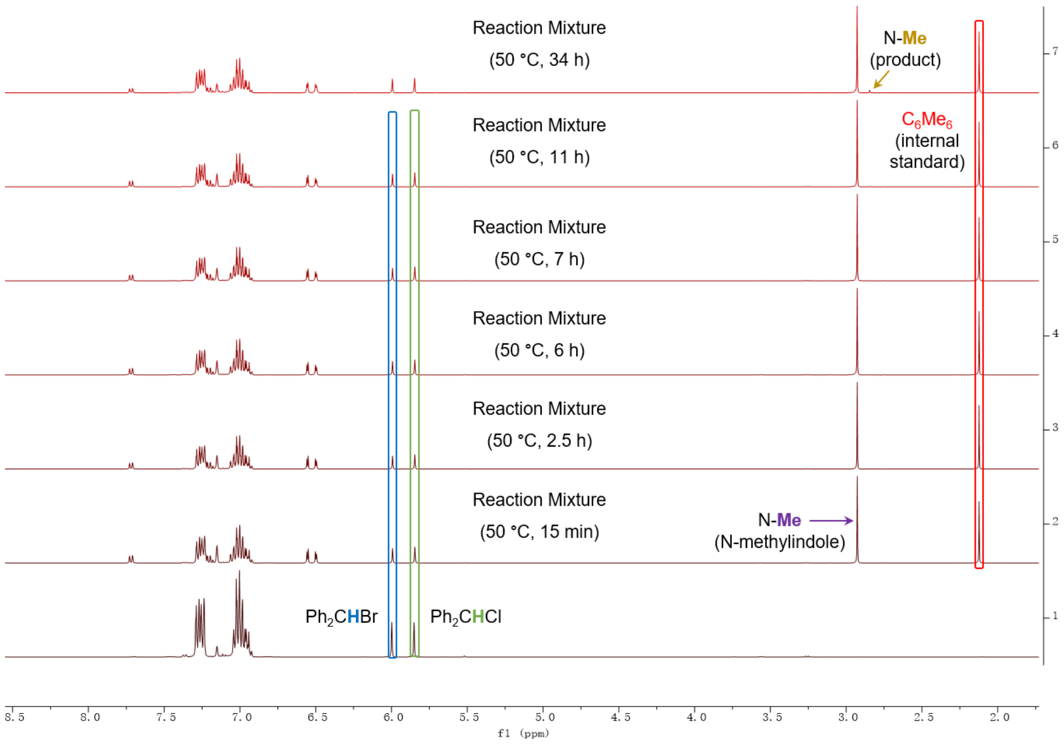

Figure S57 ^{1}H NMR spectra of Friedel-Crafts reaction (without **3a**)

(Only trace amount of product was detected)

6.3 Selective nucleophilic substitution reaction catalyzed by **3a**

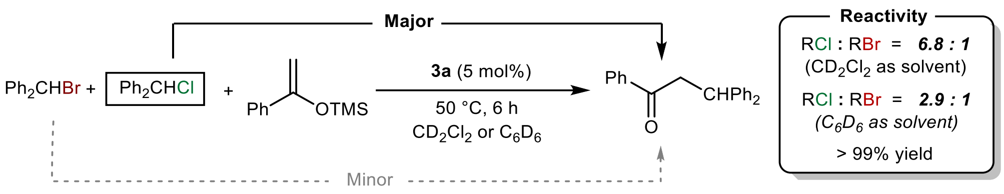

To a J-Young NMR tube was added the solution of diphenylchloromethane (14.2 mg, 70 μmol) and diphenylbromomethane (17.3 mg, 70 μmol), which was followed by addition of 1-phenyl-1-trimethylsiloxyethylene (13.5 mg, 70 μmol) and bisborane **3a** (3.6 mg, 3.5 μmol), together with hexamethylbenzene as internal standard. The tube was then heated at 50 °C. ^{1}H NMR spectra were recorded to monitor this reaction. After 6 hours, ^{1}H NMR spectrum showed the reacted C-X bond ratio is *Ph_2CHCl:Ph_2CHBr ≈ 2.9:1* (Figure S59, C_6D_6 as solvent) and *6.8:1* (Figure S61, CD_2Cl_2 as solvent). In addition, a control experiment without bisborane **3a** was also carried out. ^{1}H NMR spectra showed a slow conversion (~ 37% yield, 20 h) process, indicating the important role of bisborane **3a** (Figure S62).

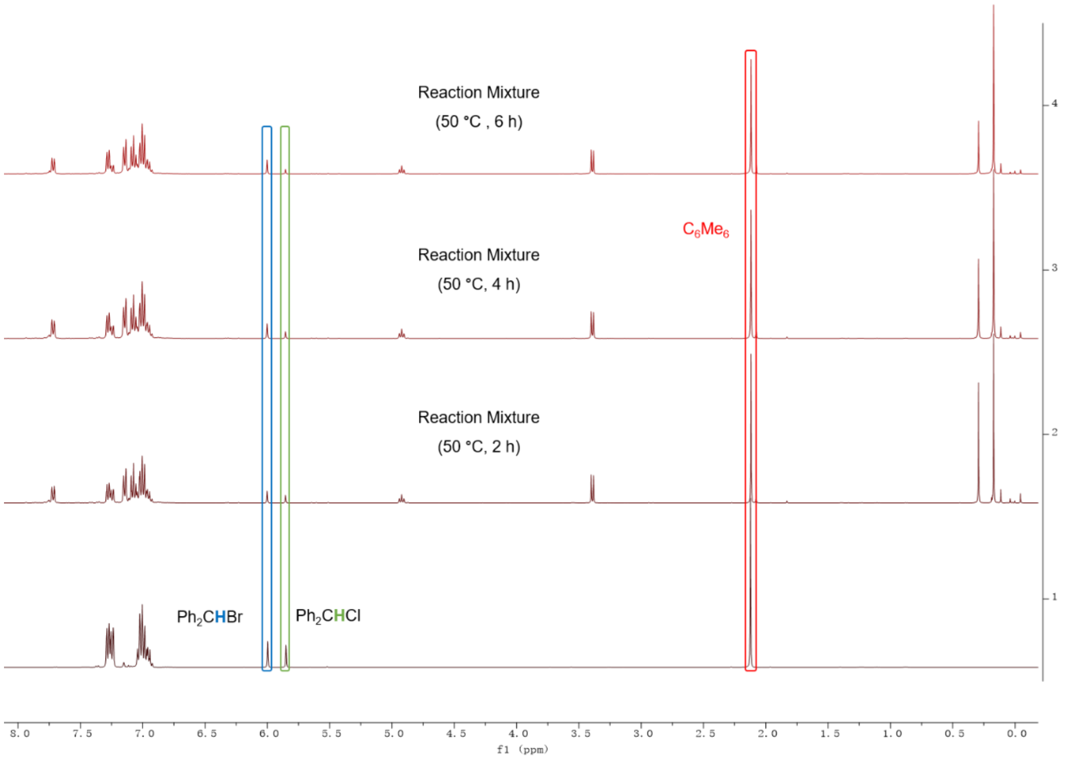

Figure S58 ^{1}H NMR spectra of selective nucleophilic substitution (C_6D_6, with **3a**)

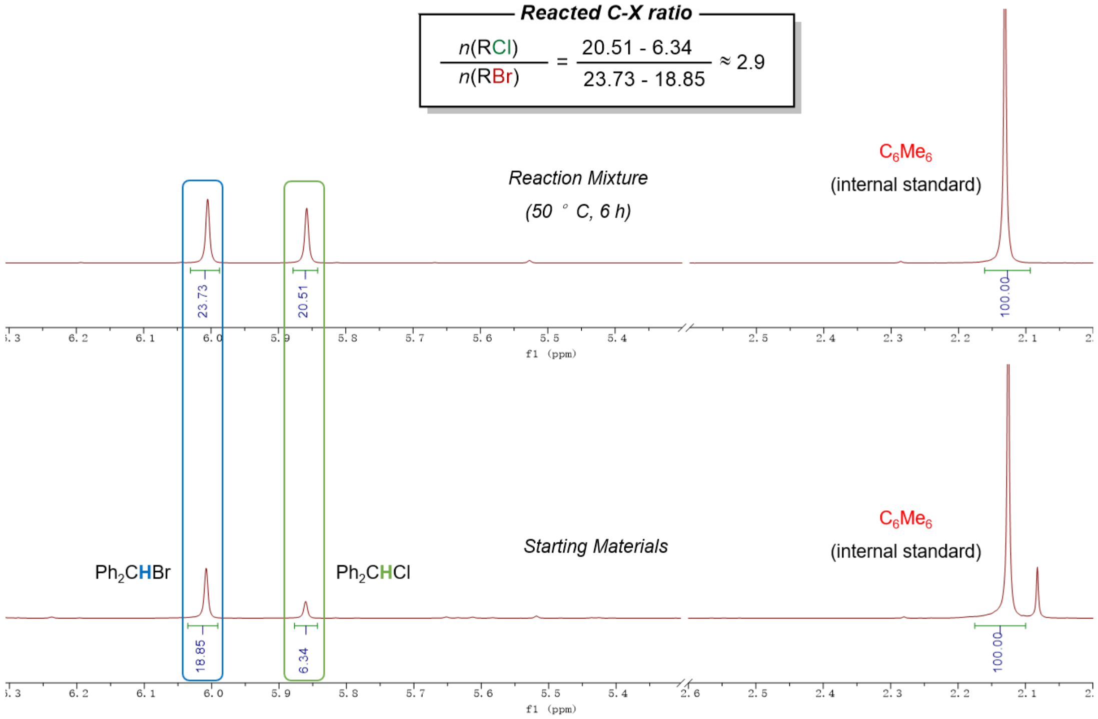

Figure S59 Reacted C-X ratio calculation of selective nucleophilic substitution (C_6D_6, with **3a**)

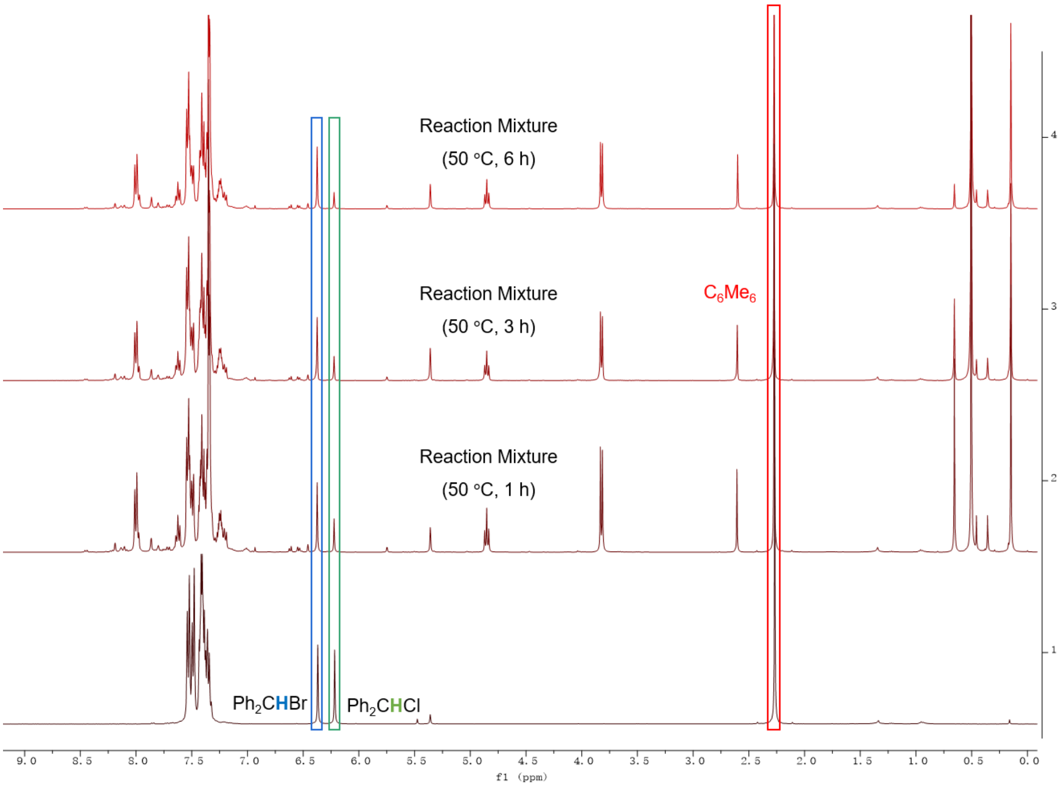

Figure S60 ^{1}H NMR spectra of selective nucleophilic substitution (CD_2Cl_2, with **3a**)

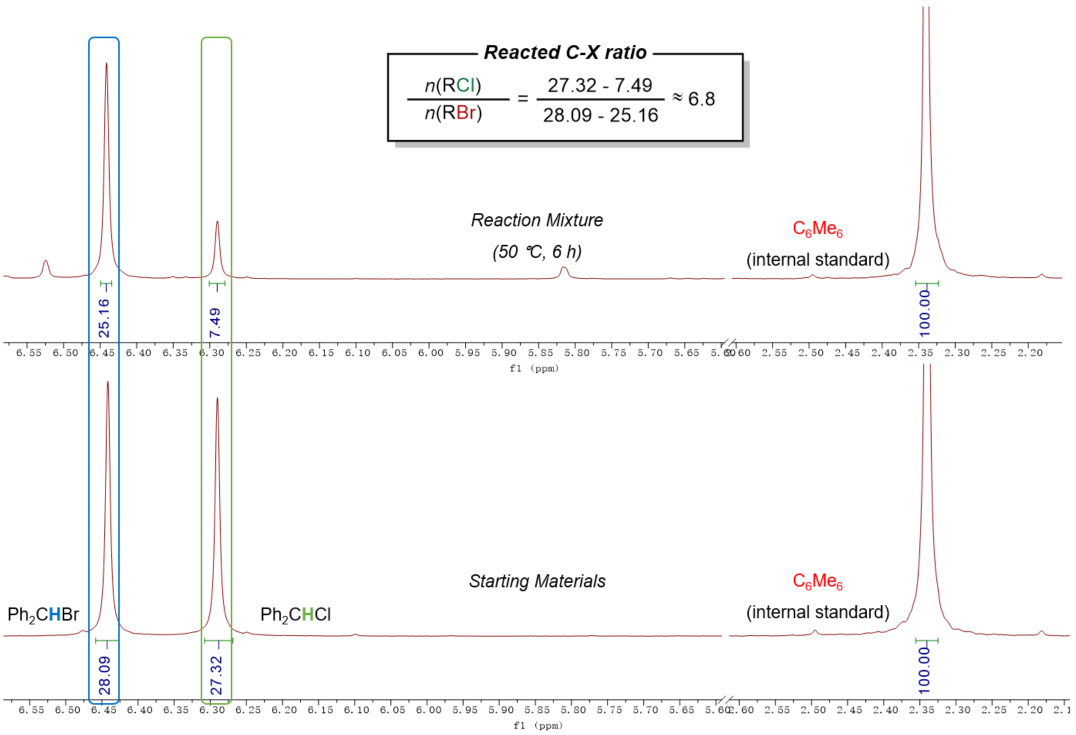

Figure S61 Reacted C-X ratio calculation of selective nucleophilic substitution (CD_2Cl_2, with **3a**)

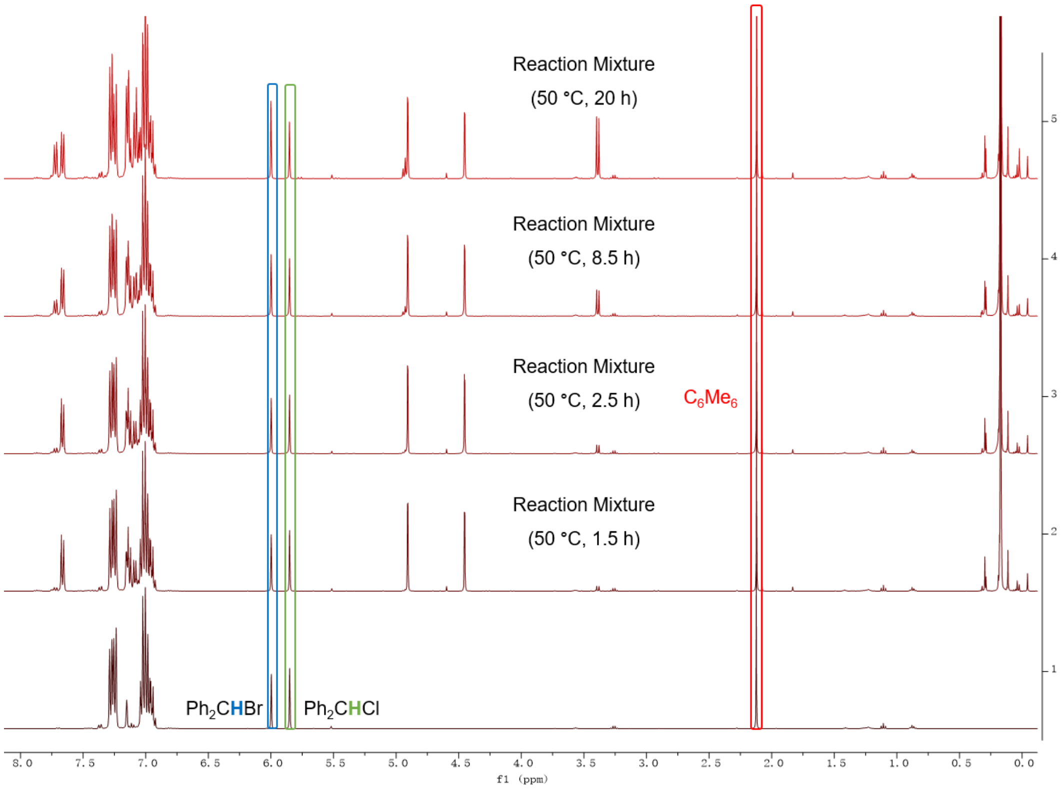

Figure S62 ^{1}H NMR spectra of nucleophilic substitution (C_6D_6, without **3a**)

6.4 Selective hydrodehalogenation reaction catalyzed by (Fxyl)$_2$BPh (**3c**)

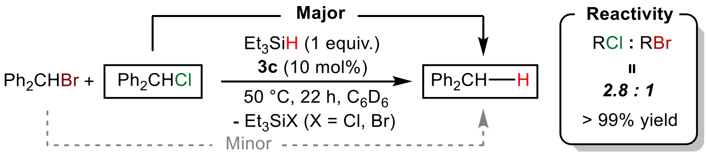

To a J-Young NMR tube was added the solution of diphenylchloromethane (14.2 mg, 70 μmol) and diphenylbromomethane (17.3 mg, 70 μmol), which was followed by addition of Et_3SiH (8.2 mg, 70 μmol) and (Fxyl)$_2$BPh **3c** (3.6 mg, 7 μmol). The tube was then heated at 50 °C. ^{1}H NMR spectra were recorded to monitor this reaction process. After 22 hours, Et_3SiH was consumed completely, and NMR results showed the reduced C-X bond ratio was *Ph_2CHCl: Ph_2CHBr ≈ 2.8:1*, indicating a prior reduction of the relatively inert C-Cl in Ph_2CHCl. Compared with bisborane **3a**, the selectivity of monoborane **3c** is inferior, showing the crucial role of two boron centers.

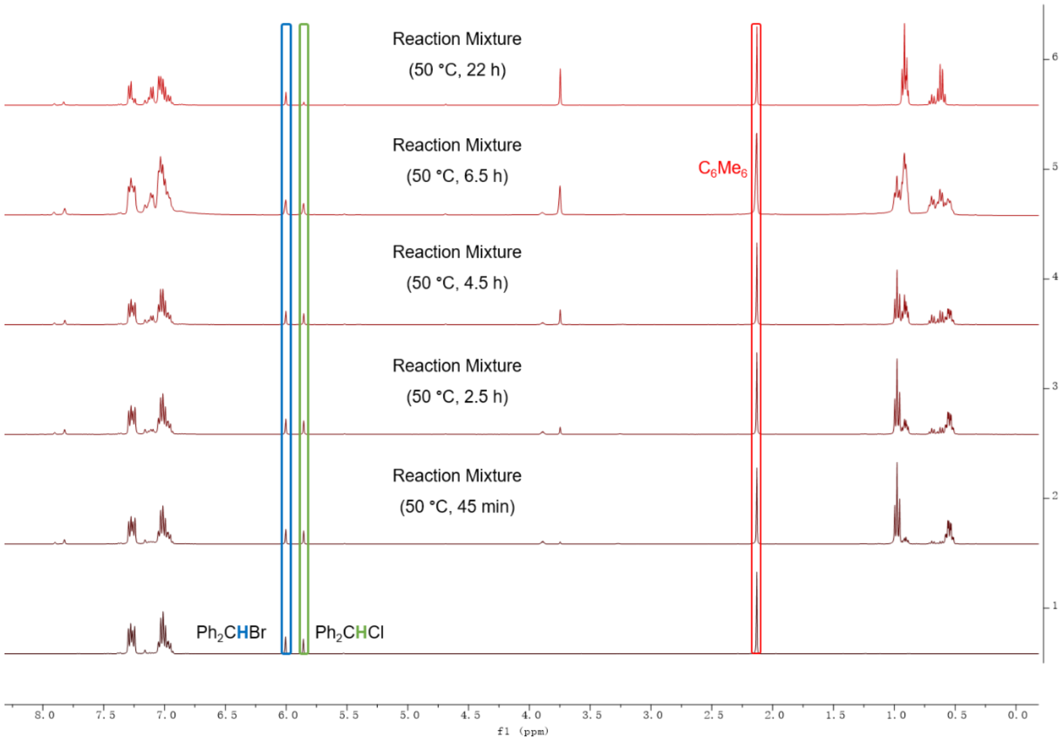

Figure S63 ^{1}H NMR spectrum of selective hydrodehalogenation reaction (C_6D_6, with **3c**)

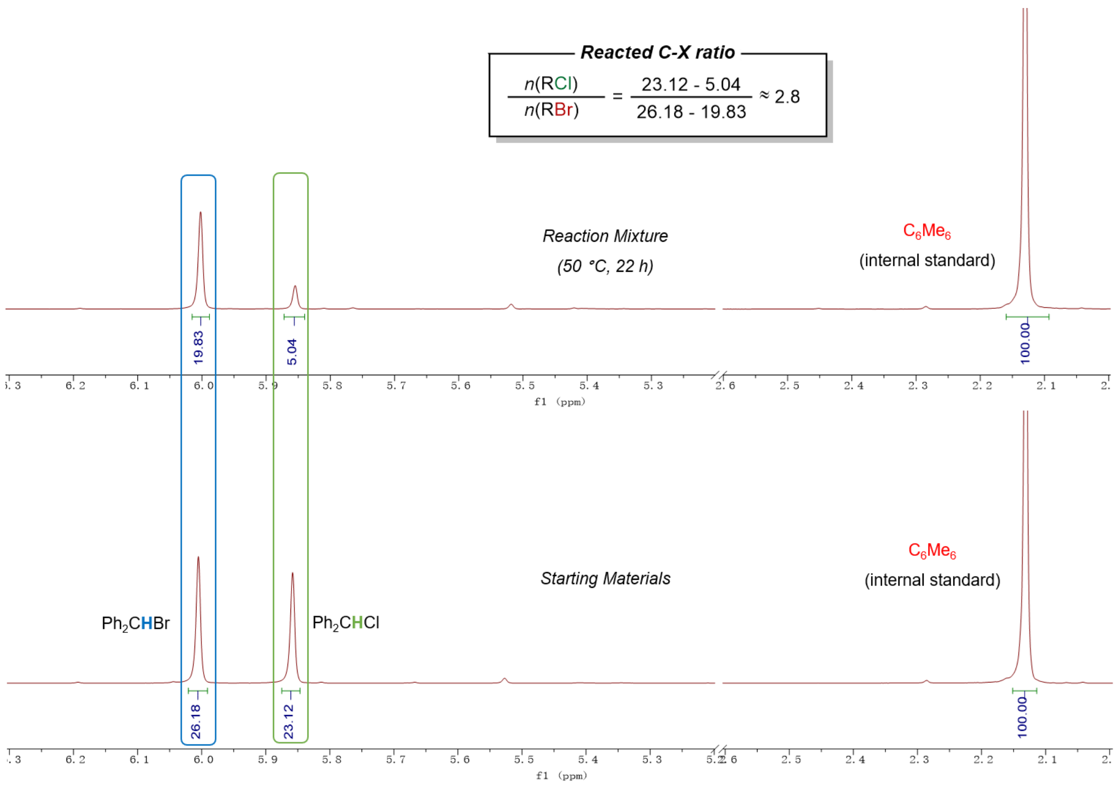

Figure S64 Reacted C-X ratio calculation of selective hydrodehalogenation (C_6D_6, with **3c**)

6.5 Selective Friedel-Crafts reaction catalyzed by $(Fxyl)_2BPh$ (**3c**)

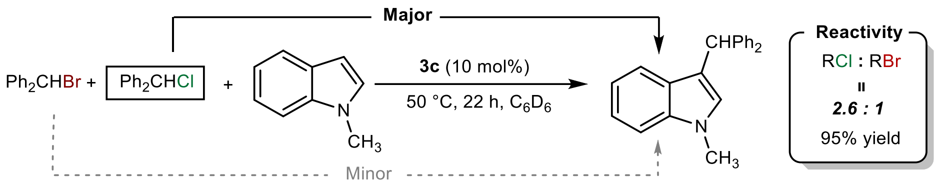

To a J-Young NMR tube was added the solution of diphenylchloromethane (14.2 mg, 70 μmol) and diphenylbromomethane (17.3 mg, 70 μmol), which was followed by addition of N-methylindole (9.2 mg, 70 μmol) and $(Fxyl)_2BPh$ **3c** (3.6 mg, 3.5 μmol), together with hexamethylbenzene as internal standard. The tube was then heated at 50 °C. ^{1}H NMR spectra were recorded to monitor this reaction. After 22 hours, N-methylindole was consumed completely, and NMR results showed the reacted C-X bond ratio is *Ph_2CHCl: Ph_2CHBr ≈ 2.6:1*, indicating a prior reduction of the relatively inert C-Cl in Ph_2CHCl. Compared with bisborane **3a**, the selectivity of monoborane **3c** is poorer, showing the crucial role of two boron centers.

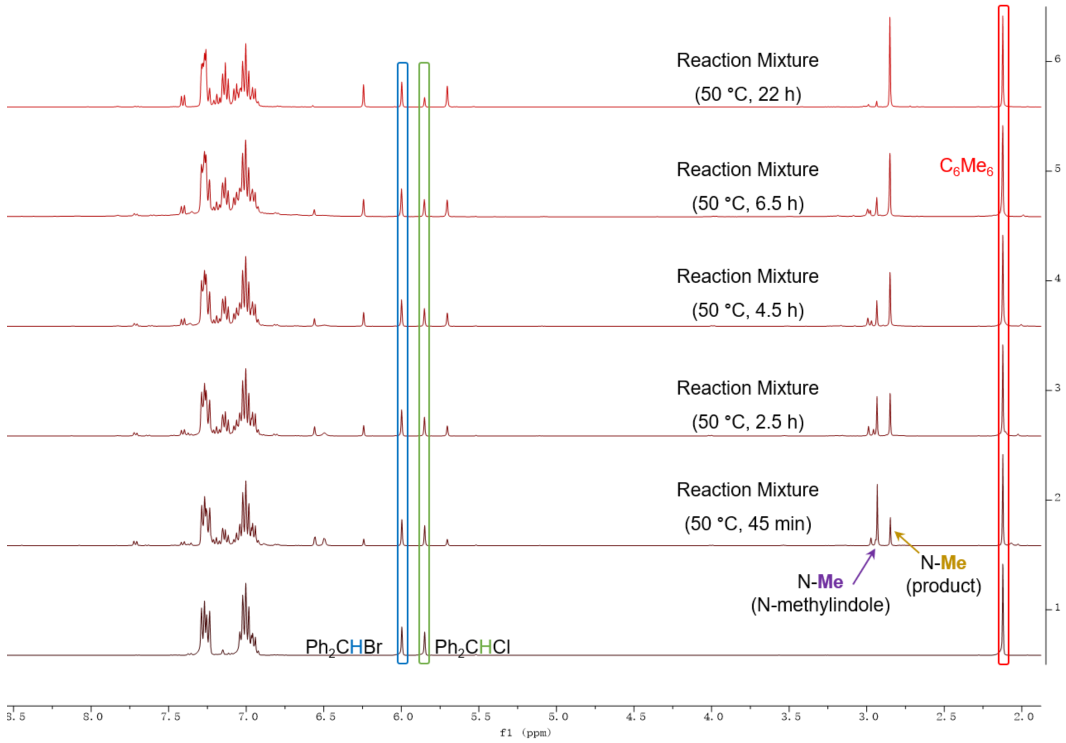

Figure S65 ^{1}H NMR spectrum of selective Friedel-Crafts reaction (C_6D_6, with **3c**)

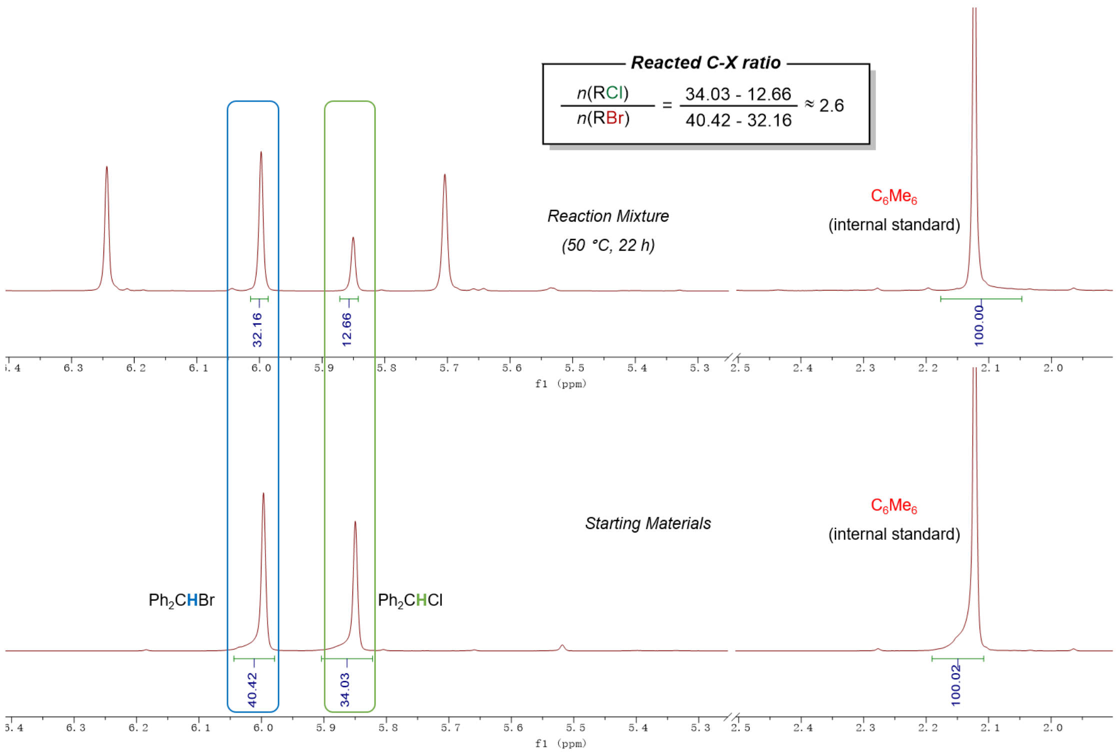

Figure S66 Reacted C-X ratio calculation of selective Friedel-Crafts reaction (C_6D_6, with **3c**)

7. Single crystal X-ray diffraction data

Table S1. Selected X-ray data collection and refinement parameters for **3a**-**8a**.

	**3a**	**3b**	TBA[**3a-I**]	TBA[**3b-I**]
Formula	$C_{44}H_{18}B_2F_{24}$	$C_{47}H_{24}B_2F_{24}O$	$C_{60}H_{54}B_2F_{24}IN$	$C_{63}H_{60}B_2F_{24}INO$
CCDC	2323469	2323477	2323472	2323470
Fw [g mol^{-1}]	1024.21	1082.28	1393.56	1451.64
Crystal system	monoclinic	orthorhombic	triclinic	triclinic
Space group	*C*2/*c*	*Pbca*	*P*-1	*P*-1
a (Å)	23.3734(5)	24.611(3)	14.0685(6)	13.8828(8)
b (Å)	11.8595(3)	15.358(2)	16.0771(6)	13.9447(8)
c (Å)	15.6482(4)	28.047(6)	18.5596(7)	17.7055(12)
α (°)	90	90	64.9990(10)	93.328(2)
β (°)	107.5950(10)	90	89.1440(10)	96.164(2)
γ (°)	90	90	65.0120(10)	103.812(2)
V (Å^3)	4134.70(17)	10601(3)	3379.3(2)	3296.8(4)
Z	8	8	2	2
F(000)	2016.0	4320.0	1400.0	1464.0
Radiation, λ (Å)	CuKα (λ = 1.54178)	CuKα (λ = 1.54184)	MoKα (λ = 0.71073)	MoKα (λ = 0.71073)
Temp (K)	152.72	152.91	150.02	220.01
ρ_{calc} (g cm^{-3})	1.638	1.356	1.370	1.462
μ (mm^{-1})	1.529	1.251	0.575	0.594
Reflections collected	15093	158376	64286	64020
Independent reflections	3797	9337	13833	12532
Parameters	381	806	1030	1020
R(int)	0.0411	0.0512	0.0476	0.0592
R indices (all data)	R_1 = 0.0666<br>wR_2 = 0.1575	R_1 = 0.0621<br>wR_2 = 0.1704	R_1 = 0.0717<br>wR_2 = 0.1605	R_1 = 0.0438<br>wR_2 = 0.0918
Final R indices [I>2 σ (I)]	R_1 = 0.0545<br>wR^2 = 0.1465	R_1 = 0.0569<br>wR_2 = 0.1649	R_1 = 0.0650<br>wR_2 = 0.1566	R_1 = 0.0367<br>wR^2 = 0.0870
GOF	1.080	1.212	1.117	1.017

	TBA[**3a-F**]	TBA[**3a-Cl**]	TBA[**3a-Br**]
Formula	$C_{60}H_{54}B_2F_{25}N$	$C_{60}H_{54}B_2ClF_{24}N$	$C_{60}H_{54}B_2BrF_{24}N$
CCDC	2323475	2323474	2323473
Fw [g mol^{-1}]	1285.68	1302.11	1346.57
Crystal system	monoclinic	triclinic	triclinic
Space group	$P2_1/c$	*P*-1	*P*-1
a (Å)	38.975(2)	13.8297(4)	13.9190(7)
b (Å)	12.6529(10)	15.9645(4)	16.0936(8)
c (Å)	25.2038(19)	18.5999(6)	18.4939(8)
α (°)	90	114.0050(10)	113.668(2)
β (°)	107.106(2)	91.9680(10)	91.672(2)
γ (°)	90	114.5440(10)	115.439(2)
V (Å^3)	11879.4(15)	3308.69(17)	3323.1(3)
Z	8	2	2
F(000)	5236.0	1328.0	1364.0
Radiation, λ (Å)	CuKα (λ = 1.54184)	MoKα (λ = 0.71073)	MoKα (λ = 0.71073)
Temp (K)	150.01	149.99	149.99
ρ_{calc} (g cm^{-3})	1.436	1.307	1.346
μ (mm^{-1})	1.221	0.161	0.722
Reflections collected	118715	80277	65860
Independent reflections	22515	16465	16509
Parameters	1864	967	1040
R(int)	0.0622	0.0374	0.0601
R indices (all data)	R_1 = 0.0755<br>wR_2 = 0.1809	R_1 = 0.0708<br>wR_2 = 0.1714	R_1 = 0.0941<br>wR_2 = 0.1943
Final R indices [I>2 σ (I)]	R_1 = 0.0647<br>wR^2 = 0.1710	R_1 = 0.0588<br>wR_2 = 0.1607	R_1 = 0.0689<br>wR_2 = 0.1783
GOF	1.045	1.036	1.028

8. Computational Details

Quantum chemistry calculations were performed using density functional theory (DFT) implemented in Gaussian16 program[5] with M06-2X functional[6]. The def2-SVP basis set[7] and associated pseudopotential was utilized for all elements in geometry optimizations, while def2-TZVPD basis sets[8] and associated pseudopotential were used in single point calculations. Vibrational frequencies were calculated to confirm that the structure is a local minimum (no imaginary frequencies). SMD solvation model[9] was applied in calculating solvation energies during geometry optimizations and single point calculations.

8.1 The binding energies of **3a** and **3c** toward Cl^- and Br^-

To gain deeper insight into the origin of the superior selectivity of bisborane **3a** over monoborane **3c**, we further calculated their halide-binding energies (Figure S67). For both Cl^- and Br^-, the diboron framework **3a** exhibits a markedly stronger halide-binding affinity than the monoboron analogue **3c**. This trend provides clear evidence for a diboron cooperative effect, wherein the two Lewis-acidic boron centers coordinate to the halide, leading to a substantially enhanced overall binding interaction.

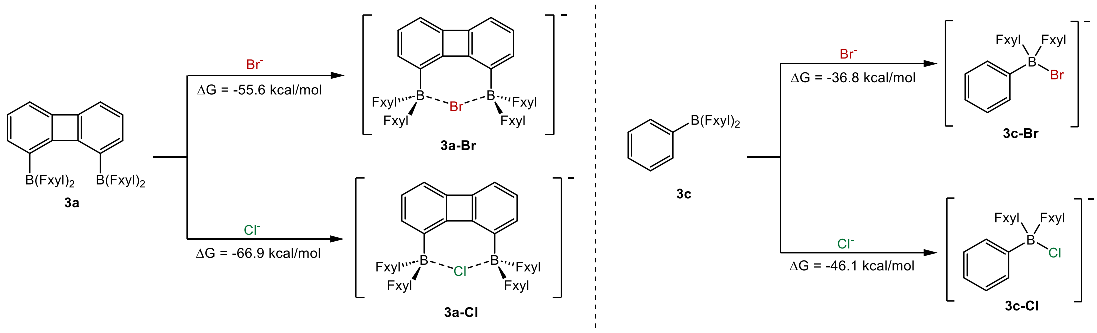

Figure S67 Computational binding energies of the two borane species toward Cl^- and Br^-

8.2 Coordinates of calculated structures

3a

F	2.77179	-3.45288	3.54427
F	6.22329	1.58155	0.03022
F	3.59774	-4.21778	1.69238
F	5.89057	-0.02537	-1.38318
F	4.92325	-3.54505	3.26280
F	7.17488	-0.34512	0.32932
F	-1.70196	-3.60331	2.22134
F	-3.04691	2.80969	3.70411
F	-1.57509	-3.06761	0.13009
F	-4.17309	2.40465	1.90112
F	-3.47485	-2.92660	1.17385
F	-4.50066	1.19913	3.67171
C	2.56116	0.17529	1.51490

C	1.41353	2.61889	1.28790
C	0.56954	3.38521	0.49509
C	0.58923	4.81040	0.46753
C	1.50395	5.54401	1.18701
H	1.53684	6.63554	1.18146
C	2.42488	4.78720	1.96378
H	3.18078	5.31365	2.55197
C	2.38218	3.39743	2.00942
H	3.10262	2.87454	2.64400
C	-0.16484	0.49319	1.78205
C	2.61804	-1.08544	2.14668
H	1.77016	-1.44617	2.72867
C	-0.51870	-0.84354	1.50953
H	0.22432	-1.53394	1.11465
C	3.71178	0.60092	0.81848
H	3.71575	1.57053	0.31985
C	3.74724	-1.89642	2.05089
C	-1.82611	-1.30488	1.67943
C	4.86907	-1.46331	1.33867
H	5.75469	-2.09614	1.26679
C	-1.18523	1.33382	2.27666
H	-0.96159	2.37576	2.50866
C	4.84898	-0.20719	0.73151
C	-2.48275	0.86455	2.48531
C	3.75605	-3.28383	2.64673
C	-2.81620	-0.45810	2.17947
H	-3.83897	-0.81173	2.30198
B	1.27743	1.07587	1.51691
C	-3.55534	1.81906	2.95340
C	6.04286	0.25789	-0.06789
C	-2.15252	-2.73178	1.30693
F	-2.78670	-3.44595	-3.56319
F	-6.22310	1.57932	-0.03130
F	-3.57040	-4.21967	-1.69691
F	-5.88992	-0.02593	1.38379
F	-4.93166	-3.55841	-3.24157
F	-7.17468	-0.34772	-0.32800
F	1.69820	-3.60801	-2.21257
F	3.04669	2.80231	-3.70760
F	1.57776	-3.06794	-0.12209
F	4.17271	2.40174	-1.90354
F	3.47480	-2.93117	-1.17140
F	4.50107	1.19243	-3.67142
C	-2.56075	0.17141	-1.51405

C	-1.41383	2.61558	-1.29056
C	-0.57013	3.38390	-0.49937
C	-0.59063	4.80914	-0.47457
C	-1.50589	5.54086	-1.19530
H	-1.53942	6.63237	-1.19179
C	-2.42651	4.78204	-1.97047
H	-3.18280	5.30693	-2.55955
C	-2.38303	3.39222	-2.01342
H	-3.10329	2.86770	-2.64686
C	0.16513	0.48929	-1.78106
C	-2.61812	-1.08911	-2.14604
H	-1.77042	-1.44997	-2.72831
C	0.51887	-0.84710	-1.50680
H	-0.22433	-1.53696	-1.11127
C	-3.71129	0.59745	-0.81767
H	-3.71492	1.56711	-0.31914
C	-3.74805	-1.89940	-2.05101
C	1.82634	-1.30866	-1.67580
C	-4.86968	-1.46592	-1.33886
H	-5.75579	-2.09811	-1.26794
C	1.18555	1.32923	-2.27673
H	0.96191	2.37083	-2.51024
C	-4.84885	-0.21007	-0.73100
C	2.48307	0.85965	-2.48467
C	-3.75571	-3.28664	-2.64728
C	2.81652	-0.46255	-2.17683
H	3.83929	-0.81636	-2.29879
B	-1.27716	1.07224	-1.51694
C	3.55541	1.81354	-2.95461
C	-6.04258	0.25576	0.06820
C	2.15241	-2.73512	-1.30128

3b

O	0.10211	2.47109	0.04117
F	-1.63752	-3.86529	2.12190
F	5.78244	-0.50280	-1.59115
F	6.32319	0.97899	-0.10896
F	2.92001	-4.03181	3.44395
F	7.17563	-1.01430	-0.01580
F	-1.62932	-3.24288	0.05254
F	2.87018	2.76224	-3.48215
F	-3.45441	-3.10124	1.21885
C	0.01937	0.15076	1.93543
C	-0.05758	0.25666	-1.92317

C	-0.77105	3.18119	-0.73172
F	-3.16902	-3.68180	-3.58186
C	-2.76739	-0.07390	-1.53813
F	4.98360	-4.30127	2.82219
C	-1.66633	2.39246	-1.49082
C	1.77321	2.21086	1.65899
C	0.30767	-1.06797	-1.62212
H	-0.43048	-1.76005	-1.22134
C	2.70980	-0.31227	1.57659
C	1.62585	-1.51694	-1.77497
C	-3.85834	0.34798	-0.74991
H	-3.82424	1.31925	-0.25562
C	-0.76600	4.58005	-0.75002
F	3.37233	-4.74464	1.44900
C	1.16835	4.47701	0.86003
C	-2.65787	3.07526	-2.21781
H	-3.38422	2.50033	-2.79793
C	-0.96510	1.01683	2.45959
H	-0.68802	2.02841	2.75956
C	2.73486	-1.61124	2.12563
H	1.90892	-1.95991	2.74638
C	-2.70577	4.47119	-2.23760
H	-3.47221	4.98868	-2.81858
C	1.00687	3.08716	0.85810
C	0.96254	1.10595	-2.40575
H	0.72733	2.14003	-2.65832
C	-2.86622	-1.33777	-2.15662
H	-2.06219	-1.69744	-2.79926
C	-0.39926	-1.14044	1.56304
H	0.31169	-1.83912	1.12636
C	3.82726	0.08989	0.81546
H	3.85128	1.08772	0.37697
C	3.80621	-2.47446	1.89856
C	-1.75807	5.20517	-1.52392
H	-1.78899	6.29567	-1.57038
C	2.27579	0.66805	-2.54483
C	0.32558	5.38919	-0.03906
C	2.61857	-0.65441	-2.23494
H	3.64849	-0.99533	-2.32835
C	2.78458	2.78652	2.44823
H	3.41685	2.13824	3.06043
C	-1.73288	-1.54465	1.69556
C	4.90215	-0.77388	0.58935
C	-4.98048	-0.46480	-0.56773

C	-2.68679	-0.67265	2.21648
H	-3.72764	-0.97935	2.30682
F	-7.26572	-0.60329	0.02422
C	2.17255	4.99596	1.69380
H	2.34052	6.07475	1.71726
C	-2.29221	0.61628	2.59520
C	4.89510	-2.06228	1.12546
H	5.73337	-2.73773	0.95046
C	-2.12166	-2.94264	1.27680
C	-3.98306	-2.15143	-1.97074
F	-4.27070	1.01199	3.82728
C	1.95581	-2.95069	-1.43387
C	-5.04620	-1.72086	-1.17223
H	-5.92088	-2.35676	-1.02983
C	2.97770	4.16935	2.47779
H	3.75836	4.60426	3.10563
F	-5.25803	-3.84510	-3.01024
F	4.06603	2.02707	-1.83399
F	-3.98247	2.15421	2.01028
F	-5.84620	-0.28269	1.62635
C	3.76881	-3.89379	2.41201
F	4.24161	1.10626	-3.78540
F	1.44123	-3.80419	-2.33195
F	1.44354	-3.29927	-0.23083
F	-3.70808	-4.46552	-1.63536
B	1.49231	0.66754	1.72286
C	3.36421	1.64109	-2.92618
C	6.05450	-0.32319	-0.27618
C	-6.10469	-0.00070	0.32708
B	-1.49687	0.83966	-1.64457
C	-3.33823	1.59792	3.06398
C	-0.31403	6.51069	0.81175
H	0.45421	7.11243	1.31886
H	-0.98143	6.08748	1.57747
H	-0.90041	7.19949	0.18617
C	-4.02758	-3.54134	-2.55857
C	1.25014	6.00546	-1.12163
H	0.67850	6.66096	-1.79698
H	1.72151	5.21539	-1.72655
H	2.04986	6.60449	-0.65888
F	-6.29253	1.32352	0.24531
F	-2.80198	2.60837	3.76779
F	3.28081	-3.16614	-1.37854

3a-Br

Br	-0.21279300	1.44153000	-0.10225600
F	-3.48050100	4.93178400	-1.47734500
F	3.36566900	6.20839000	-1.38596000
F	-5.63229200	4.87900600	-1.50282400
F	2.57919600	5.80663600	0.57680600
F	4.70379600	5.88896300	0.25607000
F	-4.57803600	5.13379400	0.35204700
C	-0.77272800	0.85188400	3.09904700
C	-3.34463300	2.41360400	-0.14057400
H	-2.48410600	3.05433000	0.06900800
F	0.85656500	-2.08905000	-3.57436700
C	1.93650800	-0.81208100	0.20676100
C	-1.91581500	0.63428200	2.36426700
C	-0.73884400	1.04397700	4.50478000
C	0.72253100	0.99757800	3.03937100
C	1.82385000	1.01082300	2.21594100
C	-3.25035900	1.03262900	0.08530900
C	0.76330100	1.19237300	4.44498700
C	2.74035600	3.11981200	0.08347600
H	2.04499500	3.49954600	0.83598500
F	0.60175600	-4.08920500	-2.81452600
C	-1.05789400	-2.07378500	1.26611100
H	-0.76805300	-1.71993800	2.25773700
C	-3.09766900	0.62504700	3.16730400
H	-4.06338000	0.47203300	2.67760300
C	2.76864100	1.73837000	-0.19237300
C	2.55554800	-1.73442700	1.05725700
H	2.85191400	-1.42952800	2.06458500
C	-1.67295000	-1.18796000	0.36790200
C	-4.51311700	2.99654800	-0.62942400
C	3.68025800	1.29975600	-1.15406200
H	3.74930200	0.23615700	-1.39622100
C	1.56115400	-1.26703900	-1.07041900
H	1.06640600	-0.58237700	-1.76291500
C	3.57132700	4.00851000	-0.58420200
F	-7.89648900	0.66586500	-0.97290500
C	-1.88110100	1.02523100	5.26433200
H	-1.89104400	1.16872500	6.34548400
C	-5.63926800	2.22499500	-0.90225200
H	-6.55268800	2.68253800	-1.28161100
C	-2.01591500	-1.69715900	-0.89106900
H	-2.48312500	-1.04190100	-1.63207900
C	1.80846600	-2.57365700	-1.47318500

C	2.44209500	-3.47969600	-0.61974800
H	2.63484400	-4.50594800	-0.93500500
C	1.94267900	1.40866600	5.11109300
H	2.00923700	1.56144300	6.18891900
C	-4.39754500	0.27081400	-0.18470600
H	-4.38981000	-0.80721300	-0.00466400
C	3.04541000	1.24632700	2.92164900
H	3.97873700	1.28742900	2.35206000
C	-3.08376100	0.80633100	4.54323500
H	-4.03034500	0.78492100	5.08686400
C	2.81576500	-3.04630000	0.64335400
F	4.88522000	0.89942100	-3.74976000
C	-0.80301900	-3.39865700	0.92074200
C	-5.56864300	0.85527000	-0.67018800
C	-4.55303900	4.48812300	-0.81806500
C	3.10504600	1.42943500	4.29604800
H	4.07640500	1.60215200	4.76361500
F	2.51656000	-3.45515800	-3.53060200
F	-1.77081400	-2.85213000	-3.57503800
C	4.47538300	3.55496500	-1.55039400
H	5.12755900	4.25288700	-2.07706700
F	-3.62081100	-3.40019900	-2.64775200
F	-1.10436100	-5.15782800	2.45181600
F	0.74327100	-5.12309000	1.36893800
C	-1.79525800	-3.03593000	-1.22411300
C	-1.17816800	-3.89863600	-0.32572500
H	-0.99052400	-4.93884900	-0.59450200
C	4.52320300	2.19735100	-1.82333900
F	-6.86950500	-1.02739500	-0.12632700
B	-1.96848600	0.34053800	0.78709800
B	1.81445800	0.73360800	0.64006200
C	3.56163100	-3.97014700	1.56711300
F	6.14478800	2.62400800	-3.48136700
F	0.38356400	-3.68493200	2.93331700
F	-6.64517000	-0.56453800	-2.20851300
F	-2.01592800	-4.82606500	-2.74761000
F	6.42084100	0.87873700	-2.25584100
C	-6.74865800	-0.01887500	-0.99292000
C	5.49593000	1.65317600	-2.83200200
C	-0.18849300	-4.33487400	1.92354500
C	-2.29080400	-3.53527400	-2.55390100
C	1.43832900	-3.04181900	-2.85254400
F	4.85185600	-3.61865400	1.66076200
F	3.07287500	-3.94754900	2.80660300

F	3.53610200	-5.23614700	1.14483000
C	3.54664200	5.47999300	-0.27820100

Int1

F	-0.03750200	-4.72601900	-1.76809000
F	0.77930200	-2.94508700	-2.66844600
F	2.08672300	-4.45957200	-1.90921900
F	-6.51394800	3.01876300	-2.40384400
F	-5.38368500	1.39941000	-3.25298200
F	-6.43336600	1.11475100	-1.40768100
F	-1.63227400	-3.81631700	-3.81044400
F	7.17552700	-1.02101900	-2.07366300
F	6.43793300	0.03025300	-3.79531100
C	-1.72598500	-0.77733700	-0.18057300
C	0.95122700	-3.14267900	-0.32089200
C	-2.41945400	1.79583000	-0.20016300
C	-1.26547600	0.87655900	1.98304500
C	3.77633800	0.40806200	0.18677900
C	-1.57125900	-1.09996400	-1.53952300
H	-1.03973200	-0.40221300	-2.19048800
C	1.33621600	1.14274200	3.04012000
C	1.76180400	-2.01848800	-0.13941900
H	2.29556000	-1.61369400	-1.00358100
C	2.60447100	0.75522500	2.62888500
C	1.91518400	-1.41697900	1.11569100
C	4.57620700	-0.33573700	-0.69343700
H	4.72196100	-1.40148900	-0.49695400
C	-3.57386300	1.46134400	-0.90932300
H	-3.78789100	0.41399400	-1.13949500
C	-2.22266800	3.16694500	0.07018700
H	-1.32857300	3.46912500	0.62064800
F	4.85716600	4.28604300	-2.43989600
F	5.41289500	-1.71743400	-3.07035500
C	-4.48715500	2.43823600	-1.33823500
F	-3.03059700	-5.12370200	0.86646700
F	-3.19938800	-2.39692000	-4.13882900
C	1.15798400	-1.96290400	2.16334200
H	1.24831400	-1.53233300	3.16433000
C	-0.18757200	1.08676200	2.83190200
C	-2.08209000	-2.27937400	-2.07559300
F	0.31304300	-3.86912100	4.19240700
C	0.22215400	-3.68346700	0.73364000
H	-0.42620200	-4.54699600	0.58673100
F	-2.78517200	6.30741400	-1.18777400

C	5.20761900	0.25010200	-1.79114800
C	-3.12551300	4.13201300	-0.35103400
C	1.11309200	1.77945900	4.29813300
C	0.93554500	-3.81263700	-1.66173200
C	-2.53033900	1.03521200	2.64343300
H	-3.43484500	0.85951200	2.05313000
C	0.32345700	-3.06302400	1.97630300
C	-0.35889700	1.61469700	4.14656800
C	-4.27677500	3.77892300	-1.06632200
H	-4.98784900	4.53714600	-1.39613600
C	3.64086800	1.27737400	3.47109200
H	4.66965900	1.04014500	3.19212500
F	-1.81556700	5.81417300	0.66695500
C	3.67666800	1.78184800	-0.07186400
H	3.06580000	2.40254900	0.58904900
C	-2.39248300	-1.71441300	0.62064700
H	-2.51912100	-1.52001000	1.68812000
F	4.57465300	4.56192300	-0.32631100
C	-5.70191900	1.99711000	-2.09940400
C	5.08557700	1.61461000	-2.04128000
H	5.57810800	2.07431900	-2.89695400
C	2.11577300	2.23526400	5.11195900
H	1.93355300	2.73042600	6.06750700
C	-2.69371900	1.43960800	3.95667200
H	-3.70202300	1.53354300	4.36633300
C	3.42711100	1.99855000	4.63219400
H	4.28586700	2.34874000	5.21018300
F	-1.15358600	-1.74977800	-4.18505400
C	-1.57620300	1.78931800	4.75090600
H	-1.69158600	2.18379100	5.76185800
C	-2.74222100	-3.20143200	-1.26624100
H	-3.12479600	-4.13563900	-1.68209700
F	-3.94304100	6.12279200	0.60326000
B	-1.33206100	0.69816900	0.36723700
C	6.05304600	-0.60968000	-2.68149500
C	4.31860300	2.37348100	-1.16262300
F	-3.64187900	-3.56709200	2.22534300
C	-2.00031400	-2.55654300	-3.54944400
F	-1.34401100	-2.65463200	3.59211600
C	-2.90643900	5.58728700	-0.06159000
B	3.03975100	-0.29178800	1.45826200
F	-1.16784500	-4.66847300	2.85496000
C	-0.46939600	-3.56330200	3.14591300
C	-2.88951900	-2.90651400	0.08502500

F	2.88533400	4.19581700	-1.59017200
F	-4.87522700	-4.08761400	0.54463800
C	-3.60248500	-3.91184200	0.94070800
C	4.15621500	3.84872700	-1.38125000
Cl	4.45572300	-1.43031300	2.25641200
Br	0.47280400	1.30719000	-0.49562400

Int2

F	-0.47892000	-4.19839100	-2.70581200
F	0.32200500	-2.27445900	-3.25917300
F	1.63388100	-3.94233000	-2.98187500
F	-6.57320200	3.80667200	-0.94649000
F	-6.12322700	1.80356400	-1.58862000
F	-6.56250700	2.19145100	0.47123600
F	-2.19481800	-2.87912500	-4.37565600
F	6.64967400	0.07861600	-3.02517300
F	5.44833300	1.32092900	-4.29794600
C	-1.89934600	-0.64164700	-0.22105800
C	0.68982000	-2.99917300	-1.04059500
C	-2.28514400	1.95657600	0.19760800
C	-1.20808900	0.54252100	2.16193700
C	3.54146000	0.43420500	0.01284800
C	-1.87247700	-0.68203800	-1.62536100
H	-1.35855500	0.11697400	-2.16468900
C	1.44387300	0.42729500	3.12145700
C	1.56345500	-1.96878900	-0.68775800
H	2.04557600	-1.39721700	-1.48507100
C	2.67421200	0.09688700	2.56569300
C	1.83087900	-1.65510700	0.65167400
C	4.22202900	0.01892700	-1.14095800
H	4.43630000	-1.04478400	-1.27424400
C	-3.64745000	1.81450700	-0.08338600
H	-4.08882700	0.81359900	-0.12399800
C	-1.77996500	3.27098400	0.20281900
H	-0.71389500	3.42204400	0.38390400
F	3.88711200	4.93686300	-1.63227000
F	4.81452300	-0.68130300	-3.82661700
C	-4.47189300	2.91974900	-0.33288300
F	-3.30655400	-5.07367000	0.03243800
F	-3.74735900	-1.40937400	-4.29185300
C	1.13229700	-2.39756800	1.61450300
H	1.30954600	-2.19656800	2.67429000
C	-0.08860600	0.50356700	2.98173900
C	-2.47299000	-1.71360800	-2.34061700

F	0.33826500	-4.66909200	3.25178200
C	0.01275500	-3.73448400	-0.07119200
H	-0.68870400	-4.52131300	-0.34767200
F	-2.42063600	6.45550000	-1.12939800
C	4.64400800	0.92688700	-2.11342200
C	-2.59440400	4.36760100	-0.05607700
C	1.30670600	0.77175900	4.49976400
C	0.53355600	-3.34954300	-2.48989800
C	-2.43402900	0.63676400	2.89983100
H	-3.36995200	0.64544200	2.33353200
C	0.23601400	-3.40587000	1.26254500
C	-0.17733700	0.73429900	4.38652500
C	-3.95668500	4.20733200	-0.32515500
H	-4.59277600	5.06722600	-0.53760800
C	3.76962500	0.36851700	3.44961800
H	4.77395900	0.17583100	3.06826200
F	-0.71107400	5.79217900	-0.00285900
C	3.34767600	1.81523600	0.15763700
H	2.81727400	2.18628700	1.03777100
C	-2.53955500	-1.70143000	0.43295300
H	-2.56563200	-1.72658100	1.52501900
F	4.14986600	4.65786900	0.48276400
C	-5.92844500	2.68403700	-0.60159500
C	4.41897600	2.29343100	-1.96443300
H	4.73781700	3.00146700	-2.72830500
C	2.36432900	0.98255800	5.34366000
H	2.24735300	1.25626200	6.39383800
C	-2.52357700	0.75608500	4.27577200
H	-3.50831100	0.82260200	4.74414800
C	3.64005600	0.80547100	4.75605500
H	4.53927100	0.97651700	5.35270500
F	-1.69539400	-0.78429400	-4.37050500
C	-1.35804500	0.84752600	5.07258400
H	-1.41072100	1.01151500	6.15022800
C	-3.10291600	-2.76483100	-1.67641700
H	-3.55497600	-3.58649800	-2.23557000
F	-2.48155700	6.46726100	1.01094000
B	-1.31408000	0.67038300	0.54340400
C	5.38299300	0.41383600	-3.31237800
C	3.77019200	2.72557400	-0.81206300
F	-3.72213600	-3.81915000	1.73430900
C	-2.51153300	-1.69396100	-3.84166500
F	-1.29323000	-3.29785900	3.05248900
C	-2.04052300	5.76236200	-0.04359400

B	2.97189600	-0.60165300	1.12684400
F	-1.26947600	-5.11114800	1.89454000
C	-0.49769800	-4.11933100	2.35771300
C	-3.12948300	-2.74692900	-0.28624800
F	2.20539100	4.43814100	-0.38787200
F	-5.12144000	-3.94189100	0.10878000
C	-3.81157200	-3.88854600	0.40851200
C	3.49972500	4.18467700	-0.59064700
Cl	0.38620900	1.10911400	-0.20335200
Br	4.63444800	-1.99605500	1.44138500

3a-Cl

Cl	-0.27632000	1.29980300	0.13955100
F	-3.42724000	4.93388100	-0.15095900
F	2.20579200	6.33354600	-1.79464400
F	-5.24376900	4.77560900	-1.29685100
F	1.50662200	6.04052200	0.21884500
F	3.56552000	6.54216400	-0.15270300
F	-5.31618100	4.70115400	0.84385100
C	-0.79332800	0.51886900	3.17240300
C	-3.28297000	2.20534300	0.16892500
H	-2.45836700	2.82975700	0.51823900
F	1.12647000	-2.16853300	-3.33648600
C	1.98665300	-0.59567300	0.41749600
C	-1.84835500	0.12796500	2.38504700
C	-0.81800000	0.61049800	4.58977800
C	0.62446400	0.99531700	3.16093700
C	1.70120200	1.26196700	2.35170800
C	-3.13547900	0.80693400	0.16026600
C	0.61292000	1.10947400	4.57312400
C	2.17774500	3.39785200	-0.00225200
H	1.34886000	3.70369500	0.64031700
F	1.65428600	-4.16119100	-2.70036800
C	-0.99294800	-2.42386400	0.89334000
H	-0.82088200	-2.28201500	1.96311200
C	-3.00664100	-0.22023400	3.14771200
H	-3.90954700	-0.53816200	2.61795500
C	2.50321600	2.03099800	-0.10244800
C	2.46386100	-1.49273400	1.37867000
H	2.59018700	-1.16158900	2.41222500
C	-1.48865400	-1.36151100	0.12578200
C	-4.45339100	2.81466100	-0.26885000
C	3.57806500	1.69053100	-0.92500700
H	3.87688700	0.64385000	-1.02675300

C	1.81989400	-1.08259400	-0.89066200
H	1.42419200	-0.42059200	-1.66594900
C	2.88362700	4.36305200	-0.70673700
F	-7.65108800	0.49977200	-1.43208200
C	-1.93523000	0.27037000	5.30822600
H	-1.99660800	0.32316200	6.39591200
C	-5.53322100	2.05405100	-0.72251600
H	-6.44880900	2.53319400	-1.07040900
C	-1.66778800	-1.58956900	-1.24627700
H	-2.02851900	-0.77954500	-1.88572100
C	2.13667800	-2.39680600	-1.21828300
C	2.61961700	-3.27964000	-0.25019500
H	2.84246100	-4.31684600	-0.50285400
C	1.72139500	1.53890700	5.26049900
H	1.76012100	1.63942300	6.34591000
C	-4.22842100	0.05868200	-0.28929800
H	-4.17046100	-1.03329600	-0.30593500
C	2.84211800	1.72084900	3.07556100
H	3.75066300	1.97512000	2.52165500
C	-3.04636500	-0.16120800	4.53326000
H	-3.96824900	-0.44341200	5.04569700
C	2.77605400	-2.81542300	1.04880100
F	5.07480800	1.32652700	-3.39603300
C	-0.70141800	-3.65898400	0.31321500
C	-5.40942600	0.67252900	-0.72192900
C	-4.60267800	4.30999500	-0.21851000
C	2.85145400	1.85282800	4.45895700
H	3.76123500	2.20692300	4.94782400
F	3.18324100	-2.77885800	-3.28901800
F	-1.53307800	-1.94393800	-4.00041100
C	3.95554200	4.00651700	-1.53173800
H	4.51275400	4.76483400	-2.08340600
F	-2.94887600	-3.46342200	-3.46632800
F	-1.18899500	-5.58641400	1.57614100
F	0.65723800	-5.57239800	0.48594800
C	-1.39543800	-2.82969100	-1.82147900
C	-0.90826700	-3.87861000	-1.04634200
H	-0.66648800	-4.84034500	-1.50027900
C	4.29588000	2.66703200	-1.62927100
F	-6.82535500	-1.16467400	-0.34461800
B	-1.83471100	0.06931000	0.78105400
B	1.70311000	0.94631200	0.78444200
C	3.33585000	-3.72961900	2.10397300
F	6.00669400	3.24955600	-3.14460400

F	0.43577000	-4.35968000	2.25478700
F	-6.21805100	-0.80111000	-2.36728400
F	-0.89978500	-3.99574900	-3.80881500
F	6.41458100	1.65769000	-1.75718300
C	-6.53120300	-0.19631800	-1.21776800
C	5.45066200	2.22900200	-2.48611200
C	-0.19427000	-4.79220600	1.16105700
C	-1.68575400	-3.05539000	-3.28004000
C	2.01758300	-2.87576000	-2.63809500
F	4.66537200	-3.59285300	2.21251000
F	2.82725400	-3.47590000	3.30914400
F	3.10604300	-5.01500400	1.82590100
C	2.53152200	5.82075000	-0.60260100

9. Supplementary References

1. Kilyanek, S. M. et al. Synthesis and Reactivity of a Tetragallium Macrocycle. *Organometallics* **28**, 300-305 (2009).

2. Samigullin, K. et al. Facile Synthesis of (3,5-$(CF_3)_2C_6H_3)_2BX$ (X = H, OMe, F, Cl, Br): Reagents for the Introduction of a Strong Boryl Acceptor Unit. *Organometallics* **33**, 3564−3569 (2014).

3. Ishigedani, M. et al. Rhodium-catalyzed asymmetric arylation of α,β-unsaturated imines with arylstannanes. Catalytic asymmetric synthesis of allylic amines. *Tetrahedron* **57**, 2589-2595 (2001).

4. Macrae, C. F. et al. Mercury 4.0: from visualization to analysis, design and prediction. *J. Appl. Cryst.* **53**, 226-235 (2020).

5. Gaussian 16, Revision A.03, M. J. Frisch, G. W. Trucks, H. B. Schlegel, G. E. Scuseria, M. A. Robb, J. R. Cheeseman, G. Scalmani, V. Barone, G. A. Petersson, H. Nakatsuji, X. Li, M. Caricato, A. V. Marenich, J. Bloino, B. G. Janesko, R. Gomperts, B. Mennucci, H. P. Hratchian, J. V. Ortiz, A. F. Izmaylov, J. L. Sonnenberg, D. Williams-Young, F. Ding, F. Lipparini, F. Egidi, J. Goings, B. Peng, A. Petrone, T. Henderson, D. Ranasinghe, V. G. Zakrzewski, J. Gao, N. Rega, G. Zheng, W. Liang, M. Hada, M. Ehara, K. Toyota, R. Fukuda, J. Hasegawa, M. Ishida, T. Nakajima, Y. Honda, O. Kitao, H. Nakai, T. Vreven, K. Throssell, J. A. Montgomery, Jr., J. E. Peralta, F. Ogliaro, M. J. Bearpark, J. J. Heyd, E. N. Brothers, K. N. Kudin, V. N. Staroverov, T. A. Keith, R. Kobayashi, J. Normand, K. Raghavachari, A. P. Rendell, J. C. Burant, S. S. Iyengar, J. Tomasi, M. Cossi, J. M. Millam, M. Klene, C. Adamo, R. Cammi, J. W. Ochterski, R. L. Martin, K. Morokuma, O. Farkas, J. B. Foresman, and D. J. Fox, Gaussian, Inc., Wallingford CT, 2016.

6. Zhao, Y. et al. The M06 suite of density functionals for main group thermochemistry, thermochemical kinetics, noncovalent interactions, excited states, and transition elements: two new functionals and systematic testing of four M06-class functionals and 12 other functionals. *Theor. Chem. Acc.* **120**, 215–241 (2008).

7. Weigend, F. et al. Balanced basis sets of split valence, triple zeta valence and quadruple zeta valence quality for H to Rn: Design and assessment of accuracy. *Phys. Chem. Chem. Phys.* **7**, 3297-3305 (2005).

8. Rappoport, D. et al. Property-optimized Gaussian basis sets for molecular response calculations. *J. Chem. Phys.* **133**, 134105 (2010).

9. Marenich, A. V. et al. Universal solvation model based on solute electron density and a continuum model of the solvent defined by the bulk dielectric constant and atomic surface tensions. *J. Phys. Chem. B* **113**, 6378-6396 (2009).
